# A dinuclear ruthenium(ii) phototherapeutic that targets duplex and quadruplex DNA†
†Electronic supplementary information (ESI) available: Additional references to relevant primary literature for this research:^90^–^98^ experimental methods and data, cell studies details, computational methods, tables and figures. CCDC 1876685. For ESI and crystallographic data in CIF or other electronic format see DOI: 10.1039/c8sc05084h


**DOI:** 10.1039/c8sc05084h

**Published:** 2019-02-18

**Authors:** Stuart A. Archer, Ahtasham Raza, Fabian Dröge, Craig Robertson, Alexander J. Auty, Dimitri Chekulaev, Julia A. Weinstein, Theo Keane, Anthony J. H. M. Meijer, John W. Haycock, Sheila MacNeil, James A. Thomas

**Affiliations:** a Department of Chemistry , University of Sheffield , Brook Hill , Sheffield , S3 7HF , UK . Email: james.thomas@sheffield.ac.uk ; Tel: +44 (0)114 222 9325; b Materials Science & Engineering , University of Sheffield , Mappin St , Sheffield S1 3JD , UK . Email: j.w.haycock@sheffield.ac.uk ; Email: s.macneil@sheffield.ac.uk

## Abstract

With the aim of developing a sensitizer for photodynamic therapy, a previously reported luminescent dinuclear complex that functions as a DNA probe in live cells was modified to produce a new iso-structural derivative containing Ru^II^(TAP)_2_ fragments (TAP = 1,4,5,8-tetraazaphenanthrene). The structure of the new complex has been confirmed by a variety of techniques including single crystal X-ray analysis. Unlike its parent, the new complex displays Ru → L-based ^3^MLCT emission in both MeCN and water. Results from electrochemical studies and emission quenching experiments involving guanosine monophosphate are consistent with an excited state located on a TAP moiety. This hypothesis is further supported by detailed DFT calculations, which take into account solvent effects on excited state dynamics. Cell-free steady-state and time-resolved optical studies on the interaction of the new complex with duplex and quadruplex DNA show that the complex binds with high affinity to both structures and indicate that its photoexcited state is also quenched by DNA, a process that is accompanied by the generation of the guanine radical cation sites as photo-oxidization products. Like the parent complex, this new compound is taken up by live cells where it primarily localizes within the nucleus and displays low cytotoxicity in the absence of light. However, in complete contrast to [{Ru^II^(phen)_2_}_2_(tpphz)]^4+^, the new complex is therapeutically activated by light to become highly phototoxic toward malignant human melanoma cell lines showing that it is a promising lead for the treatment of this recalcitrant cancer.

## Abstract 2

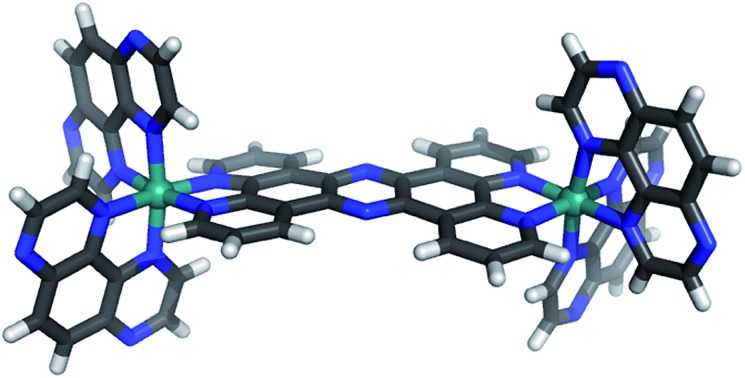
A previously reported dinuclear DNA imaging probe has been converted into a phototherapeutic through the incorporation of Ru^II^(TAP)_2_ fragments (TAP = 1,4,5,8-3 tetraazaphenanthrene).

## Introduction

A promising and emerging therapeutic regime for the treatment of cancer is photo-dynamic therapy (PDT).^1^,^2^ In this approach a photo-excitable sensitizer molecule is used to generate highly reactive species, most commonly singlet oxygen, ^1^O_2_, *in situ*. Whilst a number of PDT sensitizers have reached clinical trials, there are still very few successful commercial examples available; those that are currently employed frequently display poor water solubility and do not target cancer cells with high specificity, leading to generalized photosensitivity and off-target toxic side effects.^3^ Moreover, these agents are often challenging to synthesize and so can be costly. Furthermore, whilst the generation of ^1^O_2_ is essentially a photocatalytic process, one of the drawbacks of this approach is that many tumours are hypoxic, which can restrict the efficacy of PDT in these circumstances.

One alternative to conventional PDT involves the use of water-soluble polypyridyl *d*^6^–metal complexes as sensitizers as, on photo-excitation with visible light, these complexes often possess long-lived triplet states. Because their synthesis from specific metal ions and ligands is essentially modular, the photophysical and biophysical properties of these complexes are easily tuned.^4^–^8^ and Ru^II^ complexes have attracted particular attention as sensitizers.^9^–^18^

**Scheme 1:**
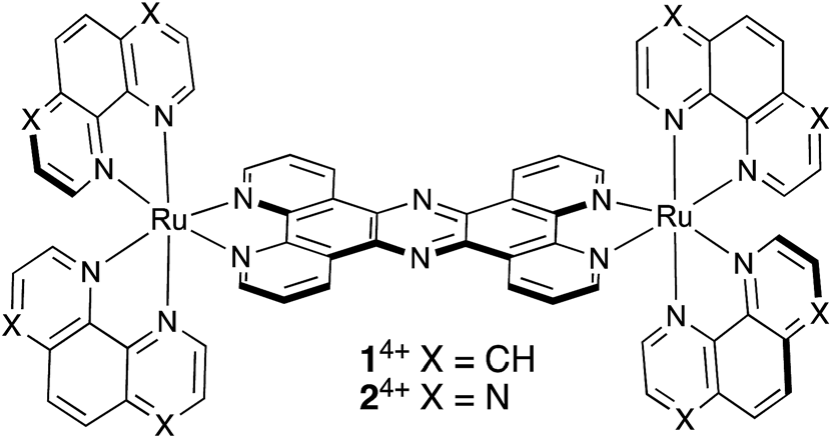
Structures of **1**^4+^ and **2**^4+^.

For similar reasons, this class of compounds has also been investigated as probes for optical microscopy. A case in point is the probe described in previous reports by the Thomas group. We have identified a dinuclear Ru^II^ complex, [{(phen)_2_Ru}_2_(tpphz)]^4+^, **1**^4+^,^19^,^20^ (phen = 1,10-phenanthroline, tpphz = tetrapyridophenazine), Scheme 1, whose biophysical properties are related to the ubiquitous [Ru(bpy)_2_(dppz)]^2+^ “light-switch” complex (bpy = 2,2′-bipyridyl, dppz = dipyridophenazine).^21^,^22^ Like the parent compound, **1**^4+^ is virtually non-emissive in aqueous solution but – on high-affinity binding to DNA – it displays the familiar bright, ^3^MLCT-based, luminescence. Strikingly, the emission of **1**^4+^ is dependent on DNA structure: whereas binding to canonical duplex DNA induces NIR emission centred at ∼680 nm, binding to G-rich, quadruplex folded, human telomere sequence – (3′-TTAGGG-5′)_*n*_ – leads to emission at 650 nm and a greater enhancement of emission (×150 *vs.* x60).^19^,^23^ Detailed subsequent studies, including co-localization with nucleic acid stains and TEM imaging, confirmed that **1**^4+^ is taken up by live cells, where it localizes within the nucleus imaging heterochromatin duplex and quadruplex structures.^24^

This observation is particularly significant, as quadruplex structures have been hypothesized to have roles in a range of biological processes, including modulation of gene expression, epigenetics, nucleating of DNA replication, and genetic disease.^25^–^30^ Consequently, this has led to a plethora of research into these non-canonical structures, including work on telomeres. Single stranded telomere sequences are found at the end of chromosomes and, as outlined above, are known to form quadruplexes. Telomeres are associated with defining the Hayflick limits for cell division: each cell division results in shortening of the telomere sequence, until a critical length is reached and senescence is triggered. Given these facts, it is perhaps not surprising that telomere length maintenance, leading to cell immortalization, is an almost ubiquitous phenomenon in cancer cells.^31^,^32^ For this reason, selective methods to facilitate the attrition of telomere length in cancer cells, which often involve stabilization of quadruplex structures, have been sought.^32^

As **1**^4+^ binds to both quadruplex and duplex structures within live cells, a phototoxic analogue would function as a sensitizer for PDT that could additionally enhance telomere attrition rates. To carry out this transformation we changed the ancillary ligands attached to the central Ru^II^(tpphz)Ru^II^ moiety.

Extensive studies have demonstrated that the substitution of conventional bpy or phen ancillary ligands with the electron deficient ligand tetraazaphenanthrene, TAP, produces a complex with strikingly different photo-excited state properties.^33^–^37^ For example, unlike the parent light-switch complex, [Ru(TAP)_2_(dppz)]^2+^ is luminescent in water but is quenched by binding to DNA. This is due to photo-excitation into a Ru^II^ → TAP ^3^MLCT excited state which photo-oxidizes guanine sites when intercalated into duplex DNA. In cell free studies, a related dinuclear complex was shown to be a more efficient at photo-damaging oligonucleotides containing G than [Ru(TAP)_2_(dppz)]^2+^ and was particularly effective at creating photo-adducts when bound to quadruplex folded DNA.^38^ However, cell studies on such systems are much less developed. Whilst it is known that, even after 24 hours exposure, [Ru(TAP)_2_(dppz)]^2+^ solely localizes in the cytoplasm of HeLa cells,^39^ the Keyes group has demonstrated that covalent attachment of a suitable signal peptide to a [Ru(TAP)_2_(bpy)]^2+^ unit leads to a construct capable of targeting cell nuclei. Although this hybrid system possesses considerable phototoxicity, it is produced on a small scale, using only 10 mg of metal complex starting material.^40^ Recently, the Elias group have reported preliminary studies on mononuclear complexes containing the Ru^II^(TAP)_2_ moiety coordinated to a quadruplex targeting ligand. Whilst this complex is phototoxic, so is its Ru^II^(phen)_2_ analogue, suggesting that the observed phototoxicity of both complexes may be due to classic singlet oxygen sensitization.^41^

This report concerns an essentially iso-structural derivative of **1**^4+^ containing Ru^II^(TAP)_2_ fragments. In this study we describe the synthesis, characterization, and crystal structure of this new complex [{Ru(TAP_2_)}_2_(tpphz)]^4+^, **2**^4+^, which can be isolated in good yields.

Experimental and computational studies reveal that **2**^4+^ displays a Ru → TAP MLCT excited state and cell free studies confirm that the complex binds to both duplex and quadruplex DNA. Photophysical studies confirm that the complex is capable of photo-oxidizing guanosine monophosphate, GMP and guanine sites in both duplex and quadruplex DNA. We also find the complex is spontaneously taken up by live melanoma cells, where it localizes in the nucleus and displays potent photo-toxicity, indicating that it is a readily accessible, highly promising, photosensitizer for the treatment of a highly malignant recalcitrant cancer.

## Results and discussion

We initially set out to synthesize the TAP ligand following established literature procedures.^34^,^42^ The diaminoquinoxaline precursor was readily obtained using the route described by Elmes *et al.*^42^ However, in our hands, existing literature procedures for the final synthesis of TAP from this intermediate proved to be unreliable and low yielding. We found that by using isopropanol as the reaction solvent, along with 1.5 equivalents of glyoxal, the production of intractable side-products could be eliminated, yielding 80% of pure TAP after work-up.

Complex **2**^4+^ was synthesized through an analogous route to that used for **1**^4+^ (see ESI† for a reaction scheme and experimental details).^19^ However, in coupling the two {Ru(TAP)_2_} cores to the tpphz bridge, we found it was necessary to use more forcing reaction conditions that for the related phen or bpy complexes. This is most likely due to the more strongly electron-deficient nature of the TAP ligand reducing the donor strength of the chelating N lone pairs. A microwave-assisted synthesis adapted from a previous report,^39^ combined with purification through alumina column chromatography and then ion-exchange chromatography on Sephadex-LH25 yielded analytically pure **2**^4+^ as a chloride salt in 55% yield. For spectroscopic studies, the hexafluorophosphate was also isolated by anion metathesis.

**Fig. 1:**
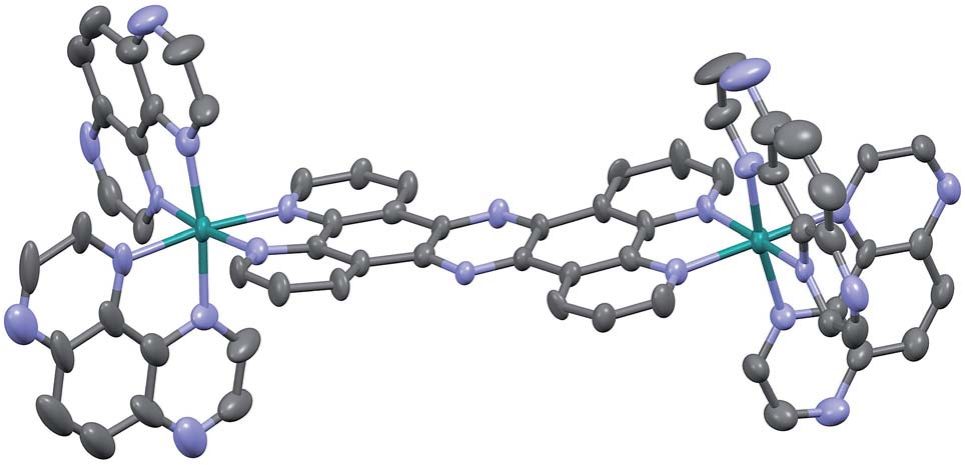
Thermal ellipsoid diagram showing the Δ,Δ cation of the [**2**]Cl_4_ structure. Hydrogen atoms have been omitted for clarity.

Crystals suitable for X-ray crystallography were grown by slow vapour diffusion of acetone into a saturated solution of **2**^4+^ in methanol. Although the quality of the data (*R* = 9.27) prevents a full analysis of bond length and angles, it does confirm connectivities. Interestingly, like the corresponding structure of [{(bpy)_2_Ru}_2_(tpphz)]^4+^,^43^ the resultant unit cell contains two stereoisomers of **2**^4+^ – the Δ,Δ and Λ,Λ forms – with no evidence of the meso Δ,Λ form apparent in the crystal data. One of these cations (the Δ,Δ cation) is shown in Fig. 1.

Two of the chloride counter ions are disordered, and a number of highly disordered water molecules are present within a solvent cavity, which were accounted for in the refinement using a solvent mask.

The distorted octahedral geometry around each ruthenium(ii) core is essentially identical, with a N1–Ru1–N6 angle of 171.8° and the average bond length for the 4 TAP ligands of 2.052 A. The tpphz bridging ligand displays a marked twist – the angle between the planes of the two “bpy” moieties of the tpphz gives a twist of 10.4°. As might be expected, the observed bond angles and lengths are comparable to those of [{(bpy)_2_Ru}_2_(tpphz)]^4+^.

**Fig. 2:**
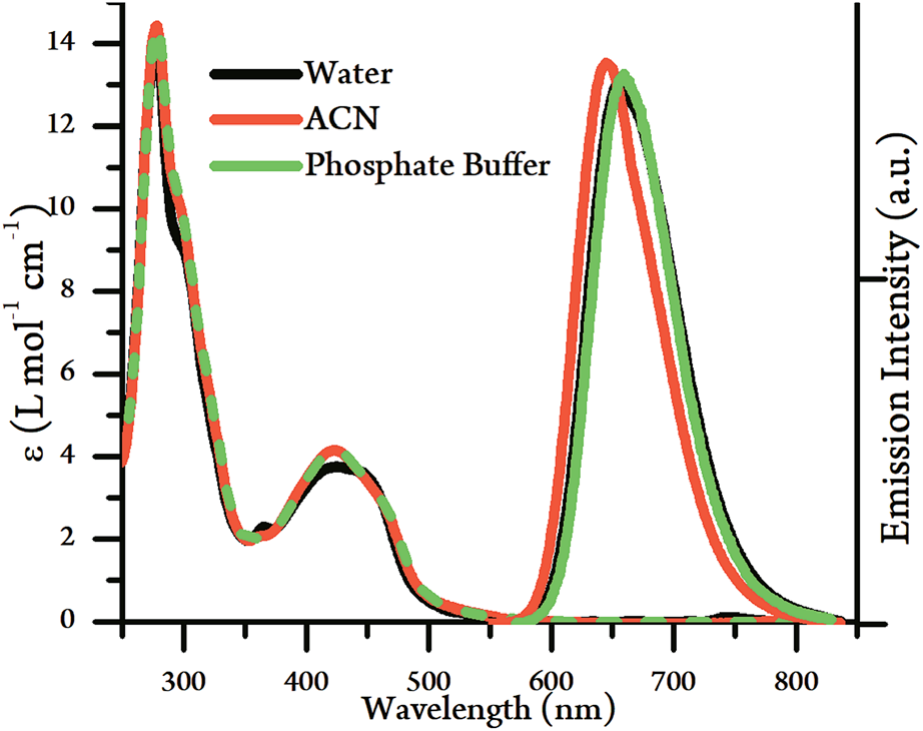
UV/Vis absorption spectra (left) and emission spectra (right) of [**2**]Cl_4_ and [**2**](PF_6_)_4_ in water, acetonitrile and phosphate buffer (10 mM KH_2_PO_4_, 200 mM KCl, pH 7.2, 20 mM EDTA).

The UV/Vis absorption spectra of [**2**](PF_6_)_4_ and [**2**]Cl_4_ were measured in acetonitrile and water respectively. Apart from the expected high-energy intraligand bands, the complex possesses an absorption between 400–500 nm, an energy that is typical for a Ru → L ^1^MLCT transition, see Fig. 2 and Table 1, but it is substantially more intense than the equivalent transition for **1**^4+^. The energy of the ^1^MLCT does not significantly change in acetonitrile compared to aqueous environments, although changes in relative intensities within the band suggests that it is composed of at least two intense, overlapping transitions.

**Table 1:** Photophysical data for complex **2**^4+^

Complex	Absorption		Emission		
	*λ*_max_ (nm)	*ε* (L mol^–1^ cm^–1^)	*λ*_max_ (nm)	Quantum yield	Lifetime (ns)
[**2**]Cl_4_^*a*^	423	41 800	628	0.05 (water)	650 ± 24
	452	33 300		0.04 (phosphate)	575 ± 37
				0.07 (Tris)	723 ± 23
[**2**](PF_6_)_4_^*b*^	423	41 800	613	0.04	550 ± 30
	452	34 800			

^*a*^Measured in H_2_O.

^*b*^Measured in MeCN.

The emission spectra of [**2**](PF_6_)_4_ and [**2**]Cl_4_ were also measured in acetonitrile and water respectively (Fig. 2, Table 1). The complex displays bright emission between 600–750 nm in both solvents, which is in direct contrast to the properties of **1**^4+^,^19^ which is quenched in aqueous media.^44^ The emission wavelength and lifetimes in acetonitrile were very similar to those in aqueous conditions, with only a small blue shift in *λ*_max_ alongside an approximately 20% decrease in emission lifetime and quantum yield. These observations reveal a significant difference in the lowest excited state of **2**^4+^ compared to **1**^4+^, suggesting that – as expected – the emissive state of **2**^4+^ is a TAP-based ^3^MLCT rather than the tpphz-based ^3^MLCT of **1**^4+^. As expected from these observations, emission quantum yield are similar, regardless of solvent environment.

**Fig. 3:**
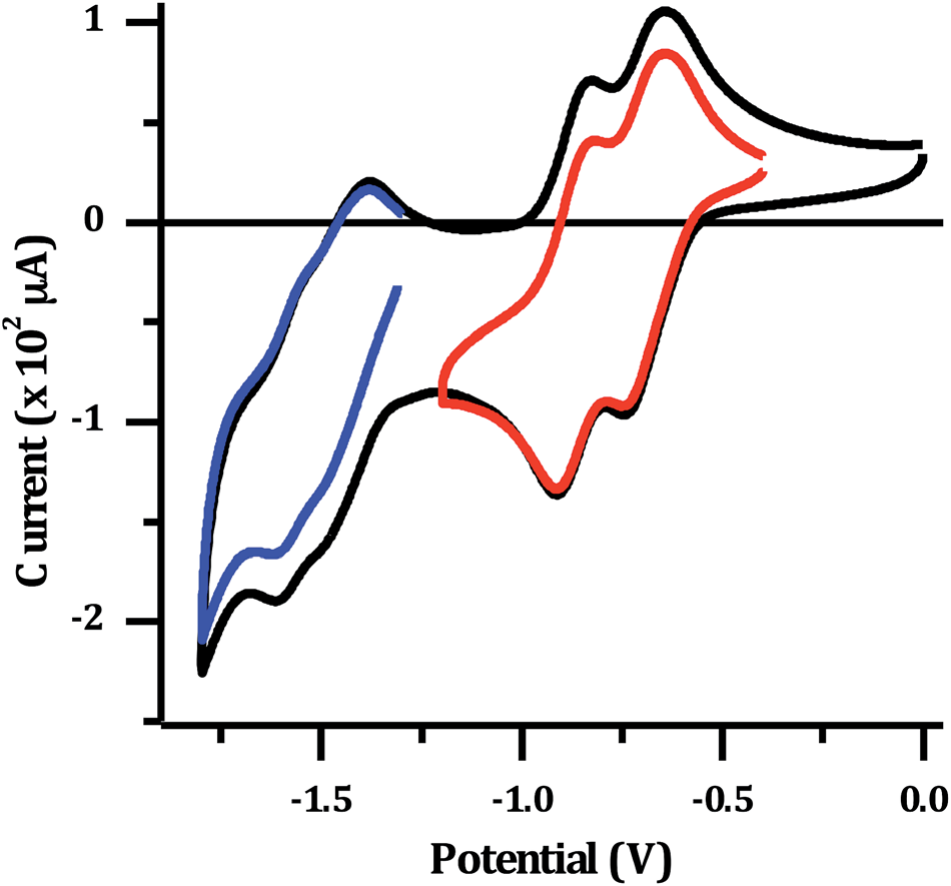
Cyclic voltammogram for 1 mM [**2**](PF_6_)_4_: in dry DMF with 0.1 M N(*n*-Bu)_4_PF_6_ supporting electrolyte. Red and blue lines show isolated sets of reductions.

The electrochemical properties of **2**^4+^ were investigated in dry acetonitrile. The complex displays a single oxidation at approximately 1.8 V *vs.* Ag/AgCl, which appears to be reversible, although it occurs at the edge of the solvent window. This potential is almost identical to that reported for the mononuclear [Ru(TAP)_2_dppz]^2+^ complex^34^ (+1.82 V *vs.* Ag/AgCl), and is therefore assigned to the expected reversible Ru^II/III^ oxidation. The reduction of the complex in acetonitrile results in a large stripping peak on the return sweep. This behaviour has been previously observed for dinuclear complexes with a tpphz bridge^44^ and suggests the reduced form of the complex displays low solubility in MeCN. Therefore, the reduction potentials were also measured in dry DMF (Fig. 3).

Several reduction waves are observed in these conditions. Two distinct reversible reductions occur at –0.70 V and –0.86 V, with further poorly resolved couples occurring between –1.4 to –1.8 V. Given that the emissive state of [**2**]Cl_4_ is not quenched in water, which implies a TAP-based lowest excited state, the reduction at –0.70 V is assigned to the first reduction of the TAP ligand, which is similarly observed in the mononuclear Ru–TAP analogues.^33^,^45^ A second reduction process at –0.86 V is also consistent with the reduction of a TAP ligand coordinated to the second Ru centre. Through comparison to related complexes, the overlapping redox processes between –1.4 V to –1.8 V are assigned to a combination of reductions of the tpphz bridging unit, and further reductions of TAP ligands.

To elucidate our results further a series of density functional calculations using Gaussian 09 (ref. 46) was performed employing methods and procedures outlined in the ESI.† Calculations were performed on **1**^4+^ in acetonitrile and on **2**^4+^ in both acetonitrile and water. In these calculations, we included solvent using a continuum model for MeCN. However, from previous work,^47^ it is clear that some explicit water molecules are needed to describe the electronic structure of these compounds in water correctly, especially since it is known that hydrogen bonding plays an important role in the excited states dynamics of complexes such as **2**^4+^.

**Fig. 4:**
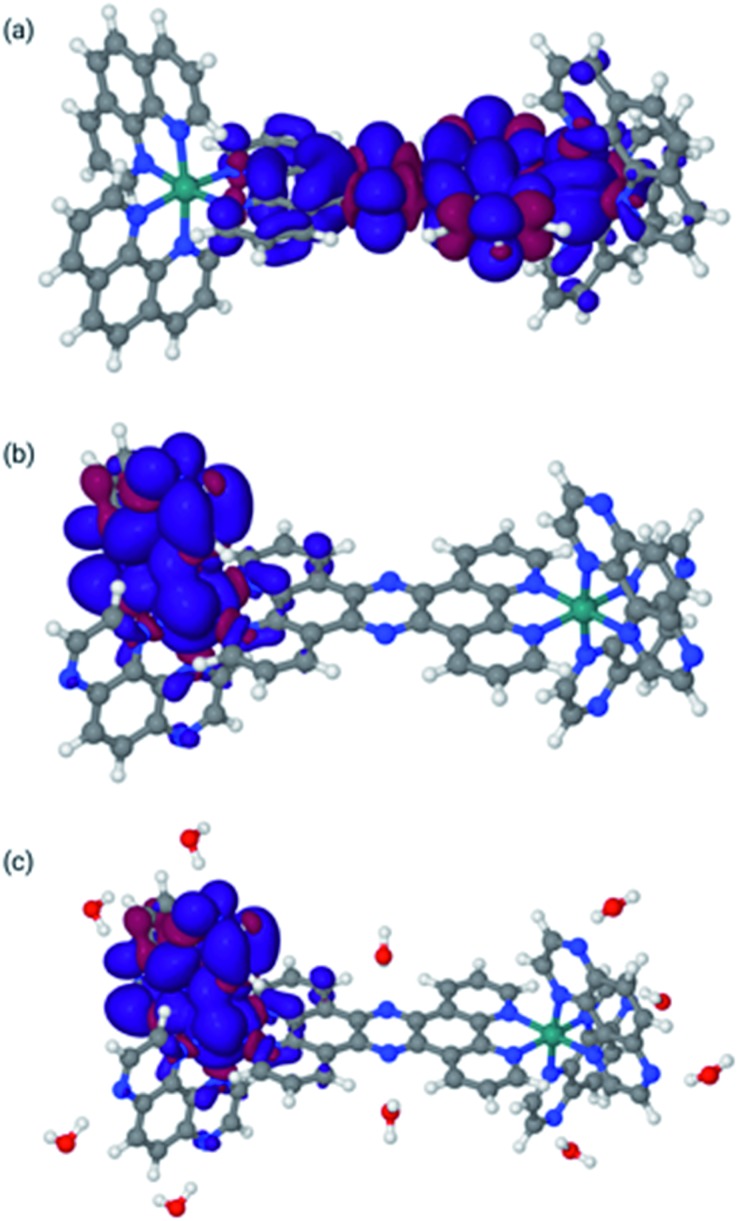
Lowest triplet structures for **1**^4+^ in MeCN [panel (a)], **2**^4+^ in MeCN [panel (b)], and **2**^4+^ in water [panel (c)]. Blue indicates α spin, whereas red indicates β spin.

The final optimized structures for the triplet states are given in Fig. 4, with the spin density super-imposed on the final structure. In these calculations, the singlet (ground-state) structures are indistinguishable from the triplet structures and can be found in the ESI.†

The spin densities and geometries in Fig. 4 show very clearly that, although replacing phen with TAP has little structural effect, the substitution leads to significant electronic changes. In particular, the spin density clearly shows that for **2**^4+^ (irrespective of the solvent) the triplet excited state has its density on one of the TAP ligands, whereas for **1**^4+^ it is situated on the tpphz, confirming our experimental observations (*vide supra*). Close inspection of the LUMO and LUMO+1 orbitals shows that these are part of a (near-) degenerate pair, so that small geometric distortions may alter the specific TAP on which the excited state localizes.

In the calculations associated with Fig. 4c, water molecules were placed in hydrogen-bonding positions around **2**^4+^. These interactions will be associated with many orientations of very similar energies. However, an exhaustive search on this issue is outside the scope of this paper and will most likely lead to very similar results. Nevertheless, it is interesting to note that two water molecules bonding to the central tpphz moiety, which were originally placed in a hydrogen-bonding position, rotated and are in a hydrogen-accepting orientation in the final structure. This observation suggests that the nitrogen atoms of the tpphz bridge are not accessible to hydrogen-bonding, indicating that some of the deactivation pathways available to similar complexes incorporating the Ru^II^(dppz) unit cannot be accessed by **2**^4+^.

Using the singlet geometries, UV-VIS spectra were simulated through TD-DFT calculations, which show qualitative agreement with the experimental absorption spectrum (see ESI Fig. S-9†). However, our calculations also show that there are many transitions underlying absorption bands in the visible region. To investigate whether the nature of the transitions is affected by changing the solvent from MeCN to water, a wave function analysis^48^–^50^ was performed on the highest fifty states in the TD-DFT calculation. The strongest five transitions from this analysis are also represented graphically in the ESI (see Fig. S-10).†

The wave function analysis indicated that transitions in both solvents occur at similar energies, resulting in what appear to be the near-identical experimental UV-VIS spectra shown in Fig. 2. However, it is also clear that the nature of each of these transitions is very different.

Again, the analysis yields results that are consistent with the experimental data. For example, in MeCN and water, transitions with similar energy – at 425.8 and 421.0 nm, respectively – are predicted. Yet, whilst this transition in MeCN is assigned to a MLCT from both Ru atoms onto the tpphz bridge, in water the transition is predicted to be a MLCT from the Ru atoms onto the TAP units. Interestingly, some of the transitions are completely different in their nature. For example, transitions at 389.1 nm and 386.5 nm in MeCN are again MLCT transitions onto tpphz, but in water, where these transitions occur at 385.0 nm and 381.0 nm, respectively, they are inter-ligand transitions from tpphz onto TAP for the latter transition and a mix of inter-ligand transitions and an MLCT transition for the former transition.

The calculated triplet geometries and corresponding singlet states were also used to predict the emission wavelength assuming no geometrical relaxation. In MeCN, the emission maximum of **2**^4+^ was calculated to be at 647 nm and in water the corresponding wavelength was predicted to be 677 nm. The corresponding emission wavelengths for the 0–0 transitions (which includes geometric relaxation) were at 607 and 637 nm, respectively. In both cases, there is a correlation with both the ordering and approximate energies of experimentally measured spectra as shown in Fig. 2.

**Fig. 5:**
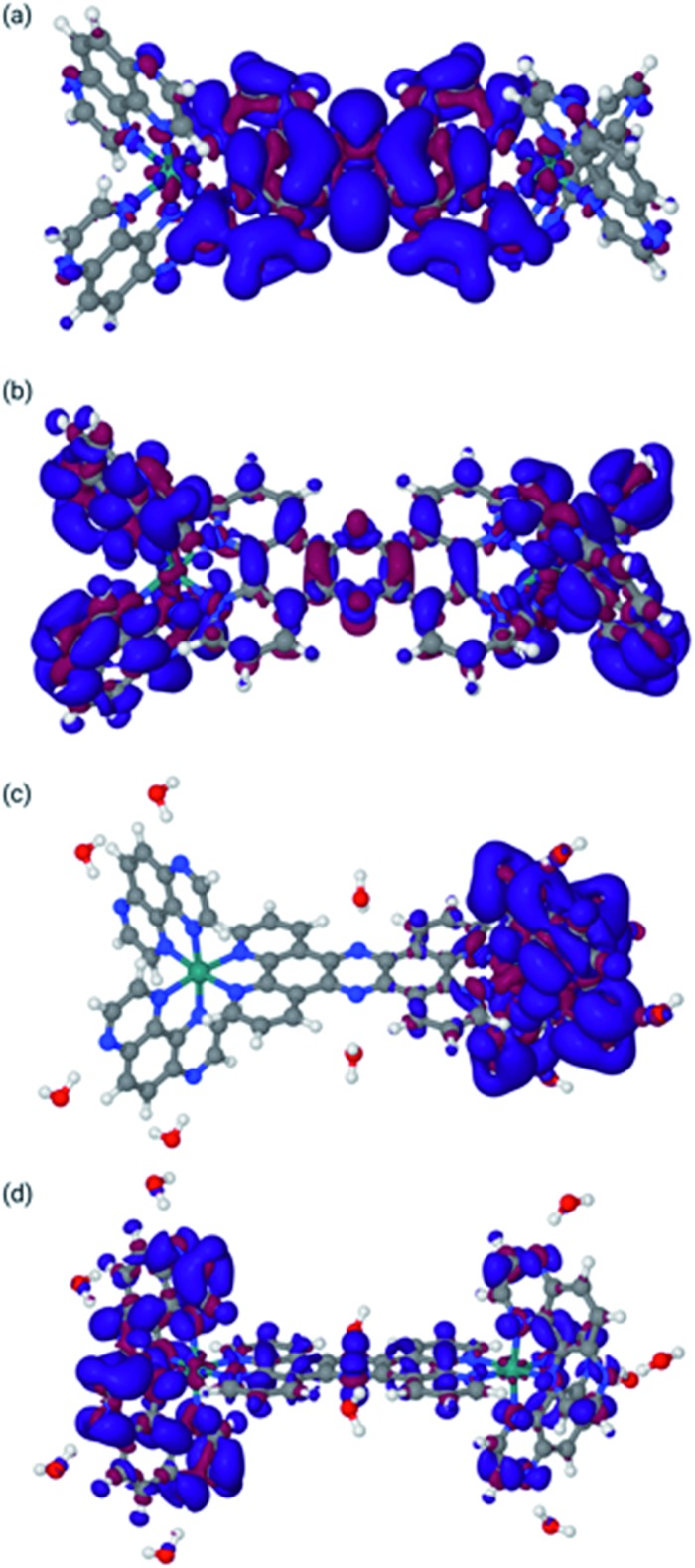
Difference density maps for **2**^2+^ in acetonitrile [panels (a) and (b)] and water [panels (c) and (d)]. Panel (a): Differential density for reduction from **2**^4+^ to **2**^3+^ in MeCN. Panel (b): Differential density for reduction from **2**^3+^ to **2**^2+^ in MeCN. Panel (c): Differential density for reduction from **2**^4+^ to **2**^3+^ in water. Panel (d): Differential density for reduction from **2**^3+^ to **2**^2+^ in water. Blue indicates increase of electron density, whereas red indicates loss of electron density (see ESI for the methodology used).

Finally, the behaviour upon reduction of these complexes was investigated computationally. The differences in electron density for the first and second reduction of **2**^4+^ in both MeCN and water were calculated and are shown in Fig. 5.

Concentrating on the panels (c) and (d) first, the calculations are in agreement with the experimental data. They clearly indicate that both reductions are mainly centred on the TAP ligands. It is also clear that the water molecules do not play a direct role in the reduction, in that there is no density difference associated with them. However, water molecules do respond to the reduction by adapting their orientation with respect to **2**^4+^ as is clear from panel (d), where the water molecules bonded to the tpphz ligand in **2**^3+^ are polarized towards the reduced TAP ligand.

The situation in MeCN as shown in panels (a) and (b) of Fig. 5 is somewhat different. Here, the first reduction is of the tpphz ligand, whereas the TAP ligands are reduced in the second reduction. This is in line with the virtual orbitals of **2**^4+^ in MeCN, but is different from what happens upon photo-excitation, where the triplet state is clearly located on the TAP ligand. Clearly, these virtual orbitals are close enough to facilitate this re-arrangement upon reorganization of electron density. It should be pointed out that this result is slightly different to the observed electrochemistry for [**2**](PF_6_)_4_; however, the experimental data were collected in DMF and it the calculations indicate that the electronic structure of this system is solvent dependent.

Given the interesting redox properties of **2**^4+^ revealed by the experimental data and computational studies, the photo-oxidizing properties of the complex were investigated.

**Fig. 6:**
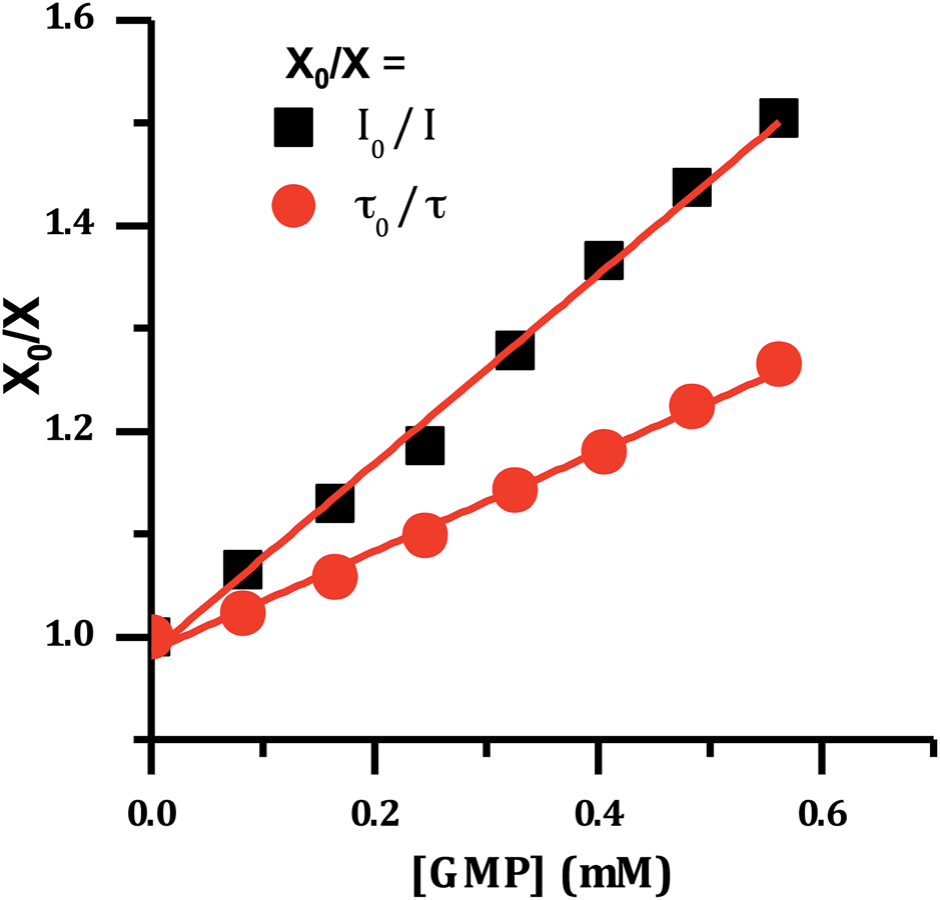
Stern–Volmer plot for the emission of [**2**]Cl_4_ quenched by the addition of guanosine monophosphate, showing change in lifetime and emission intensity.

It has previously been demonstrated that [Ru(TAP)_2_(dppz)]^2+^ and related complexes photo-oxidize DNA through electron transfer from guanine sites to the photo-excited complex, quenching its emissive state.^33^–^36^,^51^ Therefore the effect of increasing concentrations of nucleotide monophosphates on the emission spectrum of **2**^4+^ was investigated and as expected these revealed that only addition of GMP led to emission quenching (Fig. 6).

The difference between *I*_0_/*I* and *τ*_0_/*τ* as a function of quencher concentration indicate that emission changes are caused by both dynamic and static quenching processes. Such effects often occur through stacking interactions between nucleotides and luminophores,^52^,^53^ therefore this possibility was investigated using ^1^H-NMR.

Although distinct changes in the chemical shifts for a number of protons on **2**^4+^ were observed on addition of GMP, these were difficult to quantify as they were accompanied by significant signal broadening. However, significant chemical shifts were simultaneously observed for several GMP signals. Notably, the purine proton at the 8-position shifted from *δ* 7.95 to 8.06, and the 1′ sugar proton *δ* 5.60 to 5.75. Fitting these latter changes to a simple 1 : 1 guest–host binding model gave a *K*_a_ of 8.3 × 10^2^ M^–1^. It is known that large anions can interact with dinuclear complexes by binding into the cleft defined by ancillary and bridging ligands. Indeed, this has formed the basis of chromatographic separation of stereoisomers of such complexes.^54^–^56^ Thus, the observed NMR shifts indicate that GMP interacts with **2**^4+^ in this manner.

The emission quenching data were then fitted using the relationship:^57^
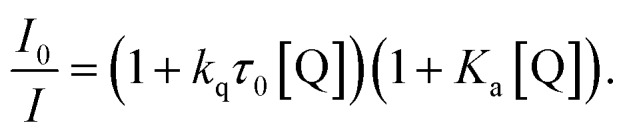
Where, *k*_q_ is the quenching constant, *τ*_0_ is the emission lifetime of **2**^4+^ without a quencher, [Q] is quencher concentration and *K*_a_ the association constant. This analysis gives a quenching constant of 1.2 × 10^8^ M^–1^ s^–1^. No significant emission quenching was observed on addition of A, T and C monophosphates confirming that – as expected from previous studies – quenching was exclusive to GMP.

For the interaction of **2**^4+^ with both duplex and quadruplex DNA was the investigated through absorption and luminescence titrations. To aid comparisons, all titrations in these studies (and the ESI†) were carried out in high ionic strength phosphate buffer, which is consistent with quadruplex folding (10 mM KH_2_PO_4_, 200 mM KCl, 0.1 M EDTA, pH 7.0). Absorption measurements were taken in parallel, both to assess any changes in the absorption spectrum itself and to correct the emission intensities.

**Fig. 7:**
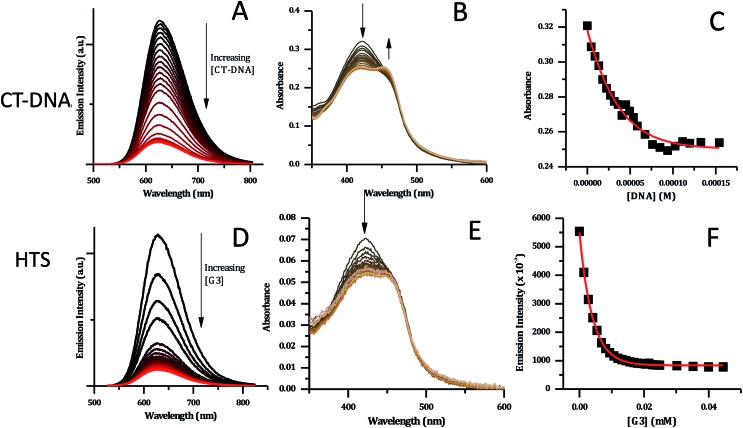
CT-DNA and HTS titration data for [**2**]Cl_4_ (5 μM concentration, in phosphate buffer). (A) Changes in emission spectra upon addition of CT-DNA. (B) Changes in absorption spectra with CT-DNA. (C) Binding curve for the data obtained with CT-DNA based on absorption data – red line shows fit to model developed by Srishailam *et al.*^7^ (D) Changes in emission spectra upon addition of HTS. (E) Changes in absorption spectra of [**2**]Cl_4_ upon addition of HTS. (F) Binding curve for [**2**]Cl_4_ with HTS based on emission data – red curve shows fitting to a one-set-of-sites binding + non-specific interaction mode.

As expected from the GMP experiments and similar studies on [Ru(TAP)_2_(dppz)]^2+^, complex **2**^4+^ shows emission quenching on binding to CT-DNA – Fig. 7A.^34^,^36^ Again, this observation is consistent with a Ru^II^ → TAP-based ^3^MLCT excited state and indicates the expected photoredox quenching by G-sites within the duplex. This is confirmed by the fact that addition of poly(A)–poly(T) causes a slight increase in emission (see ESI†).

DNA-induced changes were also observed in the absorption spectrum of **2**^4+^ – Fig. 7B. The MLCT transition at 420 nm shows a significant decrease in intensity throughout the titration. Approaching the end point of the titration, a new shoulder at 460 nm appears to grow in, suggesting that binding to CT-DNA may affect the energy of excited states. This is consistent with the previously described DFT calculations indicating that order of the close lying excited states of the complex is sensitive to its environment.

Attempts to fit the titration data to the well-known McGhee–von Hippel model^58^ were unsuccessful; it is possible that this is due to the presence of multiple binding modes. However, the absorbance data fitted well to the simpler binding model described by Srishailam *et al.*,^7^Fig. 7C, resulting in an estimated *K*_b_ of 2 × 10^6^ M^–1^, which is of the same order of magnitude as isostructural **1**^4+^,^19^ implying the addition of the heteroaromatic nitrogens to the ligands does not significantly affect binding to duplex DNA.

Previous work within the Thomas group has shown that **1**^4+^ binds to quadruplex-folded human telomere sequence (HTS) with high affinity.^19^,^20^,^23^ Therefore, analogous experiments were carried out with **2**^4+^ using the same experimental conditions. HTS-induced changes in emission spectra are similar to those observed on CT-DNA addition; as HTS is G-rich quenching by G-sites is again expected – Fig. 7D. However, a closer inspection reveals differences in the absorption changes induced by binding to HTS and CT-DNA – Fig. 7E. In particular, whilst an initial decrease in absorption of the 423 nm band is observed on addition of HTS, the subsequent grow-in of the band at 460 nm observed in duplex binding does not occur, suggesting that binding to quadruplex does not affect the energy of excited states of **2**^4+^ in the same way as duplex binding.

The emission titration data was also best fitted to a one-set-of-sites binding + non-specific interaction model – Fig. 7F. This yielded an estimated binding constant of 5.1 × 10^5^ M^–1^, which is an order of magnitude lower than the binding constant to the duplex and twenty-fold lower than the binding of **1**^4+^ to HTS.

The difference in quadruplex binding affinities between the two complexes may be explained by a consideration of our previously reported NMR-based HTS/**1**^4+^ structure, which shows close contacts between the ancillary ligands of **1**^4+^ and the diagonal loops of the quadruplex structure. It seems the lone pairs of the aromatic nitrogen of the ancillary TAP ligands destabilizes this interaction through unfavourable interactions with similar basic sites within the diagonal loop.

**Fig. 8:**
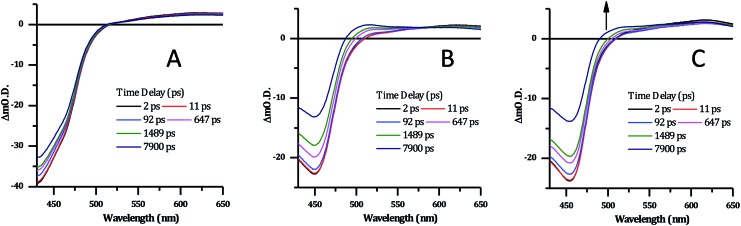
Selected transient absorption data for [**2**]Cl_4_ following ∼40 fs, 400 nm excitation. (A) In phosphate buffer. (B) In phosphate buffer with 1 mM CT-DNA. (C) In phosphate buffer with 1 mM HTS.

**Fig. 9:**
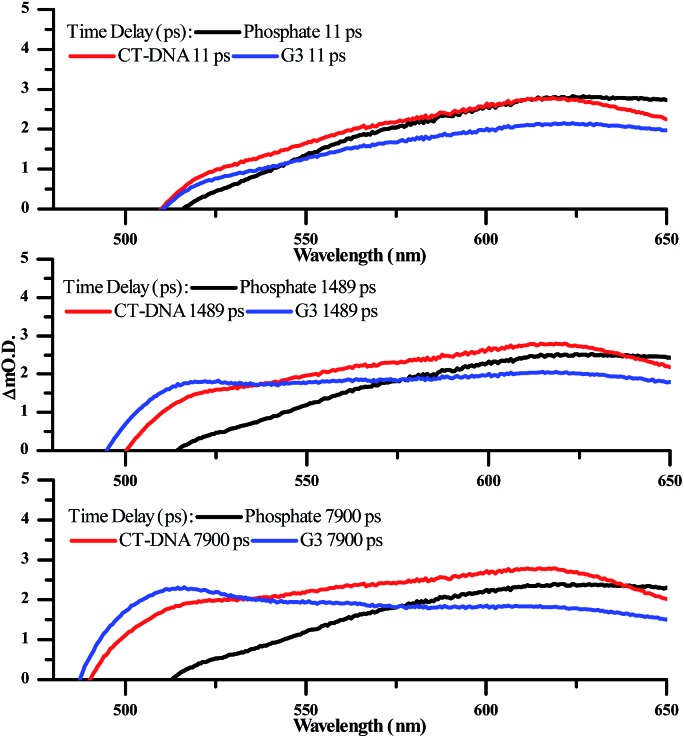
Selected transient absorption spectra for [**2**]Cl_4_ in phosphate buffer with and without CT-DNA and HTS, showing the growth of the transient band at ∼515 nm.

To further investigate the changes in the excited state of DNA bound **2**^4+^ transient absorption experiments were carried out. Absorption transients were measured in phosphate buffer at a concentration of 0.1 mM complex – Fig. 8 and 9. Global lifetime analysis of the TA data was carried out using Glotaran 1.5.1,^59^ with kinetics of selected processes fitted using Origin 8.0.

Previous studies on Ru complexes of dppz and tpphz have revealed that intense transient bands in the 500–800 nm region are produced by charge transfer to the phenazine moiety of the ligands.^36^,^60^–^63^ However, the computational and steady-state optical studies described above suggested that the lowest excited state for complex **2**^4+^ is a Ru → TAP MLCT. These conclusions are further supported by the transient spectra for [**2**]Cl_4_ which show that the strong ground-state bleach of the MLCT absorption bands between 400–500 nm is accompanied by the growth of a weak transient between 500–650 nm, changes which occur immediately following the pump pulse (within 1 ps) – Fig. 8A. These observations are consistent with previous studies on [Ru(TAP)_2_(dppz)]^2+^ in solution – which also shows a weak, broad, largely featureless transient absorption between 500–650 nm – and confirm that **2**^4+^ displays a TAP centred excited state.^34^,^64^

There are no significant changes in spectral shape throughout the time-window of the experiment (7900 ps). Global fitting of the spectra using 4-exponential decay parameters gave the most reliable fitting of the data. The two fastest components of under ∼1 ps, are followed by simultaneous decay of the entire spectrum, with components of 355 ps and ∼100 ns. The slow component is assigned to the emissive excited state observed above. The 355 ps component is likely to be due to conversion of a multitude of initially-formed excited states to the long-lived Ru → TAP ^3^MLCT state, as lifetimes of a similar order of magnitude have been previously observed for [Ru(TAP)_2_(dppz)]^2+^ and related complexes.

Significantly, in both the CT-DNA and HTS experiments, but not a distinctive signal at approximately 515 nm emerges – Fig. 9. A very similar transient is observed when DNA is added to [Ru(TAP)_2_(dppz)]^2+^ and this was assigned to the generation of the guanine radical cation as a photo-oxidization product.^35^,^36^,^64^,^65^ Interestingly, this feature is appreciably more prominent on addition of HTS, which as a sequence possesses a higher density of G-residues compared to CT-DNA, and is therefore more likely to undergo photo-oxidation on exposure to light.

The excited state dynamics of **2**^4+^ in the presence of CT-DNA and HTS are complex, with global lifetime analysis suggesting a minimum of four distinct excited state processes. The lifetimes of these states are of similar magnitude in the presence of both CT-DNA and HTS (Table 2).

**Table 2:** Lifetimes obtained from global lifetime analysis of transient absorption data

	Lifetimes (ps)	
	CT-DNA	HTS
*τ*_1_	3.6 ± 0.4	2.9 ± 0.2
*τ*_2_	275 ± 12	330 ± 17
*τ*_3_	2900 ± 150	2930 ± 130
*τ*_4_	>30 000	>30 000

The decay-associated spectra (see ESI‡) for each excited state process feature a grow-in signal at ∼510 nm, associated with the photooxidation of guanine. This implies that this photo-oxidation process can occur in parallel from multiple excited states. Unfortunately, there is insufficient detail in the transient absorption data to accurately model these processes. However, by isolating and fitting the feature at 510 nm using a 3-exponential model, the major lifetime component of this growth is 1808 ± 100 ps for CT-DNA and 1592 ± 90 ps for HTS. The faster kinetics for HTS is consistent with the anticipated intimate contact between **2**^4+^ and guanine tetrads of the four-stranded structure.

The intricacies of the dynamics reflect the complexity of binding modes and the numerous potential photochemical pathways following photoexcitation. Assuming the lowest triplet excited state of the complex is a Ru → TAP ^3^MLCT state (as confirmed by the calculations), then subsequent simultaneous photooxidation of guanine, radiative and non-radiative decay, as well as c of the oxidised guanine residues will all contribute to the spectral evolution. Significantly more detailed analyses, such as time-resolved infrared spectroscopy, will be required to fully dissect the subtleties, which will form the basis of a future report. Nevertheless – although it is clear that **2**^4+^ preferentially binds to duplex over quadruplex DNA – the cell-free studies described above confirm that this complex does participate in photo-induced redox reactions with G-sites, whether they are found in duplex or quadruplex structures.

With these promising results in mind, the live cell uptake and localization of **2**^4+^ was investigated. For reasons of therapeutic need, malignant human melanoma cells were specifically chosen for these studies.

Melanoma is one of the most aggressive and therapeutically resistant cancers. If diagnosed and treated in its early stages it has an 80% 10 year remission rate. However, if it spreads to the lymph nodes this figure drops to only 10%.^66^ In 2017 malignant melanoma was responsible for 72% of skin cancer deaths in the USA and its incidence continues to rise.^67^ One of the difficulties in treating melanoma is that it displays a range of therapeutic resistance mechanisms,^68^ so alternative treatment regimes are being actively sought.^69^ In this context, it has been suggested that PDT could provide a novel therapeutic modality for this cancer.^66^,^70^ We chose to investigate the effect of **2**^4+^ on human C8161 melanoma cells as this line is known to be highly invasive and spontaneously metastatic,^71^ and thus it represents a relevant and therapeutically challenging treatment target.

**Fig. 10:**
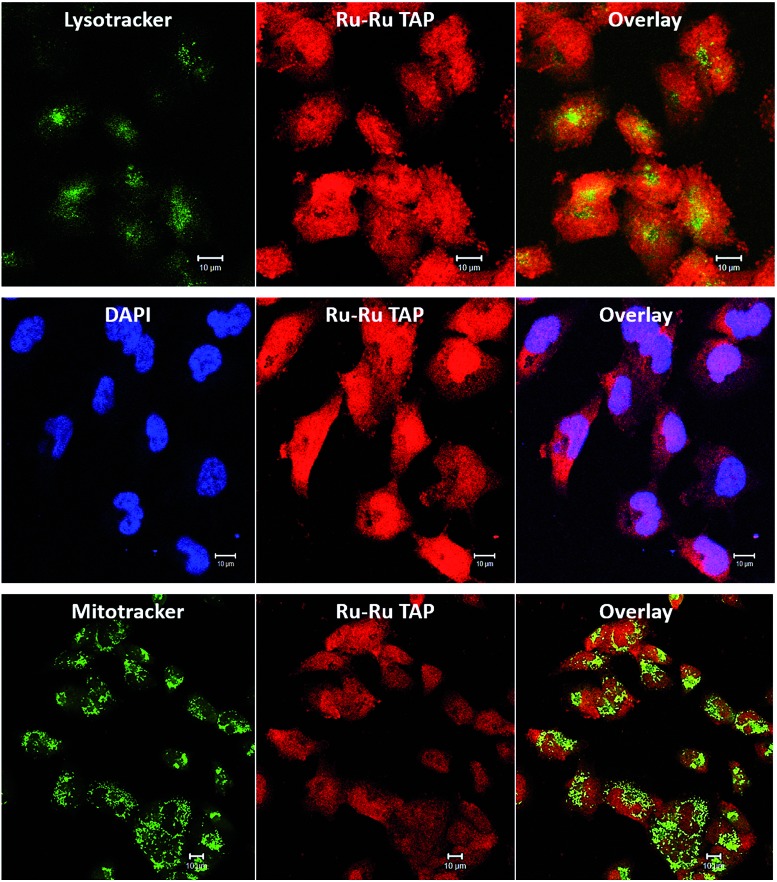
Intracellular localization and uptake of [**2**]Cl_4_ in human C8161 melanoma cells – labelled Ru–Ru TAP in diagrams. Co-staining with cytoplasm stains LysoTracker Green (top) and MitoTracker Green (bottom) show low colocalisation with [**2**]Cl_4_ (Pearson coefficient = 0.06 ± 0.01, and 0.12 ± 0.04, respectively, SB 10 μm). Co-staining with nuclear stain DAPI (centre) reveals a significantly higher co-localisation (Pearson coefficient = 0.51 ± 0.19, scale bar = 10 μm).

Encouragingly, it was found that the complex was spontaneously taken up by live C8161 cells, and it produced bright intracellular emission. Furthermore, co-staining using **2**^4+^ (100 μM, 24 hours) and the standard nuclear stain DAPI, reveals DAPI co-localizes with the new complex, displaying a Pearson coefficient of 0.51. These observations confirm that – like its close analogue **1**^4+^ – complex **2**^4+^ is cell and nuclear membrane permeant. However, unlike **1**^4+^, treatment with **2**^4+^ also leads to bright emission from the cytoplasm of the melanoma cells, therefore analogous co-staining experiments with commercial stains were carried out, Fig. 10.

These studies revealed some correlation between the emission of the complex and that of the MitoTracker Green and LysoTracker Green labels. However, calculated Pearson coefficients of 0.12 and 0.06, respectively, indicate localization of **2**^4+^ within these organelles is significantly lower than within nuclei. This is consistent with recent super-resolution and TEM studies^72^ that revealed that, while **1**^4+^ is only brightly emissive from the nucleus, it does exhibit significant mitochondrial localization. The difference in the intracellular luminescence of the complexes can be attributed to the fact that whilst **1**^4+^ displays off/on DNA light-switch emission, **2**^4+^ is emissive, unless quenched by photo-redox processes.

To facilitate a more detailed understanding of cellular uptake, the concentration- and time-dependent uptake of **2**^4+^ was explored – see ESI.† It was found that intracellular emission intensity correlates with increased concentrations and exposure times. These studies confirmed that the complex readily diffuses into cells, where initially it predominately labels the nucleus, but then diffuses more generally throughout the cell.

**Fig. 11:**
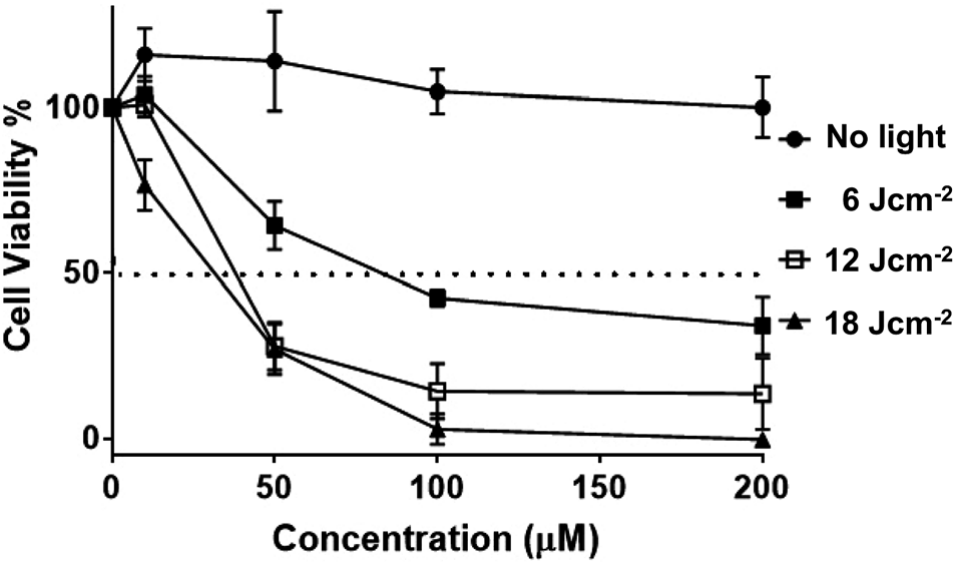
Phototoxicity of **2**^4+^ on melanoma. Human melanoma cells (C8161) were treated at a series concentration of **2**^4+^ (0, 10, 50, 100, 200 μM). Photo-toxicity was assessed using a 405 ± 20 nm wavelength light from a ThorLab LED lamp (M405LP1, power output 1500 mA) on exposure to fluences of 6 J cm^–2^, 12 J cm^–2^ and 18 J cm^–2^, respectively. No cell death was observed with an increase in **2**^4+^ compound concentration. However, cell viability decreased significantly after exposure to 405 ± 20 nm light with pre-treated melanoma cells (*e.g.* 100 μM with 1 hour (■) light activation causes >50% reduction in cell viability). LD_50_ value calculated (for each line interpolate sigmoidal) using Prism software. The error bars denotes SEM (*n* = 3).

Having established uptake by live melanoma cells and specific sub-cellular localizations, the cytotoxicity and phototoxicity of **2**^4+^ was then investigated. Cell viabilities after exposure to different concentrations of the complex in the dark after 24 hours were quantified using the AlamarBlue method – Fig. 11.^58^–^60^

These studies indicated that the initial seeding density of 1 × 10^5^ cells per well does not change on treatment with the complex. Indeed, at all concentrations, no statistical difference in cell viability was observed compared to cells of non-treated controls, suggesting the complex displays negligible cytotoxicity in dark conditions (see ESI†). Experiments were then carried out in which melanoma cells treated with various concentrations of complex were irradiated into the MLCT band using a 405 ± 20 nm LED laser. Light treated cells were then incubated for 3 hours, and a change in cell viability measured, and plotted against various concentrations of **2**^4+^.

Irradiation at low fluences, with a maximum of only 18 J cm^–2^, showed a dose–response hyperbolic curve, revealing a radical drop to effectively zero cell-viability at exposure to 100 μM of **2**^4+^, compared to non-irradiated cells. A similar pattern of concentration-dependent cell death was also seen on exposure to a broad-spectrum blue light, a regime commonly employed by dermatologists in the treatment of topical skin conditions such as acne.^73^ We note that this treatment required longer time exposures, but this is probably due to differences in power output. Nonetheless, the new complex is therapeutically active at fluences that are around an order of magnitude lower than those commonly employed for commercial sensitizers in the treatment of cancer, including basal cell skin cancer.^74^–^77^

## Conclusions

Complex **2**^4+^ displays similar photophysical properties to previously reported systems based on the (TAP)_2_Ru^II^ moiety in that it displays a reactive Ru → TAP MLCT excited state that photo-oxidizes guanosine moieties. Steady-state and time-resolved studies indicate that the complex participates in similar process when bound to both duplex and G-rich, quadruplex DNA. Crucially, this new compound is spontaneously taken up by live melanoma cells, largely localizing in nuclei. The complex shows no toxicity under dark condition, even at high concentrations (*e.g.* 200 μM). However, once sensitized with light, it becomes highly toxic to human melanoma cells, making it an efficient and promising lead as a photosensitizer for PDT.^1^

Complex **2**^4+^ binds to a quadruplex structure with affinities that are around an order of a magnitude weaker than those displayed for duplex binding. Given that conventional duplex DNA vastly outnumber putative quadruplex sequences within the genome,^78^–^81^ it seems likely that the photo-cytotoxic effects of this complex is largely due to damage to duplex sequences. However, damage to quadruplex structures could still be disproportionately large. Previous studies using photo-redox active metal complexes have demonstrated that G-rich sequences, particularly runs of neighbouring G-sites, are susceptible to redox damage,^51^,^82^ even when distal to the metal complex binding site.^83^–^86^ Furthermore, experimental evidence has accrued that quadruplexes can behave as oxidative traps for long-range charge-transfer.^87^,^88^ For this reason it has been suggested that telomeres may be oxidation hot-spots or traps for oxidative damage to genomic content.^88^,^89^ *Inter alia*, future studies involving **2**^4+^ will assess the likelihood of this hypothesis. Inspired by this lead, the synthesis of related photo-redox active structures that display higher selectively for quadruplex over duplex DNA would provide a novel form of telomere targeted PDT; work on targeting such structures is currently underway.

## Conflicts of interest

There is no conflict of interest to declare.

## Supplementary Material

Supplementary information

Crystal structure data
