# Correction: A dinuclear ruthenium(ii) phototherapeutic that targets duplex and quadruplex DNA

**DOI:** 10.1039/d0sc90032j

**Published:** 2020-02-13

**Authors:** Stuart A. Archer, Ahtasham Raza, Fabian Dröge, Craig Robertson, Alexander J. Auty, Dimitri Chekulaev, Julia A. Weinstein, Theo Keane, Anthony J. H. M. Meijer, John W. Haycock, Sheila MacNeil, James A. Thomas

**Affiliations:** Department of Chemistry, University of Sheffield Brook Hill Sheffield S3 7HF UK james.thomas@sheffield.ac.uk +44 (0)114 222 9325; Materials Science & Engineering, University of Sheffield Mappin St Sheffield S1 3JD UK j.w.haycock@sheffield.ac.uk s.macneil@sheffield.ac.uk

## Abstract

Correction for ‘A dinuclear ruthenium(ii) phototherapeutic that targets duplex and quadruplex DNA’ by Stuart A. Archer *et al.*, *Chem. Sci.*, 2019, **10**, 3502–3513.

The authors regret that incorrect details were given for ref. 41 in the original article. The correct details for ref. 41 are given below as ref. 1.

The Royal Society of Chemistry apologises for these errors and any consequent inconvenience to authors and readers.
