# Supplementary material for: A dinuclear ruthenium(ii) phototherapeutic that targets duplex and quadruplex DNA
Source: Chem Sci. 2019 Feb 18;10(12):3502–13. doi: 10.1039/c8sc05084h (PMC6430095; doi:10.1039/c8sc05084h)
Supplement: Supplementary file 1 [file SC-010-C8SC05084H-s001.pdf]

Electronic Supplementary Material (ESI) for Chemical Science.
This journal is © The Royal Society of Chemistry 2019

A dinuclear ruthenium(II) phototherapeutic that targets duplex and quadruplex DNA Supplementary information

Stuart A. Archer,[1] Ahtasham Raza,[1] Fabian M. Droege,[1] Craig Robertson,[1] Alexander J. Auty,[1] Dimitry M. Chekaluev,[1] Julia A. Weinstein,[1] Theo Keane,[1] Anthony J. H. M. Meijer,[1] John W. Haycock,[2,*] Sheila MacNeil,[2,†] and James A. Thomas[1,‡]

[1] *Department of Chemistry, University of Sheffield, Sheffield, S3 7HF, United Kingdom*
[2] *Department of Materials Science and Engineering, University of Sheffield, Sheffield S10 2TN, United Kingdom*

(Dated: November 12, 2018)

* j.w.haycock@sheffield.ac.uk
† s.macneil@sheffield.ac.uk
‡ james.thomas@sheffield.ac.uk

CONTENTS

S1. Additional Experimental Figures S4
- S1.1. Selected NMR Spectra S4
- S1.2. Additional Computational Figures S11

S2. Materials and Methods S13
- S2.1. Synthesis S13
  - S2.1.1. Synthesis of 4-nitroquinoxaline S13
  - S2.1.2. Synthesis of 4-amino-5-nitroquinoxaline S13
  - S2.1.3. Synthesis of 4,5-diaminoquinoxaline S14
  - S2.1.4. Synthesis of 1,4,5,8-tetraazaphenanthrene S14
  - S2.1.5. Synthesis of tetrapyridophenazine S15
  - S2.1.6. Synthesis of Ruthenium (II) 1,5-cycloocadiene dichloride S15
  - S2.1.7. Synthesis of Ruthenium(II) bis(1,4,5,8-tetraazaphenanthrene)dichloride S15
  - S2.1.8. Synthesis of bisRuthenium (II) bis(1,4,5,8-tetraazaphenanthrene)tetrapydridophenazine tetrachloride [**2**].Cl_4 S15
- S2.2. Cell Work S16
  - S2.2.1. Cell culture S16
  - S2.2.2. Intercellular localization S16
  - S2.2.3. Cellular uptake S16
  - S2.2.4. Light radiation source S16
  - S2.2.5. Cell metabolic assay (Alamar blue) S16
  - S2.2.6. Statistical Analysis S17

S3. Computational Methods S17

S4. Additional Tables S18

S5. Calculations on $\mathbf{1}^{4+}$ (^{1}A) in MeCN S19
- S5.1. Cartesian Co-ordinates (XYZ format) S19
- S5.2. Frequencies S22

S6. Calculations on $\mathbf{1}^{4+}$ (^{3}A) in MeCN S29
- S6.1. Cartesian Co-ordinates (XYZ format) S29
- S6.2. Frequencies S32

S7. Calculations on $\mathbf{2}^{4+}$ (^{1}A) in MeCN S39
- S7.1. Cartesian Co-ordinates (XYZ format) S39
- S7.2. Frequencies S42

S8. Calculations on $\mathbf{2}^{4+}$ (^{3}A) in MeCN S48
- S8.1. Cartesian Co-ordinates (XYZ format) S48
- S8.2. Frequencies S51

S9. Calculations on $\mathbf{2}^{4+}$ (^{1}A) at ^{3}A structure in MeCN S57
- S9.1. Cartesian Co-ordinates (XYZ format) S57

S10. Calculations on $\mathbf{2}^{4+}$ (^{1}A) in water (explicit + PCM) S60
- S10.1. Cartesian Co-ordinates (XYZ format) S60
- S10.2. Frequencies S64

S11. Calculations on $\mathbf{2}^{4+}$ (^{3}A) in water (explicit + PCM) S72
- S11.1. Cartesian Co-ordinates (XYZ format) S72
- S11.2. Frequencies S76

S12. Calculations on $\mathbf{2}^{4+}$ (^{1}A) at ^{3}A structure in water (explicit + PCM) (Single Point) S84
- S12.1. Cartesian Co-ordinates (XYZ format) S84

S13. Calculations on $\mathbf{2}^{3+}$ (^{2}A) at $\mathbf{2}^{4+}$ (^{1}A) structure in MeCN (Single Point) S88
S13.1. Cartesian Co-ordinates (XYZ format) S88

S14. Calculations on $\mathbf{2}^{3+}$ (^{2}A) in MeCN S91
S14.1. Cartesian Co-ordinates (XYZ format) S91
S14.2. Frequencies S94

S15. Calculations on $\mathbf{2}^{2+}$ (^{1}A) at $\mathbf{2}^{3+}$ (^{2}A) structure in MeCN (Single Point) S100
S15.1. Cartesian Co-ordinates (XYZ format) S100

S16. Calculations on $\mathbf{2}^{3+}$ (^{2}A) at $\mathbf{2}^{4+}$ (^{1}A) structure in water (explicit + PCM) (Single Point) S103
S16.1. Cartesian Co-ordinates (XYZ format) S103

S17. Calculations on $\mathbf{2}^{3+}$ (^{2}A) in water (explicit + PCM) S107
S17.1. Cartesian Co-ordinates (XYZ format) S107
S17.2. Frequencies S111

S18. Calculations on $\mathbf{2}^{2+}$ (^{1}A) at $\mathbf{2}^{3+}$ (^{2}A) structure in water (explicit + PCM) (Single Point) S119
S18.1. Cartesian Co-ordinates (XYZ format) S119

References S123

S1. ADDITIONAL EXPERIMENTAL FIGURES

S1.1. Selected NMR Spectra

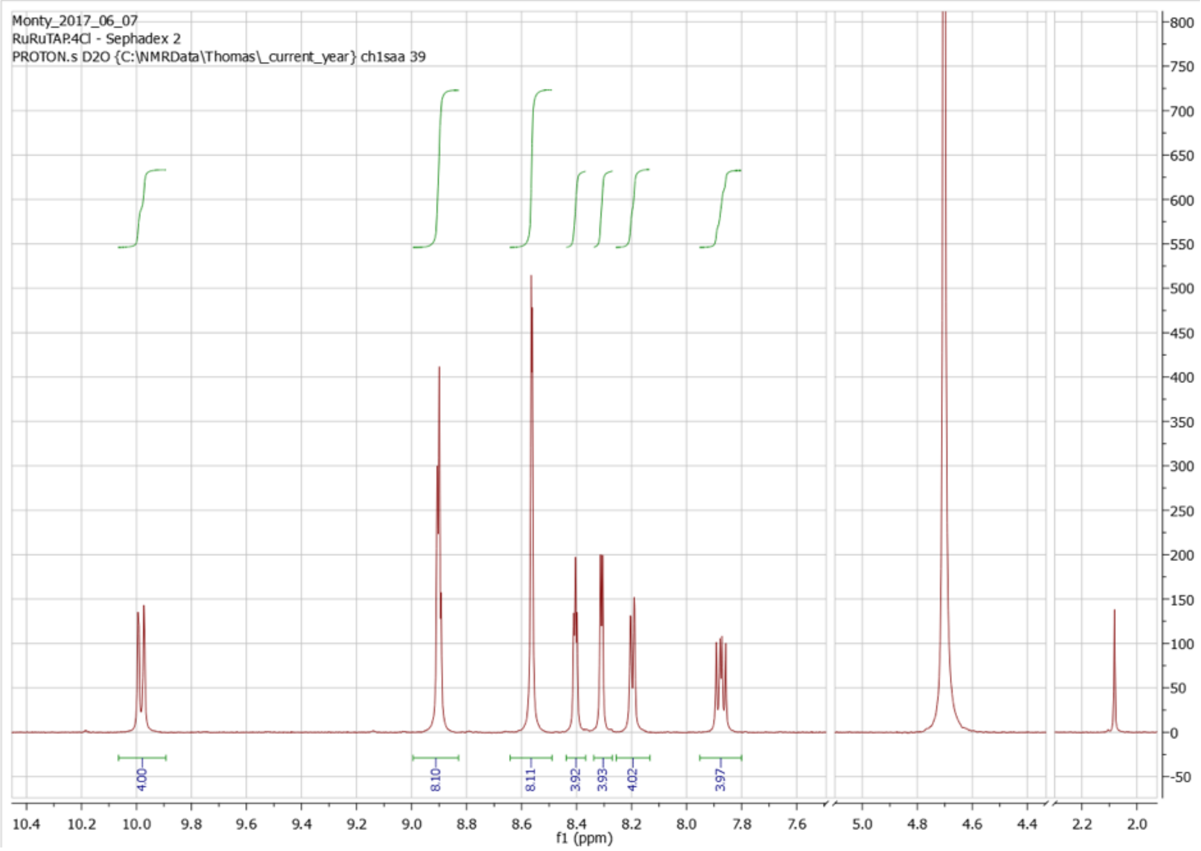

FIG. S1. ^{1}H NMR Spectrum (400 MHz) of [**2**].Cl_4 in D_2O

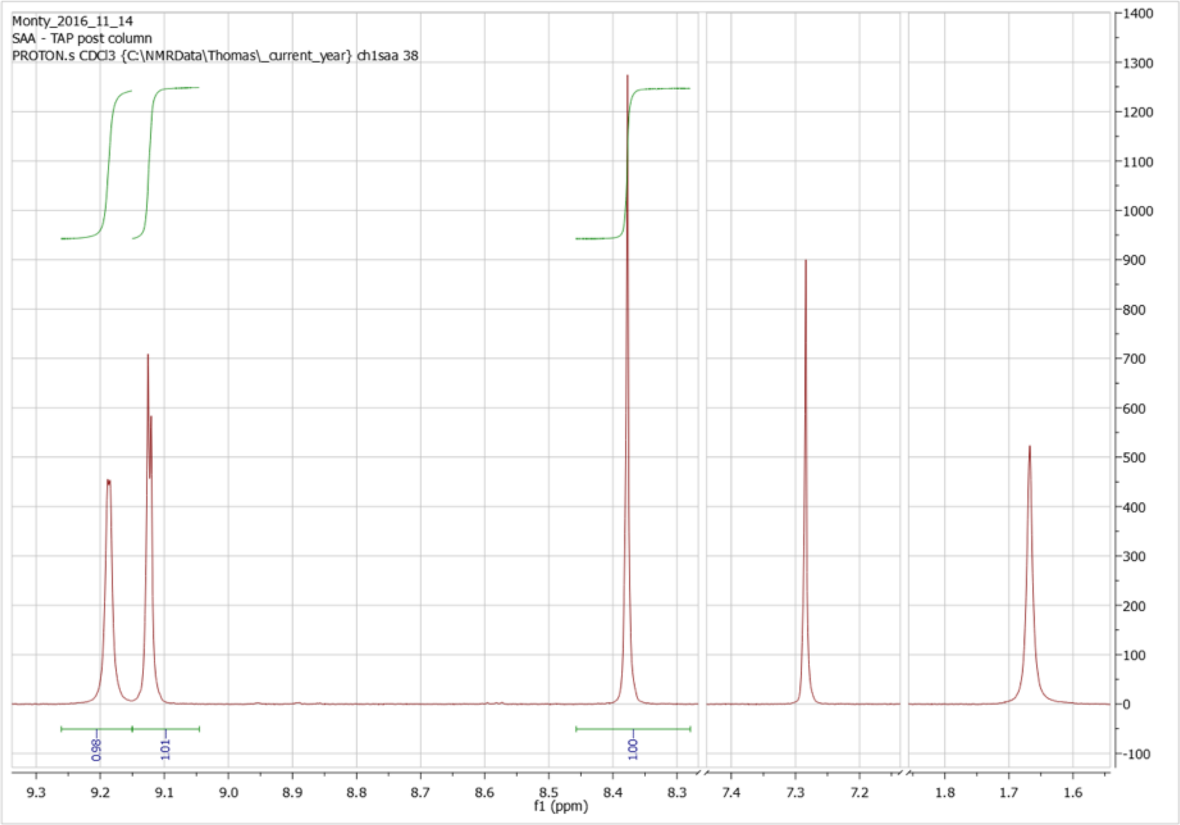

FIG. S2. ^{1}H NMR Spectrum (400 MHz) of TAP in D_2O

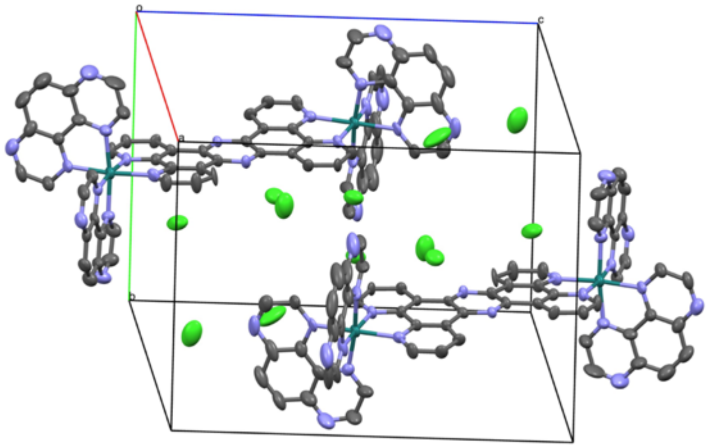

FIG. S3. Unit cell for the [**2**].Cl_4 structure

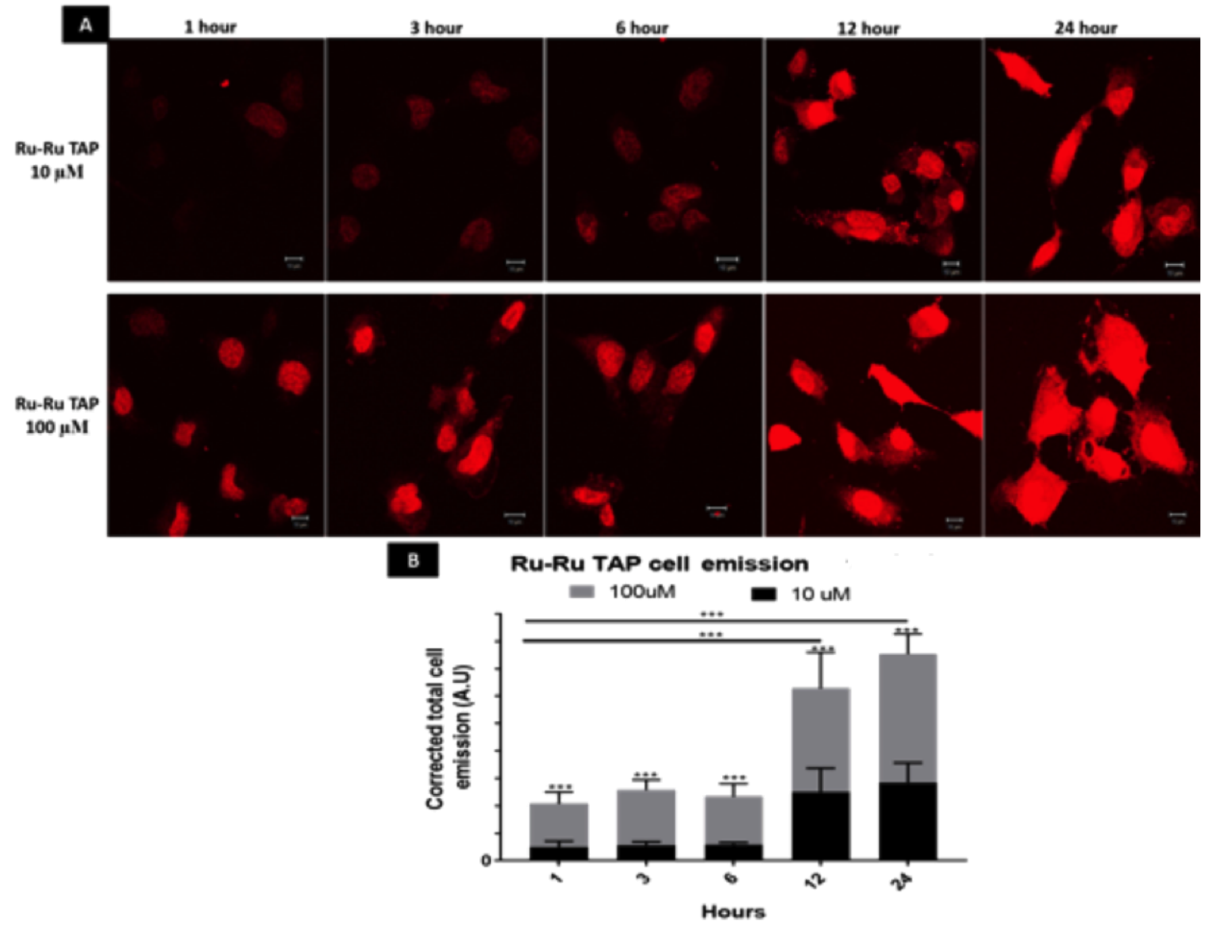

FIG. S4. Uptake of $\mathbf{2}^{4+}$ into human melanoma cells. (A) Fluorescence visualisation of Ru-Ru-TAP (10 μM and 100 μM) at different incubation end-points (1, 3, 6, 12, 24 hours), reveals the nuclear luminescence at early incubation time (1 hour) and complete distribution throughout the entirety of cells. (B) CTCE calculated in three independent images (number of cells n=9), showed statistical significant increase in emission intensity both with increase of concentration and incubation time. Highest emission was observed at 100 μM after 24 hour incubation compared to the same concentration after 1 hour (***$p<0.0001$). The emission intensity achieved at 100 μM at 1 hour can also be attained after incubating 10 μM for 12 hours.

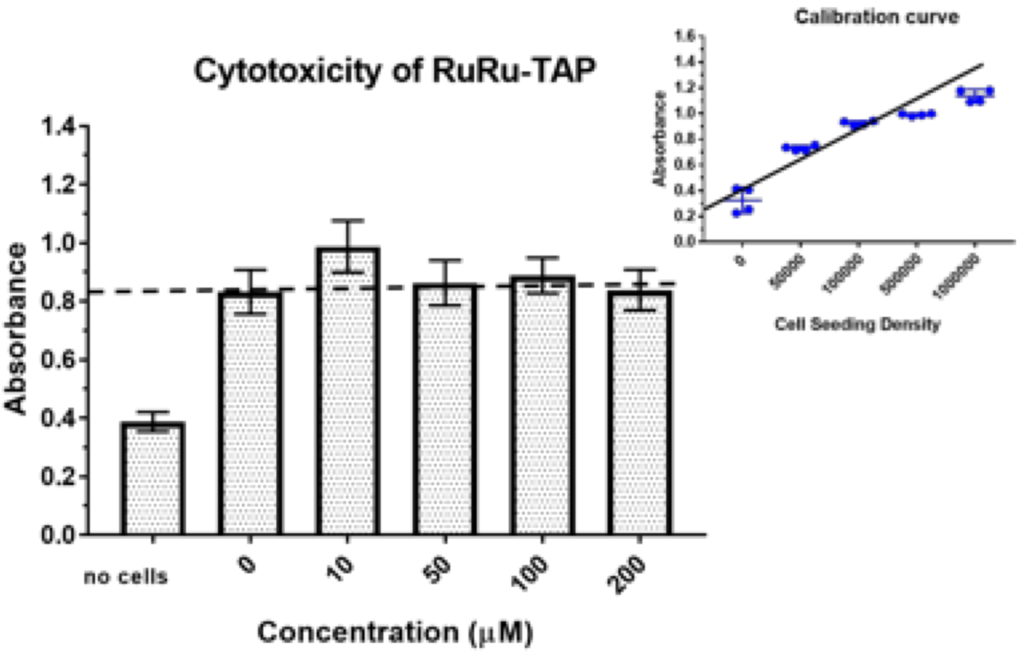

FIG. S5. Cytotoxicity of $\mathbf{2}^{4+}$ in dark, 24-hour after treatment at different concentrations (0, 10, 50, 100 and 200 μM) in a human melanoma cell line (C8161). AlamarBlue mean absorbance calculated at different initial cell seeding densities after 24 hours (right), the absorbance values used to calibrate cell concentration. No statistical significant change was observed in cell viability (left graph) (n=20) against Ru incubated cells (10 - 200 μM).

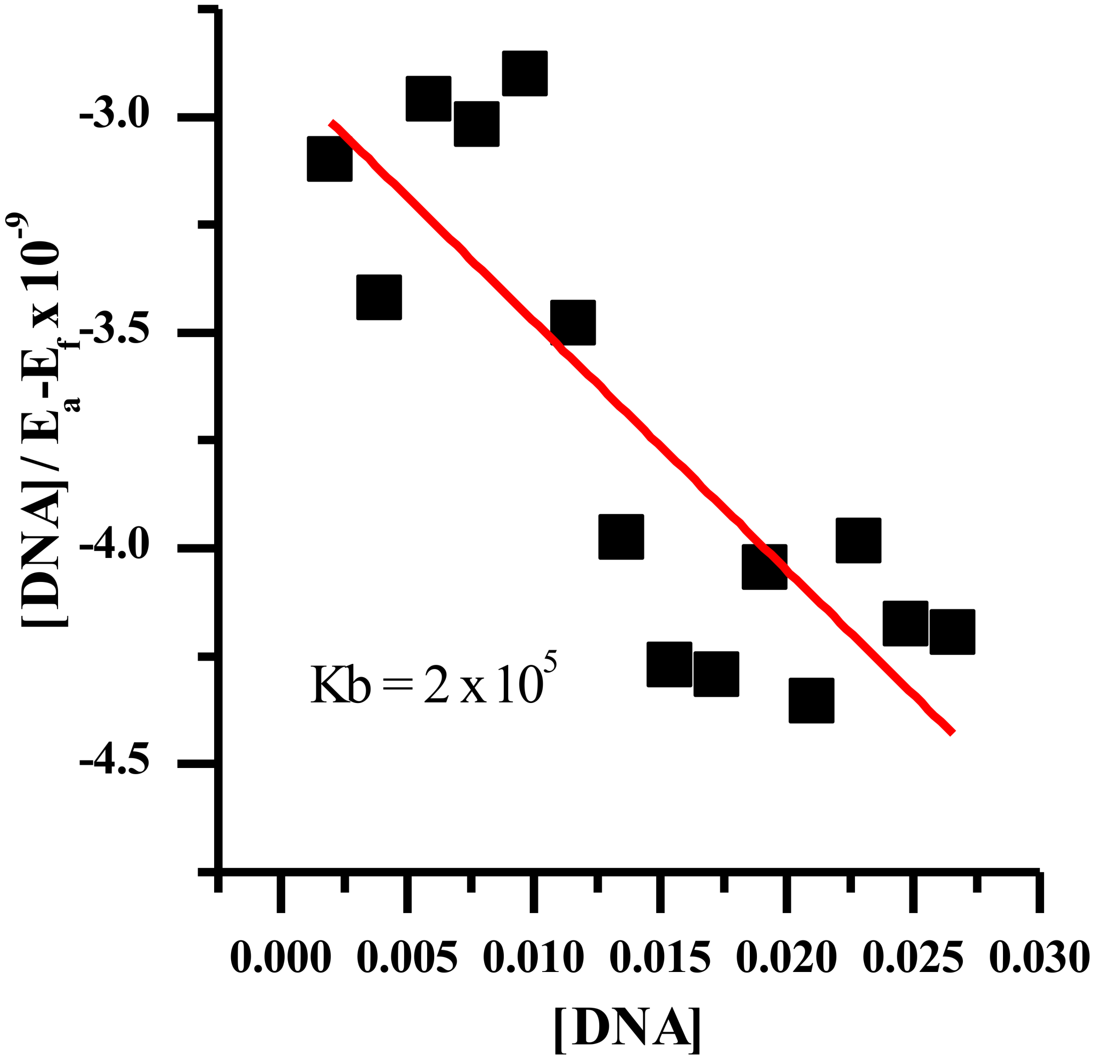

FIG. S6. Binding plot for Poly(A)-Poly(T) with [**2**].Cl_4 (5 μM concentration, in phosphate buffer) based on absorption data? red line shows fit to model developed by Srishailam, et al.[7]

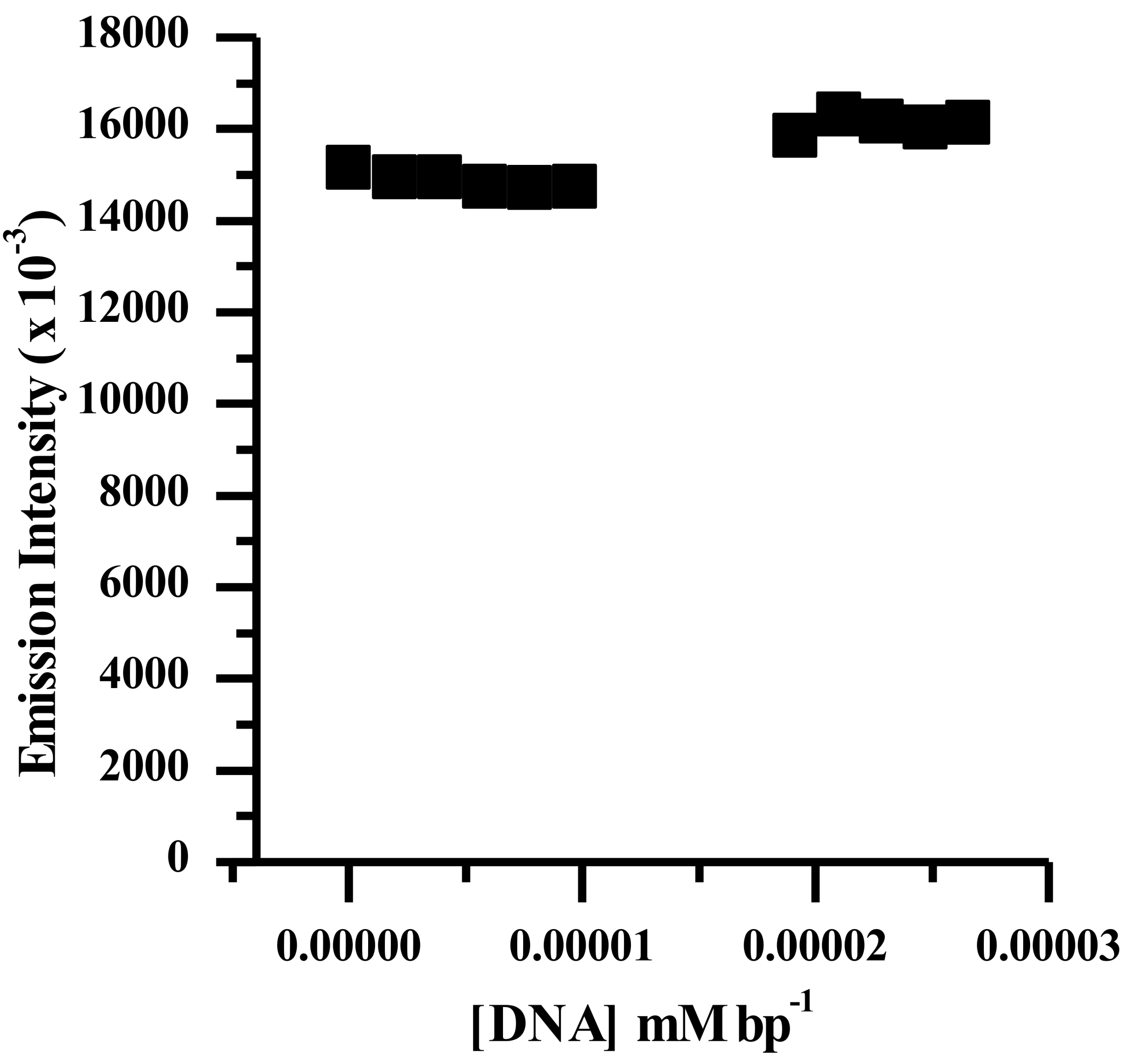

FIG. S7. Changes in maximum emission intensity of [**2**].Cl_4 (5 μM concentration in phosphate buffer) upon increasing additions of Poly(A)-Poly(T) DNA oligomers.

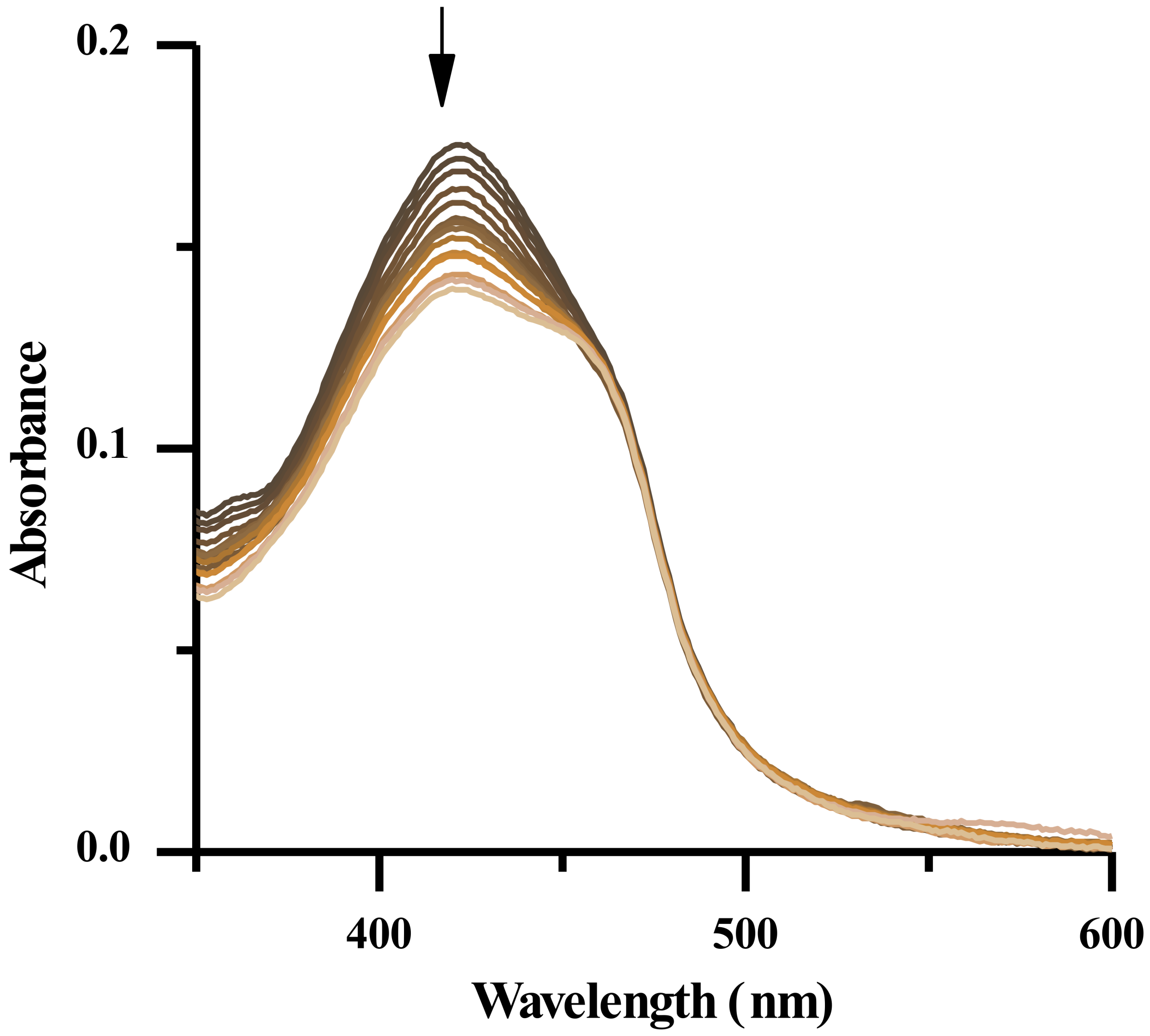

FIG. S8. CT-DNA and HTS titration data for [**2**].Cl_4 (5 μM concentration, in phosphate buffer A) Changes in absorption spectra upon addition of poly(A)-poly(T) DNA.

S1.2. Additional Computational Figures

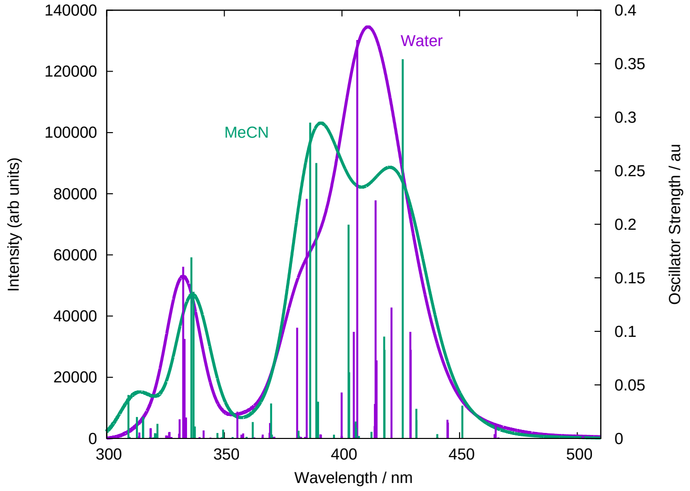

FIG. S9. Calculated UV-VIS spectra for $[\mathbf{2}]^{4+}$ in MeCN (green trace) and water (purple trace).

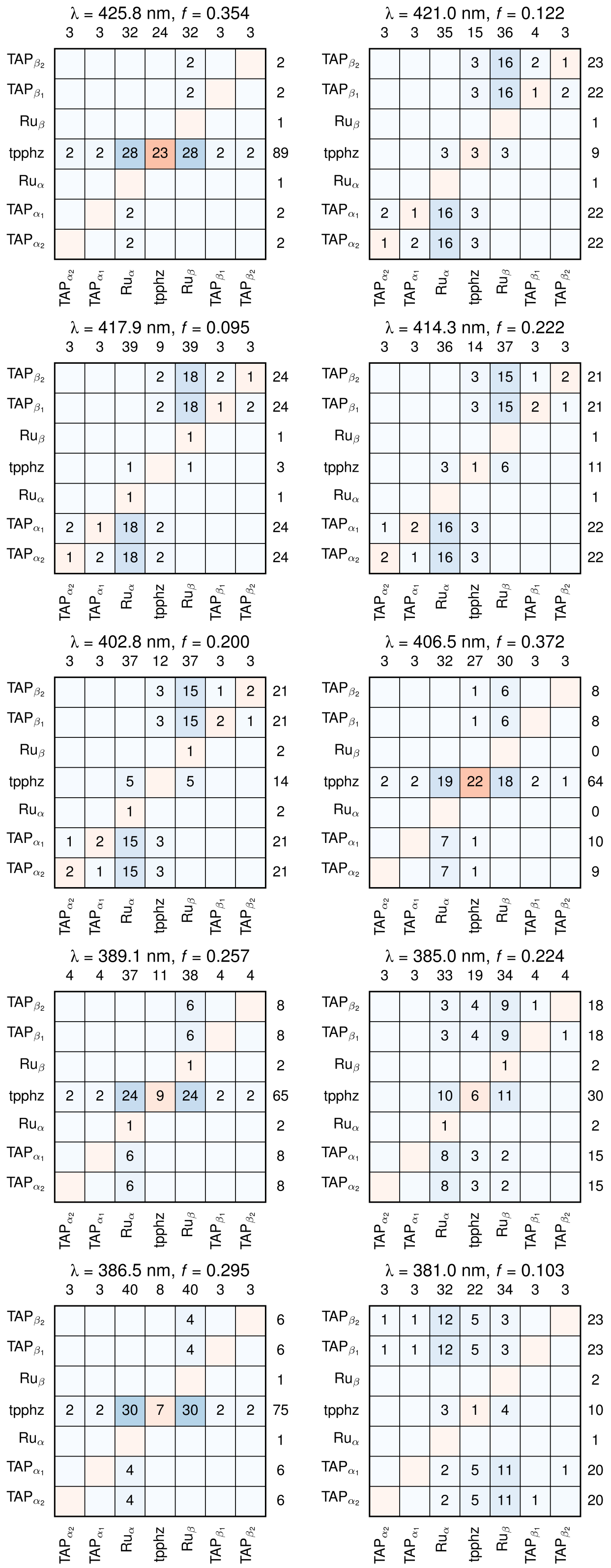

FIG. S10. Wave function analysis on the five strongest transitions in the TD-DFT calculations for compound **2** in MeCN (left-hand column) and water (right-hand column). The rows indicate what the transition is into, whereas the columns indicate what the transition is out of. In the case of water (right-hand column) all water molecules have been included with their respective ligand.

S2. MATERIALS AND METHODS

S2.1. Synthesis

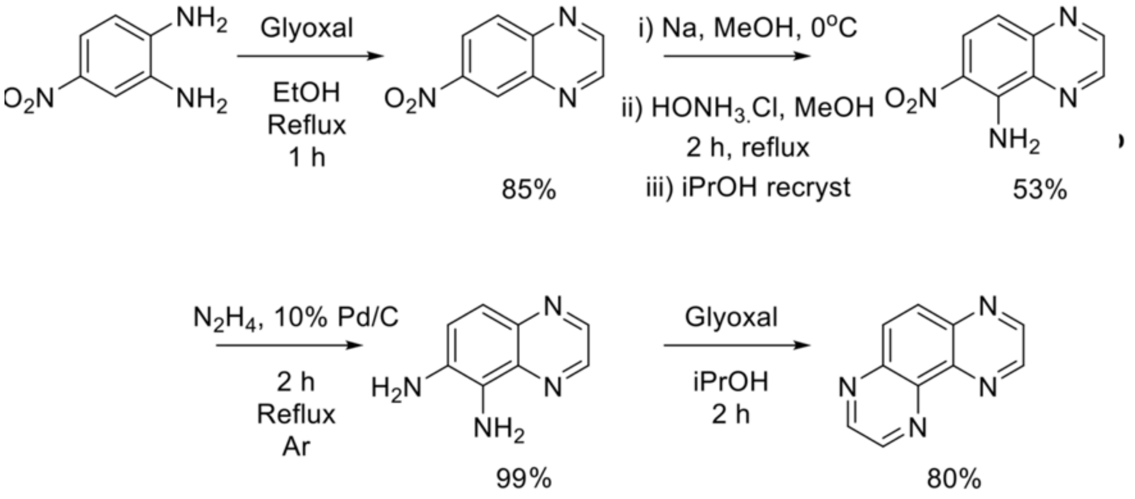

Scheme 1. Synthesis of tetraazaphenanthrene ligand

The tetraazaphenanthrene ligand was synthesised using modified literature procedures as follows:

S2.1.1. Synthesis of 4-nitroquinoxaline

1,2,-diamino-4-nitrobenzene (8 g, 55 mmol) was suspended in ethanol (125 mL) and heated to reflux. Glyoxal, 40% w/w in water (12 mL) was added to the suspension and the mixture refluxed for a further 2 hours. The resulting yellow-brown suspension was cooled rapidly in an ice bath before filtering off the precipitate, which was subsequently washed with ice cold ethanol until no further trace of the deep red starting material was apparent in the filtrate, to give a pale brown solid. Yield = 6.8 g, 70%. ^{1}H NMR (400 MHz, DMSO) δ 9.17 (s, 1H), 8.91 (d, J = 2.6 Hz, 1H), 8.57 (dd, J = 9.2, 2.6 Hz, 1H), 8.35 (d, J = 9.2 Hz, 1H).

S2.1.2. Synthesis of 4-amino-5-nitroquinoxaline

4-nitroquinoxaline (6 g, 34 mmol) was suspended in 1:1 Ethanol:Dioxane (250 mL) and heated to 60 deg C, until the solid had dissolved. The hot solution was then rapidly cooled to -10 deg C in an ice/salt bath with continuous rapid stirring to give a very fine precipitate. To this, hydroxylamine hydrochloride (14.2 g, 204 mmol) was added in one portion. Note that substantially increased yields were obtained by using freshly recrystallised hydroxylamine hydrochloride, using dry methanol as the recrystallisation solvent. To the resulting suspension, a solution of potassium hydroxide (16.3 g, 290 mmol) in methanol (200 mL) was added drop-wise over the period of 1 hour, maintaining the temperature at approximately -10 deg C using the ice/salt bath, resulting in a red-brown solution. This mixture was then stirred for a further 1 hour an -10 deg C, then stirred at room temperature for 1 hour. The resulting brown solution was immediately poured into a 1:1 ice:water mixture (1.5 L) and left in the fridge overnight. This yielded a yellow-orange precipitate, which was filtered using a Buchner funnel and washed with water (100 mL) and ethanol (100 mL). The solid was recrystallised from 3:1 glacial acetic acid:water, and finally washed with water and a small quantity of cold diethyl ether (25 mL) before drying in vacuo. Yield 3.2 g, 50%. ^{1}H NMR (400 MHz, DMSO) δ 9.09 (d, J = 1.9 Hz, 1H), 8.94 (d, J = 1.9 Hz, 1H), 8.29 (d, J = 9.7 Hz, 1H), 7.18 (d, J = 9.7 Hz, 1H).

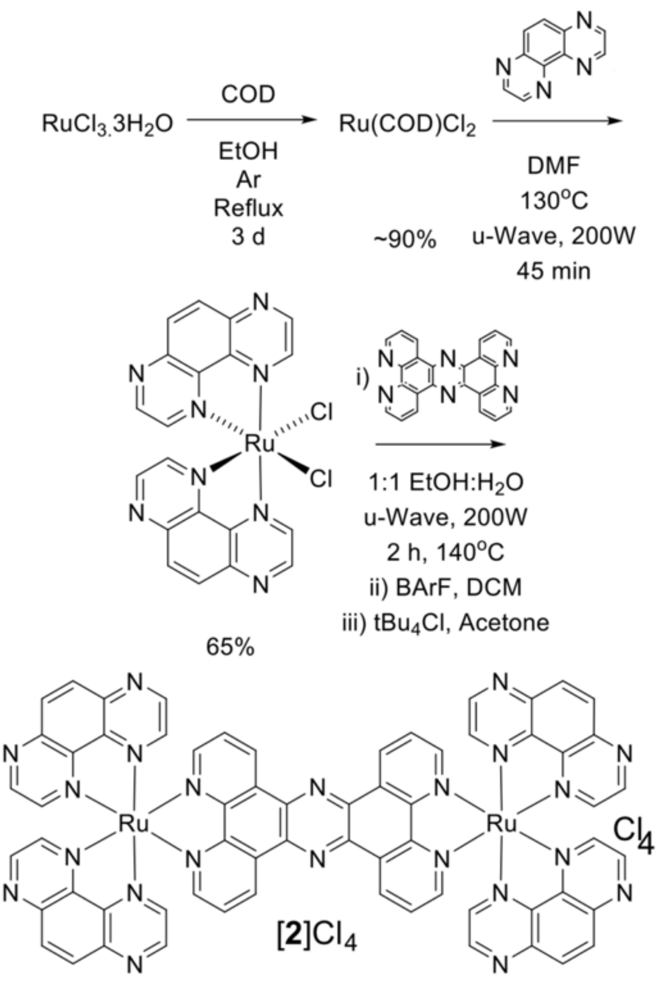

Scheme 2. Synthesis of [**2**].Cl_4 and [**2**].$(PF_6)_4$

S2.1.3. Synthesis of 4,5-diaminoquinoxaline

4-amino-5-nitroquinoxaine (1.5 g, 7.9 mmol) and 10% Pd/C (200 mg) were placed under argon. Ethanol (50 mL) was added to the mixture and heated to 65 dec C for 30 minutes. To this, hydrazine hydrate (8 mL) was added drop-wise over 15 minutes, then the reaction mixture heated for a further 2 hours. The hot reaction mixture was then filtered through a bed of celite, which was subsequently washed with dichloromethane until the filtrate was colourless. The solution was reduced to around 2-3 mL in volume on a rotary evaporator. Note that residual hydrazine hydrate is usually present after this step. This was removed by fully drying the remaining solution using a high vacuum line with a liquid nitrogen trap. The resulting red solid was used without further purification. Yield 1.2 g, 94%. 1H NMR (400 MHz, DMSO) δ 8.55 (dd, J = 31.8, 1.9 Hz, 1H), 7.23 (dd, J = 26.8, 8.8 Hz, 1H), 5.21 (d, J = 49.2 Hz, 2H).

S2.1.4. Synthesis of 1,4,5,8-tetraazaphenanthrene

5,6-diaminoquinoxaline (5 g, 31 mmol) was suspended in a mixture of 260 mL 2-propanol and 13 mL glacial acetic acid. The mixture was heated to reflux, then glyoxal, 40% w/w in water (6.8 g, 47 mmol) was added in one portion, then the mixture was further refluxed for 2 h. The dark brown solution was allowed to cool to room temperature and the solvent evaporated in vacuo to a volume of approximately 50 mL. The mixture was cooled to 4 deg C for 2 h. resulting in precipitation of the product which was filtered and washed 2-propanol (60 mL), then diethyl ether (60 mL). The precipitate was dried in vacuo before being suspended in chloroform (100 mL). The dark-brown suspension was filtered through a 2 cm Celite pad, then washed thoroughly with chloroform (300 mL). The yellow filtrate is

evaporated to yield the yellow crude product. Pure 1,4,5,8-tetraazaphenanthrene is obtained by recrystallisation from 300 mL 2-propanol as pale yellow needle crystals (3.6 g, 63%). 1H-NMR (DMSO?d6, 400 MHz): δ 8:36 (s, 2H), 9.22 ppm (d, J = 9:49 Hz, 4H)

S2.1.5. Synthesis of tetrapyridophenazine

Phenanthroline-9,10-diquinone (1.8 g, 8.5 mmol), anhydrous ammonium acetate (14.5 g, 188 mmol) and sodium thiosulfate (270 mg, 1.7 mmol) were placed under argon and heated to 185 deg C for 3 hours with gentle stirring. The reaction mixture was then cooled to around 80 deg C before water (50 mL) was added to the flask. The suspension was cooled to room temperature, filtered, and washed with water (2 x 50 mL). The crude brown solid was then suspended in ethanol (75 mL) and heated to reflux for 10 minutes. The resulting suspension was hot filtered and washed with ethanol (50 mL) and diethyl ether (50 mL) prior to drying in vacuo. Yield = 375 mg, 11%. 1H NMR (400 MHz, TFAA) δ 11.63 (s, 2H), 10.47 (d, J = 8.2 Hz, 2H), 9.51 (d, J = 4.9 Hz, 2H), 8.56 (dd, J = 8.1, 5.2 Hz, 2H).

S2.1.6. Synthesis of Ruthenium (II) 1,5-cycloocadiene dichloride

Ruthenium (III) chloride hydrate (6 g, 46 mmol) and 1,5-cyclooctadiene (20 mL, 163 mmol) were suspended in ethanol. The suspension was sparged with argon for 15 minutes, then refluxed for 3 days. The reaction mixture was then cooled to room temperature, and the resulting precipitate filtered and washed with ethanol (25 mL) and diethyl ether (25 mL) before drying in vacuo. Yield = 5.4 g, 87%. MS (MALDI) m/z 280 [M+]

S2.1.7. Synthesis of Ruthenium(II) bis(1,4,5,8-tetraazaphenanthrene)dichloride

Ruthenium (II) cyclooctadiene dichloride (1.4 g, 5 mmol) and 1,4,5,8-tetraazaphenanthrene (2.18 g, 12 mmol) were placed under argon, and dissolved in dry dimethylformamide (100 mL) The solution was heated to 130 deg C for 8 hours, then allowed to cool to room temperature. The resulting dark purple solution was poured into acetone (600 mL) and cooled in the fridge overnight. The mixture was filtered through a sintered glass funnel, washed with acetone (4 x 50 mL) and diethyl ether (50 mL), yielding a dark purple crystalline solid, which was dried overnight in vacuo. Yield = 1.85 g, 69%. 1H NMR (400 MHz, DMSO) δ 10.19 (d, J = 2.8 Hz, 2H), 9.49 (d, J = 2.8 Hz, 1H), 8.64 (d, J = 3.6 Hz, 1H), 8.63 (d, J = 2.6 Hz, 1H), 8.50 (d, J = 9.3 Hz, 1H), 8.34 (d, J = 3.0 Hz, 1H). MS (MALDI) 536 [M+]

S2.1.8. Synthesis of bisRuthenium (II) bis(1,4,5,8-tetraazaphenanthrene)tetrapydridophenazine tetrachloride [2].Cl_4

Ruthenium(II) bis(1,4,5,8-tetraazaphenanthrene)dichloride (214 mg, 0.4 mmol) and tetrapyridophenazine (30 mg, 0.08 mmol) were suspended in 1:1 ethanol:water (5 mL) in a microwave synthesis tube. The suspension was purged with argon for 15 minutes prior to sealing with a pressure cap. The reaction mixture was placed in a microwave reactor and heated to 140 deg C for 45 minutes, 100 W peak power, giving a yellow-black solution. This was diluted to 100 mL with water, and washed with dichloromethane (3 x 100 mL) to remove any residual ruthenium starting material, visible as a purple solute in the organic layer. A solution of Potassium tetrakis[3,5-bis(trifluoromethyl)phenyl]borate (KBArF, 200 mg) in dichloromethane (100 mL) was added to the separating funnel, extracting the crude product into the organic layer as the BArF salt. This process was repeated three times, then the product containing organic fraction combined, dried with magnesium sulphate and the solvent removed in vacuo. Note that the standard literature procedure of precipitation using aqueous potassium hexafluorophosphate was not possible here, as the PF_6 salt of the product shows significant solubility in water, hence it was necessary to use a less polar counteranion.

A further two-stage purification was then carried out. First, the crude product was partly purified by column chromatography on basic alumina, using 9:1:0.1 acetonitrile:water:saturated aqueous potassium nitrate eluent. Two closely-eluting orange bands were observed by TLC at Rf 0.4-0.5, assumed to be the dinuclear product and the potential mononuclear side product. These were combined and extracted with KBArF and dichloromethane as for the crude reaction mixture. Anion methathesis was carried out on the orange solid obtained by dissolving the solid in acetone and adding a large excess of tetrabutylammonium chloride to precipitate the chloride salt, which was collected by centrifugation.

The required dinuclear complex were separated by ion exchange chromatography on sephadex LH-25, using a gradient of 0.04M-0.4M aqueous sodium chloride, with the dimeric product eluting at 0.4M NaCl. The KBArF extraction and chloride anion metathesis procedure was repeated again to obtain the desired pure product as the

chloride salt. Yield = 84 mg, 0.06 mmol, 75%. ^{1}H NMR (400 MHz, D2O) δ 9.98 (d, J = 8.3 Hz, 4H), 8.90 (t, J = 2.8 Hz, 8H), 8.56 (d, J = 1.6 Hz, 8H), 8.46 ? 8.38 (m, 4H), 8.31 (d, J = 2.8 Hz, 4H), 8.20 (d, J = 5.3 Hz, 4H), 7.87 (dd, J = 8.3, 5.5 Hz, 4H). LR-MS (ESI +) m/z 329 (M^{4+}), HRMS (ESI +ve) calc. 329.0390, observed 329.0391

S2.2. Cell Work

S2.2.1. Cell culture

A C8161 human melanoma cell line was isolated from an abdominal wall metastasis from a recurrent malignant melanoma menopausal woman (developed by Professor F. Meyskens UC Irvine (USA) via Dr. M. Edwards (University Glasgow, UK))[49]. C8161 human melanoma cells were grown in melanoma culture medium consisted of EMEM media (Sigma-Aldrich) supplemented with FSC (10%v/v), L-gulamine (2 μM), Pencillin (100U/mL), streptomycin (100 μg/mL) and Amphotericin (0.625 μg/mL).

S2.2.2. Intercellular localization

Human melanoma cell line C8161 were grown in 6-well plate (initial seeding density = 1x106 cells/well) to a confluence of 60-70%, usually after 24 hours of incubation. The cells were then stained with either lysosome specific probe Lysotracker green^{TM} (ThermoFisher L7526) (100nM, 1 hour incubation in serum free media (SFM)), mitochondria specific probe Mitotracker^{TM} green (ThermoFisher M7514) (200nM, 1 hour incubation in SFM) or DAPI (300nM, 15minutes). The cells were washed with SFM and incubated with **2**.Cl_4 (100μM in SFM, 24 hours). After incubation cells were washed with SFM (thrice, 5 min) and fixed with 3.7% formaldehyde (15 minutes). Cells were washed with PBS (thrice) and left in PBS for imaging. The co-localization imaging of **2**.Cl_4 compound with cytoplasmic dye (lyso-tracker, mitotracker) and nuclear dye (DAPI) was performed using Zeiss LSM 510 META confocal upright microscopy (water dipping 40X objective, NA 0.75, WD 2.1). **2**.Cl_4 was excited with an Ar-ion laser at 458 nm and emission monitored at 670-700 nm (red). Lyso and mito tracker green was excited at 488 nm and emission at 500-550 nm. DAPI was excited using Coherent Chameleon pulsed IR multi-photon laser at 800 nm and emission detected at 435-485 nm. Image data was then processed using Zeiss LSM image browser.

S2.2.3. Cellular uptake

The uptake of **2**.Cl_4 was studied in human melanoma cell line (C8161). The cells were grown on 6 well plate for 24 hours (initial seeding density 1x106) at 37 deg C and 5% CO_2. Cells were washed and incubated with two different concentrations of **2**.Cl_4 (10 and 100 μM in SFM) for different period of incubation time (1, 3, 6, 12 and 24 hours). Ziess LSM 510 Meta confocal upright microscopy (water dipping 40X objective, NA 0.75, WD 2.1) was used to image three independent areas from each variable. Corrected total fluorescence emission was calculated from each variable using Image J and the mean values ($\pm$SD) were plotted.

S2.2.4. Light radiation source

Three different light sources were used. Firstly, ThorLabs LED (M405LP1) that has an emission of specific wavelength of 405 nm ($\pm$20nm) light, the power output of 1500 mA. The LED was fixed on a metal stand 20 cm above the base where the tissue culture dishes were placed. The three light treatment was given for 0, 1, 2 and 3 hours.

S2.2.5. Cell metabolic assay (Alamar blue)

Melanoma cells were grown in 24 well plate at an initial seeding density of 1x105 cells/well in serum media for 24 hours. Cells were then treated with different concentration of **2**.Cl_4 (0, 10, 50, 100 and 200 μM in SFM) for another 24 hours. After 24 hours, the compound was removed and the cell was washed with SFM and relished with SFM. The plates were then given light radiation using three different light sources for 0, 1, 2 and 3 hours. After irradiance dosage, the SFM was replaced with serum media and cells were incubated for 18 hours. Resazurin Na salt (Sigma R7017, 100μM for 4 hour in SFM) was used to measure cell viability. Resazurin is a non-toxic cell permeable agent

that is blue is colour but after entering into cells it reduces to resorufin that is red in colour. Viable cells unremittingly reduce resazurin leading to change in the fluorescence colour of the media. The absorbance at 560 nm was measured using a plate reader (reference point 630 nm). The average absorbance from each concentration was obtained to plot graph against **2**.Cl_4 concentration.

S2.2.6. Statistical Analysis

2way ANOVA (unpaired) were performed between non-treated and treated groups of different concentration (multiple comparison) using GraphPad Prism (software). Statistically significant value of $p \leq 0.05$ denoted with * (if ≤ 0.0001 ***) (Table S1).

S3. COMPUTATIONAL METHODS

All calculations were performed with Gaussian 09 v. D.01[34] using density-functional theory. The functional used was B3LYP[78] with empirical dispersion corrections.[79]The basis set used consisted of SDD[80] on Ru and 6-311G(d,p)[81,82] on all other atoms. All bulk solvent was described using the PCM method[83,84] as implemented in Gaussian using the provided parameters for MeCN and water. For the calculations involving water, additional water molecules were placed around the complexes coordinated to the free nitrogen atoms. In the case of TAP, the initial orientation of the additional waters was chosen randomly, since for those moieties a negligible dependence of the energetics and final electronic structure on the precise orientation of the water molecules can be expected. For the water molecules coordinated to the central tpphz unit, the initial orientation was chosen to be with the waters functioning as hydrogen-bond donors. However, during the optimisation these water molecules rotated to become hydrogen-bond acceptors. For all optimised structures frequencies were calculated in the harmonic approximation. Only small imaginary frequencies (< 12 cm^{-1}) were found. These molecules were considered to be true minima, since such small imaginary values are commonly associated with errors in the integration grids used.

All absorption spectra were calculated with the TD-DFT method[86] as implemented. Additional keywords were used to perform the wavefunction analysis using the TheoDORE 1.7.2 program.[38] Briefly, this involves partition of the one-particle transition density matrices into atomic contributions using a Löwdin partitioning.[36,37] The atomic contributions are collected into molecular fragments, in this case corresponding to the metals and ligands of the system, as well as water molecules if present. From this, a map of the excited states in terms of electron transfer between fragments can be generated, known as electron-hole correlation plots.[85] The resulting plots are created using in-house developed software, obtainable online.[Note1]

All images were created with in-house developed software, which is available upon request. The computational ESI was created using in-house developed software based on the Open Eye Toolkit.[86]

S4. ADDITIONAL TABLES

TABLE S1. Statistical significant data of Photo-toxicity measurement in melanoma cells using 405 nm light source.

Dunnett's multiple comparisons test	Mean Diff.	95.00 % CI of diff.	Significant?	Summary	Adjusted P Value
No cells					
no light treatment vs. 60 min light treatment	-0.025	-0.1117 to 0.06166	No	ns	0.8356
no light treatment vs. 120 min light treatment	-0.03558	-0.1222 to 0.05108	No	ns	0.6460
no light treatment vs. 180 min light treatment	-0.03867	-0.1253 to 0.048	No	ns	0.5869
0					
no light treatment vs. 60 min light treatment	0.04967	-0.037 to 0.1363	No	ns	0.3894
no light treatment vs. 120 min light treatment	0.05792	-0.02875 to 0.1446	No	ns	0.2686
no light treatment vs. 180 min light treatment	0.1033	0.01659 to 0.1899	Yes	*	0.0146
10					
no light treatment vs. 60 min light treatment	0.08067	-0.005995 to 0.1673	No	ns	0.0745
no light treatment vs. 120 min light treatment	0.2262	0.1395 to 0.3128	Yes	****	0.0001
no light treatment vs. 180 min light treatment	0.4334	0.3468 to 0.5201	Yes	****	0.0001
50					
no light treatment vs. 60 min light treatment	0.5018	0.4152 to 0.5885	Yes	****	0.0001
no light treatment vs. 120 min light treatment	0.7157	0.629 to 0.8024	Yes	****	0.0001
no light treatment vs. 180 min light treatment	0.7312	0.6445 to 0.8179	Yes	****	0.0001
100					
no light treatment vs. 60 min light treatment	0.4824	0.3957 to 0.569	Yes	****	0.0001
no light treatment vs. 120 min light treatment	0.6443	0.5577 to 0.731	Yes	****	0.0001
no light treatment vs. 180 min light treatment	0.7058	0.6191 to 0.7924	Yes	****	0.0001
200					
no light treatment vs. 60 min light treatment	0.4824	0.3958 to 0.5691	Yes	****	0.0001
no light treatment vs. 120 min light treatment	0.589	0.5023 to 0.6757	Yes	****	0.0001
no light treatment vs. 180 min light treatment	0.762	0.6753 to 0.8487	Yes	****	0.0001

S5. CALCULATIONS ON 1^{4+} (^{1}A) IN MeCN

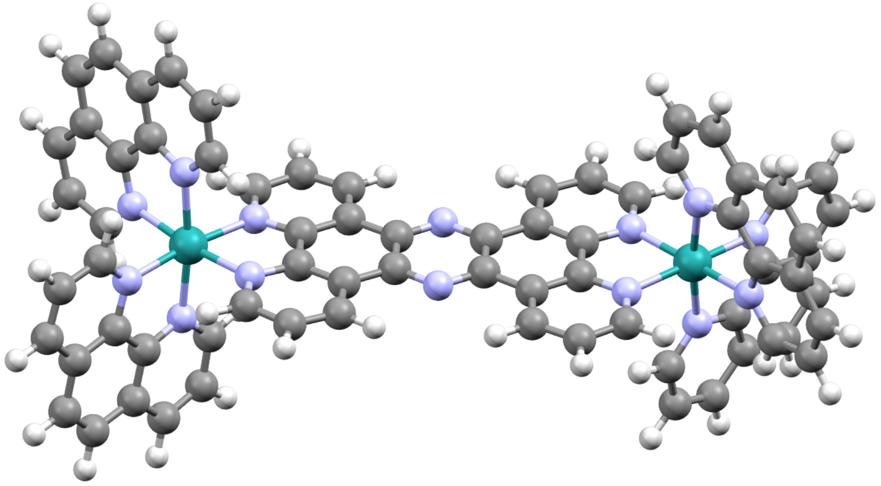

Route	: # opt freq b3lyp/genecp scrf=(solvent=acetonitrile) geom=connectivity
	: int=ultrafine scf=tight volume
SMILES	: c1cc2ccc3ccc[n+]4c3c2[n+](c1)[Ru]456([n+]7cccc8c7c9[n+]5cccc9cc8)[n+]1cccc2c1c1[n+]6cccc1c1c2nc2c3ccc[n+]4c3c3c(c2n1)ccc[n+]3[Ru]412([n+]3cccc4c3c3[n+]1cccc3cc4)[n+]1cccc3c1c1[n+]2cccc1cc3
Formula	: $C_{72}H_{44}N_{14}Ru_2^{4+}$
Charge	: 4
Multiplicity	: 1
Energy	: -3727.25165764 a.u.
Gibbs Energy	: -3726.33821500 a.u.
Number of imaginary frequencies	: 0

S5.1. Cartesian Co-ordinates (XYZ format)

132

Ru	6.45601511	0.00001800	0.00001600
N	4.82583094	0.04382200	-1.33113503
N	0.00005500	-0.00109300	-1.39486098
N	0.00004800	0.00068200	1.39486003
N	4.82582712	-0.04393800	1.33115101
C	4.86504221	0.07284000	-2.66823196
H	5.84581614	0.09529200	-3.12218308
C	3.71124411	0.08031400	-3.45436597
H	3.80691099	0.10560100	-4.53150177
C	2.47106791	0.05718700	-2.84514999
H	1.55671096	0.06507400	-3.42242503
C	2.40463209	0.02508700	-1.44447196
C	1.14391899	0.00505000	-0.71166998
C	-1.14381599	-0.00635900	-0.71166903
C	-3.60768604	-0.01773300	-0.72212398
C	-3.60769892	0.01754100	0.72210002
C	-2.40453005	0.02690100	1.44444203
C	-1.14382100	0.00596800	0.71166301

C	1.14391601	-0.00545700	0.71167302
C	2.40462589	-0.02542100	1.44447899
C	2.47105908	-0.05751400	2.84515595
H	1.55669999	-0.06547600	3.42242789
C	3.71123505	-0.08053400	3.45437789
H	3.80689692	-0.10581300	4.53151417
C	4.86503506	-0.07294700	2.66825104
H	5.84580994	-0.09529000	3.12220502
C	3.60780692	-0.01690500	0.72214502
C	3.60780907	0.01667000	-0.72213298
C	7.50706387	-2.45239401	-1.21759295
C	5.89064407	-3.04680991	0.33185399
C	7.74928522	-3.79845905	-1.56658804
C	8.24379444	-1.40507197	-1.85178101
C	6.06640005	-4.40681601	0.04068200
H	5.17304277	-2.73086095	1.07634997
C	6.99270582	-4.78832722	-0.90609002
C	9.21852875	-1.71536195	-2.82415509
H	5.46865702	-5.13741207	0.56918001
H	7.14566517	-5.83365583	-1.14567304
C	8.63653469	0.86959600	-2.04800200
C	9.91624641	-0.64058000	-3.41265702
C	9.62258339	0.64752698	-3.01953602
H	8.39078331	1.87382400	-1.73128104
H	10.67242432	-0.83542198	-4.16355324
H	10.13802242	1.49723697	-3.44689894
N	7.95584679	-0.12334000	-1.47494805
N	6.58780718	-2.08607197	-0.27493000
C	8.24374008	1.40524995	1.85177696
C	8.63657761	-0.86939901	2.04804111
C	9.21849442	1.71560395	2.82411909
C	7.50693083	2.45250511	1.21757603
C	9.62264633	-0.64726698	3.01954103
H	8.39086246	-1.87364697	1.73135400
C	9.91627312	0.64086199	3.41261911
C	7.74905491	3.79857993	1.56656003
H	10.13813686	-1.49694705	3.44689989
H	10.67248535	0.83575201	4.16346788
C	5.89044285	3.04679608	-0.33184600
C	6.99240017	4.78840017	0.90607899
C	6.06611300	4.40681887	-0.04068600
H	5.17283392	2.73079991	-1.07631302
H	7.14527607	5.83373785	1.14567399
H	5.46830797	5.13736820	-0.56917900
N	7.95583582	0.12349400	1.47497904
N	6.58767986	2.08610702	0.27493501
C	-4.86496878	-0.07598700	-2.66813493
C	-4.86497116	0.07598300	2.66810894
C	-2.40452003	-0.02720000	-1.44445503
C	-2.47098303	-0.06099100	-2.84509611
H	-1.55663800	-0.06971300	-3.42237711
C	-2.47099304	0.06072500	2.84507799
H	-1.55665302	0.06936200	3.42236900
C	-3.71117210	-0.08464600	-3.45426393
C	-3.71118093	0.08453200	3.45424700
H	-3.80686307	-0.11118300	-4.53136778
H	-5.84575319	-0.09872400	-3.12205696
H	-3.80685902	0.11112000	4.53135014
H	-5.84576416	0.09885200	3.12200809
N	-4.82572794	-0.04537500	-1.33107400
N	-4.82573318	0.04532100	1.33105099
Ru	-6.45587015	0.00001100	-0.00003200
N	-7.95581818	0.12207100	-1.47507799
N	-6.58757877	2.08580804	-0.27694601

N	-7.95580101	-0.12198900	1.47499299
N	-6.58761215	-2.08579206	0.27691501
C	-8.24389839	1.40349197	-1.85288703
C	-8.63651371	-0.87135899	-2.04722905
C	-7.50704193	2.45133591	-1.21970499
C	-5.89020109	3.04703903	0.32879999
C	-8.24386215	-1.40339696	1.85290098
C	-8.63650131	0.87146199	2.04711699
C	-7.50702381	-2.45127606	1.21975303
C	-5.89029503	-3.04706097	-0.32883301
C	-9.21883678	1.71295094	-2.82533407
C	-9.62278557	-0.65012699	-3.01873994
H	-8.39060211	-1.87532794	-1.72980106
C	-7.74933004	3.79708505	-1.56978703
C	-6.06596422	4.40680122	0.03644800
H	-5.17243004	2.73172212	1.07340002
C	-9.21876335	-1.71280503	2.82538700
C	-9.62271309	0.65027303	3.01869202
H	-8.39063644	1.87541795	1.72961605
C	-7.74930191	-3.79700899	1.56990504
C	-6.06606722	-4.40681219	-0.03643000
H	-5.17256212	-2.73179412	-1.07349205
C	-9.91661930	0.63764298	-3.41280890
H	-10.13825703	-1.50021303	-3.44531608
C	-6.99252081	4.78751802	-0.91039699
H	-5.46801376	5.13783693	0.56410497
C	-9.91651726	-0.63747603	3.41285491
H	-10.13816547	1.50038505	3.44524097
C	-6.99257183	-4.78747988	0.91048402
H	-5.46818399	-5.13787413	-0.56412601
H	-10.67298603	0.83185101	-4.16368198
H	-7.14548588	5.83264208	-1.15088403
H	-7.14554691	-5.83259392	1.15101004
H	-10.67280769	-0.83164400	4.16381311
C	9.44720364	3.09116411	3.16045809
H	10.19758606	3.32033491	3.90747404
C	8.74241638	4.08806992	2.56018496
H	8.92262173	5.12390089	2.82173109
C	8.74269295	-4.08794880	-2.56017995
H	8.92328262	-5.12381792	-2.82131290
C	9.44720268	-3.09090400	-3.16056490
H	10.19778919	-3.32009792	-3.90737009
C	-8.74293041	-4.08548594	2.56354904
H	-8.92381573	-5.12109518	2.82549906
C	-9.44760323	-3.08803701	3.16299891
H	-10.19849682	-3.31647205	3.90972805
C	-8.74303436	4.08559608	-2.56333899
H	-8.92337227	5.12116003	-2.82583809
C	-9.44773483	3.08819389	-3.16288304
H	-10.19809628	3.31657505	-3.91016507

S5.2. Frequencies

Mode	IR frequency	IR intensity	Raman intensity
1	7.67400000	0.00000000	0.00000000
2	10.52910000	0.09820000	0.00000000
3	15.07570000	0.15700000	0.00000000
4	24.07720000	0.00020000	0.00000000
5	24.86730000	0.40400000	0.00000000
6	26.97100000	0.81520000	0.00000000
7	29.55870000	0.00120000	0.00000000
8	37.82110000	0.19210000	0.00000000
9	38.42220000	0.04160000	0.00000000
10	42.69660000	0.15780000	0.00000000
11	43.51960000	0.00010000	0.00000000
12	44.27950000	0.47570000	0.00000000
13	48.97790000	0.21440000	0.00000000
14	53.69720000	0.00000000	0.00000000
15	65.02950000	0.10640000	0.00000000
16	71.50080000	0.28840000	0.00000000
17	73.94600000	0.00010000	0.00000000
18	85.54560000	8.33090000	0.00000000
19	85.79910000	6.63740000	0.00000000
20	88.34410000	3.00130000	0.00000000
21	93.94250000	4.83660000	0.00000000
22	100.61960000	0.00000000	0.00000000
23	134.39890000	0.00060000	0.00000000
24	146.89810000	0.00180000	0.00000000
25	151.52830000	0.01670000	0.00000000
26	161.54810000	1.08460000	0.00000000
27	167.21470000	0.53250000	0.00000000
28	167.25200000	0.52350000	0.00000000
29	169.78910000	0.00010000	0.00000000
30	170.13260000	8.58580000	0.00000000
31	171.04430000	0.00000000	0.00000000
32	172.91430000	0.00630000	0.00000000
33	180.25110000	0.15110000	0.00000000
34	180.77130000	6.51350000	0.00000000
35	185.05860000	0.00000000	0.00000000
36	187.20830000	1.42940000	0.00000000
37	189.24750000	1.56240000	0.00000000
38	190.32670000	3.52220000	0.00000000
39	199.68900000	1.47720000	0.00000000
40	201.60990000	0.00000000	0.00000000
41	226.46100000	0.26110000	0.00000000
42	233.25470000	0.00820000	0.00000000
43	234.01830000	8.04560000	0.00000000
44	236.28730000	0.00010000	0.00000000
45	236.54020000	0.25390000	0.00000000
46	236.70570000	8.35020000	0.00000000
47	239.92230000	0.46850000	0.00000000
48	250.28030000	0.71550000	0.00000000
49	276.15130000	2.09490000	0.00000000
50	276.70340000	2.82300000	0.00000000
51	277.61540000	13.55790000	0.00000000
52	278.77150000	0.00020000	0.00000000
53	286.35930000	1.98720000	0.00000000
54	286.76420000	1.46820000	0.00000000
55	288.65160000	0.75550000	0.00000000
56	288.94720000	0.00030000	0.00000000
57	304.49960000	0.00000000	0.00000000
58	315.21360000	0.86940000	0.00000000
59	316.19350000	1.69120000	0.00000000
60	316.39760000	2.68470000	0.00000000

61	322.50990000	0.00000000	0.00000000
62	332.53580000	1.37970000	0.00000000
63	335.08210000	0.06920000	0.00000000
64	336.22990000	0.01390000	0.00000000
65	352.87350000	0.36920000	0.00000000
66	383.01810000	0.00000000	0.00000000
67	396.74580000	0.01100000	0.00000000
68	432.40530000	16.43340000	0.00000000
69	435.82000000	0.00000000	0.00000000
70	436.28680000	1.49320000	0.00000000
71	436.37680000	1.14870000	0.00000000
72	437.29240000	0.04540000	0.00000000
73	437.45550000	0.00000000	0.00000000
74	438.69140000	0.11380000	0.00000000
75	439.63120000	0.00030000	0.00000000
76	445.66170000	0.00000000	0.00000000
77	445.87850000	0.46440000	0.00000000
78	448.66580000	2.71190000	0.00000000
79	459.82230000	14.82610000	0.00000000
80	463.20450000	0.12730000	0.00000000
81	471.17190000	1.27170000	0.00000000
82	474.64580000	0.00040000	0.00000000
83	474.78750000	0.63350000	0.00000000
84	476.25960000	2.04220000	0.00000000
85	477.69900000	2.13920000	0.00000000
86	481.55320000	3.51240000	0.00000000
87	494.35460000	0.00000000	0.00000000
88	498.59290000	3.74800000	0.00000000
89	502.96030000	0.20140000	0.00000000
90	504.02980000	1.26360000	0.00000000
91	506.93520000	0.00000000	0.00000000
92	520.27400000	0.98070000	0.00000000
93	520.30900000	0.97780000	0.00000000
94	520.31620000	2.55480000	0.00000000
95	520.32470000	3.12560000	0.00000000
96	529.79620000	25.69170000	0.00000000
97	536.72130000	17.25150000	0.00000000
98	538.31790000	0.55760000	0.00000000
99	541.32300000	9.15240000	0.00000000
100	542.08090000	0.00010000	0.00000000
101	550.77070000	0.23050000	0.00000000
102	564.61650000	0.00260000	0.00000000
103	564.70180000	0.21640000	0.00000000
104	565.73790000	0.00270000	0.00000000
105	565.79300000	0.13280000	0.00000000
106	568.63370000	5.73710000	0.00000000
107	568.64980000	2.27560000	0.00000000
108	569.40710000	1.83010000	0.00000000
109	569.40970000	1.22310000	0.00000000
110	571.03920000	0.00010000	0.00000000
111	575.09510000	0.21410000	0.00000000
112	588.44580000	0.29070000	0.00000000
113	596.55060000	14.42850000	0.00000000
114	622.32940000	0.00050000	0.00000000
115	631.43570000	0.12120000	0.00000000
116	632.27740000	0.07660000	0.00000000
117	634.26720000	0.00070000	0.00000000
118	634.31020000	0.22800000	0.00000000
119	654.40790000	0.00000000	0.00000000
120	656.74980000	0.56330000	0.00000000
121	657.22260000	2.42070000	0.00000000
122	659.55760000	4.96690000	0.00000000
123	661.56730000	1.46550000	0.00000000
124	661.69980000	7.64190000	0.00000000

125	661.73280000	0.03030000	0.00000000
126	666.25580000	1.02760000	0.00000000
127	712.82080000	0.09360000	0.00000000
128	727.53200000	0.00000000	0.00000000
129	732.76840000	0.00000000	0.00000000
130	734.39960000	30.57800000	0.00000000
131	737.92690000	0.05820000	0.00000000
132	737.93490000	13.42220000	0.00000000
133	738.47340000	0.01690000	0.00000000
134	738.50780000	24.97400000	0.00000000
135	740.85690000	131.03740000	0.00000000
136	740.97380000	5.55840000	0.00000000
137	742.75260000	111.91030000	0.00000000
138	742.84190000	0.16110000	0.00000000
139	744.64420000	0.61730000	0.00000000
140	744.96580000	8.86810000	0.00000000
141	751.02920000	0.00000000	0.00000000
142	751.26870000	1.29270000	0.00000000
143	752.25480000	120.52530000	0.00000000
144	753.94600000	2.83740000	0.00000000
145	756.19300000	0.41300000	0.00000000
146	788.82150000	23.16490000	0.00000000
147	788.83970000	12.90670000	0.00000000
148	788.92220000	26.65310000	0.00000000
149	788.94050000	7.76530000	0.00000000
150	811.25080000	0.00620000	0.00000000
151	812.31850000	0.32500000	0.00000000
152	812.34640000	0.04350000	0.00000000
153	812.45200000	0.06410000	0.00000000
154	812.46680000	0.14550000	0.00000000
155	817.88010000	0.00000000	0.00000000
156	819.47380000	0.00000000	0.00000000
157	823.40940000	0.10770000	0.00000000
158	834.30200000	129.09020000	0.00000000
159	838.65880000	0.13620000	0.00000000
160	852.19560000	0.11130000	0.00000000
161	852.19880000	0.00320000	0.00000000
162	852.21080000	0.54620000	0.00000000
163	853.47610000	0.40480000	0.00000000
164	854.29560000	0.00000000	0.00000000
165	864.52590000	213.88170000	0.00000000
166	864.67620000	0.11830000	0.00000000
167	864.71130000	26.33490000	0.00000000
168	864.72980000	186.14920000	0.00000000
169	865.13190000	11.16850000	0.00000000
170	874.18740000	0.04880000	0.00000000
171	889.82130000	0.36420000	0.00000000
172	889.84260000	0.04770000	0.00000000
173	891.89770000	0.00020000	0.00000000
174	891.97930000	0.29560000	0.00000000
175	925.85660000	5.24670000	0.00000000
176	926.01900000	4.51150000	0.00000000
177	928.02820000	0.01670000	0.00000000
178	928.05370000	0.28020000	0.00000000
179	938.64030000	0.20540000	0.00000000
180	969.99710000	0.09660000	0.00000000
181	970.03520000	0.10060000	0.00000000
182	970.38130000	0.01480000	0.00000000
183	970.42120000	0.01130000	0.00000000
184	974.02390000	0.13550000	0.00000000
185	974.04710000	0.13780000	0.00000000
186	974.15480000	0.47560000	0.00000000
187	974.17830000	0.39210000	0.00000000
188	982.48540000	0.03200000	0.00000000

189	982.62640000	0.02980000	0.00000000
190	983.18570000	0.00000000	0.00000000
191	983.74280000	0.08920000	0.00000000
192	1001.20890000	0.01170000	0.00000000
193	1001.22700000	0.02820000	0.00000000
194	1001.23010000	0.01140000	0.00000000
195	1001.24950000	0.02680000	0.00000000
196	1016.82470000	0.99820000	0.00000000
197	1016.83040000	0.89180000	0.00000000
198	1016.86920000	1.13250000	0.00000000
199	1016.87700000	0.99000000	0.00000000
200	1019.33250000	0.02000000	0.00000000
201	1019.33790000	0.01820000	0.00000000
202	1019.34210000	0.04890000	0.00000000
203	1019.34940000	0.04600000	0.00000000
204	1025.06260000	0.78880000	0.00000000
205	1025.78610000	0.00810000	0.00000000
206	1026.02910000	0.02590000	0.00000000
207	1026.66560000	0.00000000	0.00000000
208	1041.05810000	7.36600000	0.00000000
209	1050.56970000	0.00000000	0.00000000
210	1051.88780000	1.24250000	0.00000000
211	1052.25930000	0.49590000	0.00000000
212	1053.79860000	0.04170000	0.00000000
213	1054.49990000	0.00000000	0.00000000
214	1056.13570000	0.05870000	0.00000000
215	1061.06330000	1.75320000	0.00000000
216	1079.20800000	7.84770000	0.00000000
217	1079.77350000	3.19820000	0.00000000
218	1079.77770000	1.47540000	0.00000000
219	1081.03880000	0.00000000	0.00000000
220	1081.25640000	9.65660000	0.00000000
221	1096.34340000	0.00000000	0.00000000
222	1113.26790000	2.24990000	0.00000000
223	1113.27990000	0.43290000	0.00000000
224	1114.03940000	5.12860000	0.00000000
225	1114.55530000	3.74020000	0.00000000
226	1115.66460000	9.81400000	0.00000000
227	1118.55110000	19.34660000	0.00000000
228	1121.36000000	8.81890000	0.00000000
229	1124.56300000	0.77720000	0.00000000
230	1124.57180000	0.62900000	0.00000000
231	1128.07070000	0.00000000	0.00000000
232	1129.09840000	1.67020000	0.00000000
233	1146.75050000	0.00000000	0.00000000
234	1155.52500000	212.59400000	0.00000000
235	1157.48070000	18.67150000	0.00000000
236	1158.06370000	0.12850000	0.00000000
237	1169.49630000	15.11700000	0.00000000
238	1169.49910000	13.54810000	0.00000000
239	1169.76110000	9.27390000	0.00000000
240	1169.76430000	2.83930000	0.00000000
241	1172.83710000	1.48630000	0.00000000
242	1172.84050000	1.34280000	0.00000000
243	1172.95670000	1.67150000	0.00000000
244	1172.95890000	0.54910000	0.00000000
245	1203.48560000	5.24540000	0.00000000
246	1218.43760000	0.00000000	0.00000000
247	1222.17050000	5.31480000	0.00000000
248	1227.94060000	2.93030000	0.00000000
249	1227.94340000	3.67160000	0.00000000
250	1229.16600000	0.02280000	0.00000000
251	1229.22190000	0.00000000	0.00000000
252	1231.46440000	7.22040000	0.00000000

253	1231.48890000	5.10780000	0.00000000
254	1232.86660000	4.53130000	0.00000000
255	1232.93100000	0.19820000	0.00000000
256	1248.25950000	0.97010000	0.00000000
257	1248.26480000	0.92070000	0.00000000
258	1248.55000000	5.80230000	0.00000000
259	1248.57610000	1.67490000	0.00000000
260	1254.58650000	0.46960000	0.00000000
261	1277.27360000	21.91470000	0.00000000
262	1281.85180000	2.06340000	0.00000000
263	1281.87330000	1.69540000	0.00000000
264	1283.16110000	0.36470000	0.00000000
265	1283.18760000	1.16460000	0.00000000
266	1294.09480000	0.00000000	0.00000000
267	1307.59280000	0.30990000	0.00000000
268	1315.03000000	22.60400000	0.00000000
269	1316.37200000	3.93540000	0.00000000
270	1327.77900000	0.07450000	0.00000000
271	1330.04490000	0.52630000	0.00000000
272	1330.08140000	1.22710000	0.00000000
273	1330.22280000	0.00850000	0.00000000
274	1330.37570000	0.52060000	0.00000000
275	1330.39120000	0.59430000	0.00000000
276	1340.91310000	0.00010000	0.00000000
277	1342.60240000	6.45420000	0.00000000
278	1342.61640000	6.21680000	0.00000000
279	1342.63410000	11.25390000	0.00000000
280	1343.15900000	0.00200000	0.00000000
281	1349.20910000	56.65600000	0.00000000
282	1366.76750000	12.01130000	0.00000000
283	1366.78630000	12.44730000	0.00000000
284	1367.79300000	18.15230000	0.00000000
285	1367.82810000	2.06980000	0.00000000
286	1384.06120000	417.66920000	0.00000000
287	1384.70070000	0.00030000	0.00000000
288	1425.84950000	153.11200000	0.00000000
289	1440.50560000	89.84760000	0.00000000
290	1440.58150000	40.52190000	0.00000000
291	1441.79760000	20.44990000	0.00000000
292	1441.80610000	1.33460000	0.00000000
293	1447.82710000	31.21360000	0.00000000
294	1447.84690000	31.26200000	0.00000000
295	1448.14210000	17.90190000	0.00000000
296	1448.16240000	13.21790000	0.00000000
297	1449.16210000	0.72930000	0.00000000
298	1455.06590000	42.95380000	0.00000000
299	1455.09320000	95.67040000	0.00000000
300	1455.18890000	22.55400000	0.00000000
301	1455.20030000	15.12220000	0.00000000
302	1461.79040000	0.00010000	0.00000000
303	1467.38060000	44.60510000	0.00000000
304	1478.19900000	0.00250000	0.00000000
305	1478.68800000	10.97950000	0.00000000
306	1479.69930000	1.05380000	0.00000000
307	1479.70570000	10.05520000	0.00000000
308	1479.72370000	10.38480000	0.00000000
309	1492.52950000	44.11670000	0.00000000
310	1505.54570000	0.02680000	0.00000000
311	1510.97340000	48.59480000	0.00000000
312	1527.24790000	14.68590000	0.00000000
313	1527.28170000	28.87470000	0.00000000
314	1528.09440000	17.48480000	0.00000000
315	1528.19860000	0.00710000	0.00000000
316	1528.24600000	14.97490000	0.00000000

317	1541.48040000	0.00000000	0.00000000
318	1546.80260000	13.09650000	0.00000000
319	1546.81130000	8.38180000	0.00000000
320	1547.29550000	3.47130000	0.00000000
321	1547.32810000	0.02140000	0.00000000
322	1578.34830000	0.03450000	0.00000000
323	1600.76150000	1.38860000	0.00000000
324	1609.79570000	0.00000000	0.00000000
325	1612.77910000	0.45010000	0.00000000
326	1612.83360000	2.66280000	0.00000000
327	1614.67430000	11.09150000	0.00000000
328	1614.83780000	0.01580000	0.00000000
329	1617.52310000	34.51530000	0.00000000
330	1618.49620000	3.54210000	0.00000000
331	1619.47120000	11.41420000	0.00000000
332	1619.54730000	0.76150000	0.00000000
333	1619.56790000	7.64230000	0.00000000
334	1619.63130000	6.05520000	0.00000000
335	1620.72380000	0.08440000	0.00000000
336	1630.04050000	0.00000000	0.00000000
337	1632.49710000	0.53830000	0.00000000
338	1633.36440000	2.64300000	0.00000000
339	1633.40230000	0.01600000	0.00000000
340	1633.64120000	2.83990000	0.00000000
341	1633.70350000	7.02580000	0.00000000
342	1640.50740000	0.01100000	0.00000000
343	1662.04560000	10.07260000	0.00000000
344	1662.07620000	10.03830000	0.00000000
345	1662.36110000	2.86720000	0.00000000
346	1662.39500000	2.50820000	0.00000000
347	3184.69540000	0.15410000	0.00000000
348	3184.69890000	0.15330000	0.00000000
349	3184.72440000	0.15190000	0.00000000
350	3184.72660000	0.15090000	0.00000000
351	3192.70370000	2.29310000	0.00000000
352	3192.70700000	2.19760000	0.00000000
353	3192.75590000	2.36580000	0.00000000
354	3192.75800000	2.15500000	0.00000000
355	3194.75710000	2.66590000	0.00000000
356	3194.77510000	2.60740000	0.00000000
357	3194.77550000	2.74990000	0.00000000
358	3194.78230000	2.78060000	0.00000000
359	3199.43110000	27.39460000	0.00000000
360	3199.45140000	27.52150000	0.00000000
361	3199.45910000	10.79680000	0.00000000
362	3199.48000000	9.86890000	0.00000000
363	3209.55500000	0.01930000	0.00000000
364	3209.60320000	1.28760000	0.00000000
365	3209.62420000	0.06140000	0.00000000
366	3209.69270000	0.00050000	0.00000000
367	3210.39970000	2.72520000	0.00000000
368	3210.40980000	1.81170000	0.00000000
369	3210.41350000	2.76860000	0.00000000
370	3210.42340000	1.79420000	0.00000000
371	3210.70130000	4.83050000	0.00000000
372	3210.70960000	0.15980000	0.00000000
373	3210.71220000	5.79010000	0.00000000
374	3210.72090000	0.20970000	0.00000000
375	3221.74750000	0.35510000	0.00000000
376	3221.78760000	8.79110000	0.00000000
377	3221.92450000	4.92780000	0.00000000
378	3221.96400000	0.02370000	0.00000000
379	3223.52850000	11.47890000	0.00000000
380	3223.53360000	11.60720000	0.00000000

381	3223.54590000	5.09530000	0.00000000
382	3223.55320000	2.41240000	0.00000000
383	3224.83540000	11.35790000	0.00000000
384	3224.88020000	12.23440000	0.00000000
385	3225.08630000	1.05850000	0.00000000
386	3225.13140000	0.69410000	0.00000000
387	3227.15230000	0.37260000	0.00000000
388	3227.24050000	11.49690000	0.00000000
389	3227.24280000	9.53910000	0.00000000
390	3227.33480000	0.00130000	0.00000000

S6. CALCULATIONS ON 1^{4+} (^{3}A) IN MeCN

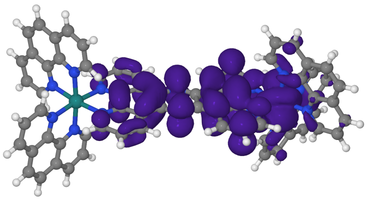

Route	: # opt freq b3lyp/genecp scrf=(solvent=acetonitrile) geom=connectivity
	: empiricaldispersion=gd3bj int=ultrafine pop=regular
SMILES	: c1cc2ccc3ccc[n+]4c3c2[n+](c1)[Ru]456([n+]7cccc8c7c9[n+]5cccc9cc8)[n+]1cccc2c1c1[n+]6cccc1c1c2nc2c3ccc[n+]4c3c3c(c2n1)ccc[n+]3[Ru]412([n+]3cccc4c3c3[n+]1cccc3cc4)[n+]1cccc3c1c1[n+]2ccccc1cc3
Formula	: $C_{72}H_{44}N_{14}Ru_2^{4+,3}$
Charge	: 4
Multiplicity	: 3
Energy	: -3727.61828967 a.u.
Gibbs Energy	: -3726.70712300 a.u.
Number of imaginary frequencies	: 2

S6.1. Cartesian Co-ordinates (XYZ format)

132

Ru	6.43764591	0.00000000	-0.00000400
N	4.84790516	0.15072000	1.32882202
N	0.00348600	0.14889701	1.40434206
N	0.00348400	-0.14876901	-1.40435100
N	4.84790087	-0.15067600	-1.32883203
C	4.89924812	0.33448601	2.64530993
H	5.88139105	0.39263600	3.09343004
C	3.73829699	0.43321100	3.42142701
H	3.82995796	0.57305199	4.48906088
C	2.49787307	0.34778601	2.80855799
H	1.58463299	0.42078200	3.38391089
C	2.40742302	0.16952400	1.42249703
C	1.14907503	0.07950300	0.71055198
C	-1.14971697	0.07108400	0.70286500
C	-3.61639810	0.06721300	0.71173102
C	-3.61639905	-0.06715000	-0.71173102
C	-2.40344691	-0.13644400	-1.42422795
C	-1.14971697	-0.07097600	-0.70287102
C	1.14907396	-0.07935200	-0.71056497
C	2.40741897	-0.16941200	-1.42250800
C	2.49786496	-0.34768701	-2.80856800
H	1.58462298	-0.42065999	-3.38392091
C	3.73828697	-0.43314600	-3.42143703
H	3.82994390	-0.57299602	-4.48906898
C	4.89924002	-0.33444801	-2.64531898
H	5.88138199	-0.39263001	-3.09343791
C	3.62592006	-0.07861100	-0.69626302
C	3.62592196	0.07869200	0.69625199
C	7.44048309	2.57650709	0.92601299
C	5.69894123	2.93824697	-0.57617098
C	7.63251877	3.95572400	1.13201594

C	8.24092579	1.62808299	1.62127399
C	5.82540417	4.32412100	-0.42175099
H	4.95769787	2.50955296	-1.23534298
C	6.78795099	4.83548403	0.42401299
C	9.25112057	2.06230402	2.50068903
H	5.16175508	4.97442484	-0.97408003
H	6.90019703	5.90479183	0.55139500
C	8.72686768	-0.61363101	2.00404000
C	10.01601505	1.06713700	3.14310193
C	9.75264168	-0.26306000	2.89074111
H	8.49632168	-1.64862502	1.79407001
H	10.80440903	1.35519099	3.82693601
H	10.32292557	-1.04814804	3.36700797
N	7.98585606	0.30763799	1.39177799
N	6.49281788	2.08900595	0.07401500
C	8.24088383	-1.62813401	-1.62127805
C	8.72688866	0.61356598	-2.00404406
C	9.25106812	-2.06238389	-2.50069094
C	7.44041204	-2.57653403	-0.92601800
C	9.75265503	0.26296601	-2.89074302
H	8.49637222	1.64856601	-1.79407501
C	10.01599121	-1.06723905	-3.14310288
C	7.63241100	-3.95575690	-1.13202000
H	10.32296181	1.04803801	-3.36700892
H	10.80437756	-1.35531402	-3.82693601
C	5.69885778	-2.93822408	0.57616401
C	6.78781796	-4.83549309	-0.42401701
C	5.82528305	-4.32410192	0.42174599
H	4.95762920	-2.50951004	1.23533702
H	6.90003490	-5.90480423	-0.55139703
H	5.16161823	-4.97438812	0.97407597
N	7.98584986	-0.30768201	-1.39178300
N	6.49275923	-2.08900690	-0.07402300
C	-4.87644196	0.24874300	2.65361404
C	-4.87644386	-0.24870400	-2.65361094
C	-2.40344596	0.13653199	1.42422497
C	-2.47769189	0.26790401	2.82385802
H	-1.56136298	0.32371700	3.39480996
C	-2.47769308	-0.26781800	-2.82386088
H	-1.56136405	-0.32361501	-3.39481497
C	-3.71459699	0.32083499	3.43270802
C	-3.71459889	-0.32077301	-3.43270898
H	-3.80700302	0.41898400	4.50588179
H	-5.85830498	0.29243499	3.10337996
H	-3.80700493	-0.41892400	-4.50588179
H	-5.85830688	-0.29241800	-3.10337496
N	-4.83259296	0.12961200	1.32649195
N	-4.83259296	-0.12957300	-1.32648897
Ru	-6.43876696	0.00000100	0.00000300
N	-7.92508698	-0.03163500	1.46681297
N	-6.55319977	-2.04756594	0.38530800
N	-7.92509222	0.03159800	-1.46680200
N	-6.55325222	2.04756403	-0.38530299
C	-8.21352005	-1.28913403	1.91451705
C	-8.60151768	0.99542201	1.97864294
C	-7.47349882	-2.36655688	1.34242702
C	-5.84040594	-3.02973509	-0.16389599
C	-8.21355915	1.28908896	-1.91450500
C	-8.60149670	-0.99547702	-1.97862995
C	-7.47356415	2.36653209	-1.34241903
C	-5.84047985	3.02975202	0.16389599
C	-9.18783092	-1.54366899	2.90056491
C	-9.58735085	0.82802403	2.96050692
H	-8.34932899	1.97684097	1.60194504

C	-7.70886278	-3.69081593	1.76374698
C	-6.00939083	-4.37177515	0.20257699
H	-5.12080383	-2.74162292	-0.91745901
C	-9.18788147	1.54359901	-2.90054893
C	-9.58733654	-0.82810497	-2.96049094
H	-8.34928131	-1.97688997	-1.60193300
C	-7.70896292	3.69078398	-1.76373804
C	-6.00950098	4.37178707	-0.20257699
H	-5.12086821	2.74165893	0.91745597
C	-9.88346672	-0.43585801	3.42600989
H	-10.10229492	1.70005703	3.33996391
C	-6.94079399	-4.70859003	1.16224003
H	-5.40243292	-5.12658882	-0.27853000
C	-9.88348770	0.43576899	-3.42599297
H	-10.10225868	-1.70015204	-3.33994699
C	-6.94091702	4.70857811	-1.16223502
H	-5.40256023	5.12661695	0.27852699
H	-10.63987923	-0.58660001	4.18613100
H	-7.08727980	-5.74007416	1.45780599
H	-7.08743095	5.74005795	-1.45780098
H	-10.63990593	0.58649099	-4.18611193
C	9.43836784	-3.47108293	-2.69005489
H	10.21429634	-3.79939294	-3.36982894
C	8.66083336	-4.37791300	-2.03729892
H	8.80646038	-5.43954277	-2.19065595
C	8.66095066	4.37785006	2.03729796
H	8.80660820	5.43947601	2.19065595
C	9.43845940	3.47099805	2.69005489
H	10.21439362	3.79928493	3.36983109
C	-8.70557594	3.92640305	-2.76806307
H	-8.88426781	4.94616508	-3.08577991
C	-9.41410923	2.89919996	-3.31089592
H	-10.16562176	3.08833909	-4.06737280
C	-8.70546532	-3.92646098	2.76807594
H	-8.88412857	-4.94622707	3.08579397
C	-9.41402245	-2.89927602	3.31091309
H	-10.16552734	-3.08843493	4.06739283

S6.2. Frequencies

Mode	IR frequency	IR intensity	Raman intensity
1	-15.22140000	0.00130000	0.00000000
2	-11.73440000	0.58170000	0.00000000
3	3.70520000	0.35720000	0.00000000
4	11.04510000	1.29850000	0.00000000
5	17.38670000	0.61370000	0.00000000
6	20.29910000	1.43300000	0.00000000
7	22.20660000	0.64460000	0.00000000
8	23.82300000	0.41170000	0.00000000
9	34.32300000	0.05570000	0.00000000
10	35.34860000	0.80990000	0.00000000
11	37.05940000	3.38620000	0.00000000
12	40.40950000	0.06590000	0.00000000
13	44.40180000	0.86500000	0.00000000
14	48.73260000	0.01970000	0.00000000
15	61.21990000	3.29250000	0.00000000
16	68.69680000	1.14600000	0.00000000
17	71.23720000	2.16500000	0.00000000
18	78.95180000	7.73490000	0.00000000
19	80.69960000	23.39270000	0.00000000
20	82.95850000	2.70160000	0.00000000
21	88.11020000	2.55230000	0.00000000
22	98.95210000	0.89450000	0.00000000
23	128.04630000	0.47990000	0.00000000
24	144.72580000	2.28750000	0.00000000
25	152.91270000	5.07370000	0.00000000
26	153.96070000	0.00030000	0.00000000
27	158.09570000	1.76990000	0.00000000
28	164.84040000	1.31590000	0.00000000
29	165.74510000	9.52730000	0.00000000
30	169.74960000	6.40990000	0.00000000
31	171.36960000	3.45790000	0.00000000
32	178.83200000	3.64440000	0.00000000
33	182.69000000	0.25350000	0.00000000
34	183.18290000	7.28540000	0.00000000
35	187.69790000	36.62410000	0.00000000
36	192.81720000	20.92960000	0.00000000
37	192.87200000	1.77430000	0.00000000
38	196.53690000	0.55740000	0.00000000
39	202.08980000	1.60410000	0.00000000
40	202.95020000	3.08900000	0.00000000
41	220.74080000	0.74230000	0.00000000
42	225.79770000	0.68550000	0.00000000
43	227.17260000	33.57300000	0.00000000
44	229.68230000	3.57860000	0.00000000
45	230.95690000	10.12010000	0.00000000
46	233.47470000	9.86150000	0.00000000
47	234.19140000	0.48270000	0.00000000
48	239.29340000	87.72570000	0.00000000
49	272.62560000	7.00720000	0.00000000
50	278.75350000	1.58610000	0.00000000
51	280.05390000	159.19690000	0.00000000
52	282.81930000	7.74310000	0.00000000
53	283.02830000	93.20500000	0.00000000
54	284.45630000	72.34230000	0.00000000
55	286.72690000	5.21000000	0.00000000
56	289.25440000	4.39170000	0.00000000
57	289.29310000	24.44960000	0.00000000
58	295.90460000	12.75270000	0.00000000
59	316.73900000	2.95570000	0.00000000
60	324.00530000	3.45740000	0.00000000

61	325.30060000	115.07950000	0.00000000
62	334.59390000	15.48000000	0.00000000
63	337.84840000	7.13410000	0.00000000
64	338.69020000	153.81670000	0.00000000
65	341.40300000	0.02070000	0.00000000
66	378.75250000	3.81140000	0.00000000
67	386.01630000	292.38010000	0.00000000
68	427.12720000	20.26610000	0.00000000
69	429.82480000	2.53700000	0.00000000
70	433.18160000	1944.24750000	0.00000000
71	437.46690000	12.29160000	0.00000000
72	438.10740000	4.75600000	0.00000000
73	441.35670000	13.50320000	0.00000000
74	443.33990000	0.49970000	0.00000000
75	443.49210000	1.70170000	0.00000000
76	444.71840000	25.61800000	0.00000000
77	445.35180000	7.10050000	0.00000000
78	446.41390000	6.35750000	0.00000000
79	446.73820000	9.47630000	0.00000000
80	450.44960000	4.99260000	0.00000000
81	463.09510000	2.02190000	0.00000000
82	466.98380000	4.69090000	0.00000000
83	469.81720000	0.97040000	0.00000000
84	474.76740000	0.84150000	0.00000000
85	474.84460000	1.03070000	0.00000000
86	479.68250000	2.36760000	0.00000000
87	490.62270000	476.58550000	0.00000000
88	501.04920000	1.64170000	0.00000000
89	502.11530000	3.33890000	0.00000000
90	504.99690000	335.89120000	0.00000000
91	506.55560000	1.04320000	0.00000000
92	520.58130000	149.36240000	0.00000000
93	520.76500000	3.33600000	0.00000000
94	521.59270000	20.23150000	0.00000000
95	521.95230000	5.74940000	0.00000000
96	523.37920000	2671.75490000	0.00000000
97	523.77290000	1.17200000	0.00000000
98	532.52580000	93.01490000	0.00000000
99	533.13670000	12.56760000	0.00000000
100	537.78330000	10.69730000	0.00000000
101	543.53270000	3.73960000	0.00000000
102	548.85610000	145.03280000	0.00000000
103	553.59710000	0.13430000	0.00000000
104	554.80450000	5.76260000	0.00000000
105	563.42440000	0.06190000	0.00000000
106	564.26750000	0.46090000	0.00000000
107	571.77030000	3.19470000	0.00000000
108	573.92070000	5.37030000	0.00000000
109	574.48180000	0.53950000	0.00000000
110	575.11820000	9.45590000	0.00000000
111	575.56220000	0.30050000	0.00000000
112	583.36660000	4.27130000	0.00000000
113	591.01970000	2.62900000	0.00000000
114	607.25230000	5.86620000	0.00000000
115	616.00870000	5869.29970000	0.00000000
116	623.33090000	1.11730000	0.00000000
117	627.97940000	51.94340000	0.00000000
118	631.53810000	0.07840000	0.00000000
119	634.44530000	0.54530000	0.00000000
120	647.53550000	1.94390000	0.00000000
121	663.02470000	1.53310000	0.00000000
122	667.10910000	11.00960000	0.00000000
123	667.39100000	3.19460000	0.00000000
124	672.85770000	9.00720000	0.00000000

125	675.02840000	32.73910000	0.00000000
126	677.49670000	2.03080000	0.00000000
127	706.60530000	77.00990000	0.00000000
128	715.39380000	77.05930000	0.00000000
129	718.77860000	0.03350000	0.00000000
130	728.90980000	2700.26560000	0.00000000
131	732.60630000	86.61360000	0.00000000
132	734.71050000	59.20740000	0.00000000
133	738.66790000	50.28120000	0.00000000
134	741.02770000	134.13010000	0.00000000
135	741.46720000	64.89410000	0.00000000
136	741.60500000	19.82600000	0.00000000
137	741.71950000	33.26430000	0.00000000
138	742.18090000	177.24970000	0.00000000
139	742.42360000	5.40250000	0.00000000
140	743.05870000	47.65080000	0.00000000
141	752.11020000	10.26090000	0.00000000
142	758.70150000	92.28550000	0.00000000
143	759.84830000	26.45230000	0.00000000
144	762.29900000	1.57720000	0.00000000
145	763.55810000	10.35680000	0.00000000
146	777.10620000	71.85720000	0.00000000
147	785.48120000	18.58630000	0.00000000
148	785.49060000	16.82220000	0.00000000
149	788.55250000	63.77900000	0.00000000
150	788.89020000	16.45560000	0.00000000
151	789.36540000	1.00240000	0.00000000
152	804.70440000	2.97330000	0.00000000
153	808.11900000	0.42520000	0.00000000
154	808.62380000	28.71410000	0.00000000
155	808.67750000	0.12900000	0.00000000
156	809.03330000	0.31190000	0.00000000
157	809.62280000	73.01320000	0.00000000
158	815.45950000	2173.29430000	0.00000000
159	826.57940000	64.13050000	0.00000000
160	827.51820000	20.25520000	0.00000000
161	844.15090000	53.88680000	0.00000000
162	849.62080000	0.50810000	0.00000000
163	851.00190000	0.27400000	0.00000000
164	851.14040000	2.17090000	0.00000000
165	852.59590000	0.12590000	0.00000000
166	862.69460000	109.81800000	0.00000000
167	862.82820000	105.72000000	0.00000000
168	864.70450000	1838.84280000	0.00000000
169	864.83350000	111.15990000	0.00000000
170	865.22660000	2453.38660000	0.00000000
171	895.78220000	1.32480000	0.00000000
172	898.38530000	21.26110000	0.00000000
173	898.48070000	69.51830000	0.00000000
174	899.97690000	22.76530000	0.00000000
175	932.25920000	6.63770000	0.00000000
176	934.63810000	21.51060000	0.00000000
177	938.26180000	2.43910000	0.00000000
178	941.34580000	593.91820000	0.00000000
179	944.00620000	1.92030000	0.00000000
180	952.49650000	26441.08120000	0.00000000
181	963.62750000	95.05120000	0.00000000
182	964.58700000	0.01310000	0.00000000
183	965.05520000	0.28120000	0.00000000
184	965.59460000	56.97450000	0.00000000
185	970.15540000	0.16800000	0.00000000
186	970.54570000	0.50030000	0.00000000
187	970.80880000	0.21320000	0.00000000
188	970.94600000	0.17520000	0.00000000

189	972.12740000	253.86780000	0.00000000
190	972.39750000	0.36400000	0.00000000
191	976.54170000	1.33340000	0.00000000
192	976.86660000	0.92890000	0.00000000
193	999.16530000	0.00290000	0.00000000
194	999.22750000	0.09360000	0.00000000
195	1003.94100000	0.01180000	0.00000000
196	1003.96660000	0.04650000	0.00000000
197	1007.33780000	26.40930000	0.00000000
198	1011.64980000	0.66890000	0.00000000
199	1012.26350000	4.81100000	0.00000000
200	1013.62480000	0.68440000	0.00000000
201	1013.75840000	1.10890000	0.00000000
202	1015.64500000	0.62670000	0.00000000
203	1016.45970000	0.00560000	0.00000000
204	1016.58570000	0.01850000	0.00000000
205	1016.66950000	0.15110000	0.00000000
206	1022.67320000	0.52700000	0.00000000
207	1022.70850000	0.21970000	0.00000000
208	1025.47700000	0.31170000	0.00000000
209	1025.50130000	0.09340000	0.00000000
210	1044.09830000	43.12660000	0.00000000
211	1061.09130000	1.94770000	0.00000000
212	1061.33310000	1.45150000	0.00000000
213	1062.34180000	172.60350000	0.00000000
214	1062.74620000	84.87240000	0.00000000
215	1064.71940000	3.91440000	0.00000000
216	1067.79510000	3020.42670000	0.00000000
217	1074.99980000	39.19440000	0.00000000
218	1083.29520000	2.36920000	0.00000000
219	1084.46130000	7.13500000	0.00000000
220	1086.89110000	25.75420000	0.00000000
221	1088.58530000	23.99260000	0.00000000
222	1089.10480000	2.58060000	0.00000000
223	1097.52560000	2760.14540000	0.00000000
224	1105.62490000	15.68610000	0.00000000
225	1114.60320000	14.70750000	0.00000000
226	1116.67100000	5.69580000	0.00000000
227	1118.26720000	18.58230000	0.00000000
228	1121.16640000	0.50480000	0.00000000
229	1122.05810000	7.67320000	0.00000000
230	1125.33850000	0.01400000	0.00000000
231	1128.76850000	352.15650000	0.00000000
232	1131.81660000	52.07800000	0.00000000
233	1134.17090000	1242.54020000	0.00000000
234	1134.92870000	16.83180000	0.00000000
235	1145.99620000	255.33440000	0.00000000
236	1153.27410000	10.22360000	0.00000000
237	1165.19900000	17.39120000	0.00000000
238	1168.63120000	11.02840000	0.00000000
239	1168.93610000	5.35270000	0.00000000
240	1171.96610000	1.34770000	0.00000000
241	1172.12680000	0.33570000	0.00000000
242	1173.75990000	16.08420000	0.00000000
243	1174.17290000	10.56850000	0.00000000
244	1176.42370000	8.11950000	0.00000000
245	1177.43640000	7.50700000	0.00000000
246	1177.45940000	1.67300000	0.00000000
247	1202.15230000	1.86760000	0.00000000
248	1209.46890000	161.51930000	0.00000000
249	1230.89540000	11.02520000	0.00000000
250	1233.20700000	63.87990000	0.00000000
251	1234.31930000	3.02770000	0.00000000
252	1235.04220000	19.68520000	0.00000000

253	1237.71160000	6.13630000	0.00000000
254	1239.86750000	125.03930000	0.00000000
255	1240.67190000	1.03910000	0.00000000
256	1241.69760000	1048.28510000	0.00000000
257	1242.54180000	3325.47600000	0.00000000
258	1250.07350000	2.27880000	0.00000000
259	1250.33920000	3.45300000	0.00000000
260	1253.59580000	12.80320000	0.00000000
261	1253.70140000	36.18050000	0.00000000
262	1255.02710000	9.69330000	0.00000000
263	1285.63580000	0.24300000	0.00000000
264	1286.72990000	1.00480000	0.00000000
265	1287.96110000	2.19450000	0.00000000
266	1288.62110000	3.52770000	0.00000000
267	1301.77190000	1166.36890000	0.00000000
268	1309.77900000	131.11710000	0.00000000
269	1317.62050000	4.82580000	0.00000000
270	1329.51230000	25.46300000	0.00000000
271	1333.24870000	2.76410000	0.00000000
272	1333.67220000	1.23030000	0.00000000
273	1342.66120000	8.68230000	0.00000000
274	1344.39010000	13.11460000	0.00000000
275	1344.81420000	6.65850000	0.00000000
276	1347.85800000	5.65660000	0.00000000
277	1347.86220000	4.19920000	0.00000000
278	1348.38170000	1.03550000	0.00000000
279	1348.99970000	6.23030000	0.00000000
280	1349.82200000	2.47230000	0.00000000
281	1361.96220000	361.23680000	0.00000000
282	1362.23550000	10.80410000	0.00000000
283	1368.79220000	498.34830000	0.00000000
284	1372.87550000	9.32470000	0.00000000
285	1373.68010000	2.03790000	0.00000000
286	1377.10300000	17.84450000	0.00000000
287	1379.79080000	10.37000000	0.00000000
288	1394.34170000	84.54610000	0.00000000
289	1408.82700000	829.43970000	0.00000000
290	1410.98290000	71.09350000	0.00000000
291	1442.09770000	38.74590000	0.00000000
292	1443.25790000	2.92880000	0.00000000
293	1443.47290000	32.13060000	0.00000000
294	1445.91260000	24.57160000	0.00000000
295	1446.17490000	1154.13100000	0.00000000
296	1447.20660000	547.13610000	0.00000000
297	1453.21260000	35.95400000	0.00000000
298	1453.44960000	4.73090000	0.00000000
299	1457.20550000	36.97010000	0.00000000
300	1457.22620000	1.75510000	0.00000000
301	1458.12220000	86.11220000	0.00000000
302	1458.49550000	27.33530000	0.00000000
303	1461.37130000	28.38900000	0.00000000
304	1462.09930000	119.57360000	0.00000000
305	1463.84400000	56.56110000	0.00000000
306	1480.19680000	69.19220000	0.00000000
307	1481.92860000	13.54710000	0.00000000
308	1482.78560000	9.01480000	0.00000000
309	1486.03820000	5.42090000	0.00000000
310	1486.55300000	8.38480000	0.00000000
311	1495.78040000	1027.42220000	0.00000000
312	1508.24960000	21.73030000	0.00000000
313	1516.35320000	148.04570000	0.00000000
314	1528.57420000	24.82680000	0.00000000
315	1528.60490000	19.47320000	0.00000000
316	1529.29890000	4.34230000	0.00000000

317	1529.63350000	17.98650000	0.00000000
318	1531.48350000	5.40780000	0.00000000
319	1548.97310000	2.83230000	0.00000000
320	1549.89430000	0.04230000	0.00000000
321	1555.96370000	165.31730000	0.00000000
322	1556.51580000	63.89180000	0.00000000
323	1559.30140000	160.29360000	0.00000000
324	1569.54570000	148.01140000	0.00000000
325	1584.08210000	800.48270000	0.00000000
326	1607.74240000	37.18560000	0.00000000
327	1608.11650000	95.30080000	0.00000000
328	1611.16210000	24.16770000	0.00000000
329	1616.30120000	60.98860000	0.00000000
330	1618.19000000	56.15660000	0.00000000
331	1618.78070000	3.84900000	0.00000000
332	1619.62550000	6.08970000	0.00000000
333	1619.77820000	1.96280000	0.00000000
334	1620.35250000	30.29670000	0.00000000
335	1620.88460000	36.42450000	0.00000000
336	1620.97210000	31.86010000	0.00000000
337	1621.90770000	4.04060000	0.00000000
338	1630.02990000	208.66780000	0.00000000
339	1634.56990000	3.35290000	0.00000000
340	1634.93460000	5.72530000	0.00000000
341	1635.11690000	0.07880000	0.00000000
342	1635.25010000	21.18990000	0.00000000
343	1664.24420000	8.12640000	0.00000000
344	1664.61220000	2.18680000	0.00000000
345	1665.39140000	21.83880000	0.00000000
346	1665.60280000	2.80320000	0.00000000
347	3186.29230000	0.43690000	0.00000000
348	3186.29250000	0.07750000	0.00000000
349	3193.83530000	2.80640000	0.00000000
350	3193.83650000	0.27040000	0.00000000
351	3193.97380000	0.01150000	0.00000000
352	3193.97400000	0.05620000	0.00000000
353	3196.27030000	1.85130000	0.00000000
354	3196.27810000	1.90740000	0.00000000
355	3200.98550000	32.19640000	0.00000000
356	3201.01710000	14.44550000	0.00000000
357	3201.58760000	2.28170000	0.00000000
358	3201.58960000	2.04340000	0.00000000
359	3202.35860000	5.00300000	0.00000000
360	3202.36650000	0.18650000	0.00000000
361	3205.34060000	5.85590000	0.00000000
362	3205.35760000	0.44340000	0.00000000
363	3207.71120000	143.56010000	0.00000000
364	3207.97650000	12.74590000	0.00000000
365	3207.99630000	8.55730000	0.00000000
366	3208.21430000	2.84040000	0.00000000
367	3211.05180000	2.93280000	0.00000000
368	3211.07570000	1.75620000	0.00000000
369	3211.38750000	6.17660000	0.00000000
370	3211.40710000	0.34180000	0.00000000
371	3213.41310000	0.68660000	0.00000000
372	3213.42360000	15.71120000	0.00000000
373	3216.66690000	0.78470000	0.00000000
374	3216.70220000	0.10060000	0.00000000
375	3217.56910000	0.10950000	0.00000000
376	3217.65000000	0.72590000	0.00000000
377	3219.64930000	0.68680000	0.00000000
378	3219.70880000	5.40730000	0.00000000
379	3222.98450000	11.96620000	0.00000000
380	3222.99050000	5.91960000	0.00000000

381	3224.72220000	8.75070000	0.00000000
382	3224.84770000	5.13740000	0.00000000
383	3225.02660000	17.52560000	0.00000000
384	3225.25780000	0.54360000	0.00000000
385	3225.90170000	7.21160000	0.00000000
386	3225.96640000	5.24720000	0.00000000
387	3227.59950000	3.15000000	0.00000000
388	3227.63080000	8.92990000	0.00000000
389	3228.28990000	6.95540000	0.00000000
390	3228.49790000	2.90370000	0.00000000

S7. CALCULATIONS ON 2^{4+} (^{1}A) IN MeCN

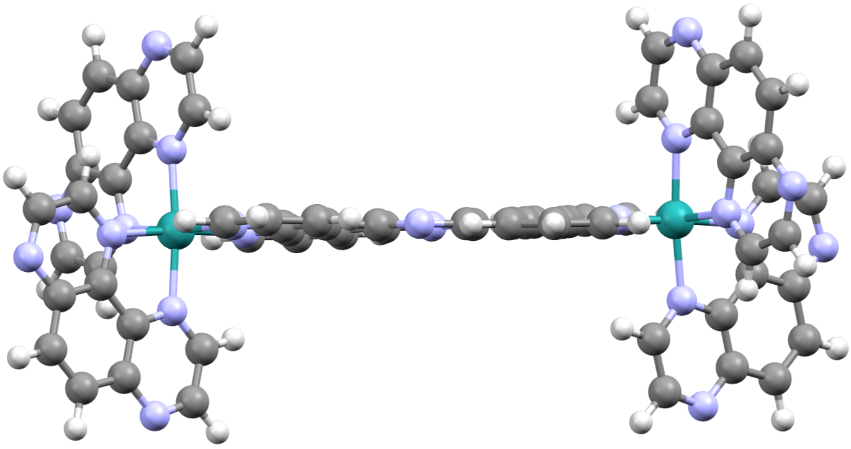

Route	: # opt freq b3lyp/genecp scrf=(solvent=acetonitrile) geom=connectivity	
	: empiricaldispersion=gd3bj int=ultrafine pop=regular	
SMILES	: c1cc2c3c(c4ccc[n+]5c4c2[n+](c1)[Ru]567([n+]8ccnc9c8c1[n+]6ccnc1cc9)[n+]1ccnc2c1c1[n+]7ccnc1cc2)nc1c2ccc[n+]4c2c2c(c1n3)ccc[n+]2[Ru]412([n+]3ccnc4c3c3[n+]1ccnc3cc4)[n+]1ccnc3c1c1[n+]2ccnc1cc3	
Formula	: $C_{64}H_{36}N_{22}Ru_2^{4+}$	
Charge	: 4	
Multiplicity	: 1	
Energy	: -3855.92224006	a.u.
Gibbs Energy	: -3855.09917600	a.u.
Number of imaginary frequencies	: 1	

S7.1. Cartesian Co-ordinates (XYZ format)

124

Ru	-6.42851686	-0.00000300	-0.00001500
N	-4.81623602	-0.06660500	-1.33004606
N	-0.00000300	-0.00001800	-1.39570904
N	-0.00000800	-0.00025400	1.39570904
N	-4.81623888	0.06649500	1.33002603
C	-4.86450291	-0.11472600	-2.66459298
H	-5.84633589	-0.14699100	-3.11409092
C	-3.71109605	-0.12844600	-3.45091796
H	-3.80778790	-0.16880600	-4.52687597
C	-2.47046208	-0.09181800	-2.84361291
H	-1.55614305	-0.10311600	-3.42039108
C	-2.40106606	-0.04148200	-1.44385397
C	-1.14257002	-0.00929500	-0.71174401
C	1.14256203	0.00914900	-0.71173799
C	3.60060096	0.02712300	-0.72063202
C	3.60059810	-0.02729600	0.72064698
C	2.40105605	-0.04172300	1.44384694
C	1.14255905	-0.00941600	0.71174198

C	-1.14257205	0.00902800	0.71173900
C	-2.40107107	0.04125600	1.44384503
C	-2.47047091	0.09159600	2.84360290
H	-1.55615401	0.10285600	3.42038488
C	-3.71110606	0.12827800	3.45090294
H	-3.80780101	0.16864499	4.52686024
C	-4.86451006	0.11461600	2.66457200
H	-5.84634304	0.14693300	3.11406493
C	-3.60061002	0.02700600	0.72063702
C	-3.60060811	-0.02717900	-0.72065097
C	-7.43644810	2.42020392	-1.22885597
C	-5.85082197	3.04681301	0.30356601
C	-7.65084124	3.76316905	-1.58826602
C	-8.17425919	1.38370299	-1.85246694
C	-6.07234716	4.38870621	-0.07101800
H	-5.13056183	2.78723907	1.06567895
C	-9.13587666	1.68479300	-2.83417797
H	-5.50071096	5.17024279	0.41603300
C	-8.62268925	-0.85556799	-2.05048490
C	-9.58779907	-0.53836298	-3.02946091
H	-8.43495274	-1.87899494	-1.75909805
H	-10.15091991	-1.34166098	-3.49024892
N	-7.91292906	0.10294100	-1.46604896
N	-6.53213978	2.06381893	-0.27320901
C	-8.17436314	-1.38358998	1.85242701
C	-8.62263584	0.85571301	2.05044794
C	-9.13600731	-1.68461502	2.83413291
C	-7.43662119	-2.42014194	1.22881997
C	-9.58777237	0.53857303	3.02942109
H	-8.43482685	1.87912703	1.75906503
C	-7.65111017	-3.76309204	1.58822596
H	-10.15083694	1.34190905	3.49020910
C	-5.85102892	-3.04685903	-0.30359301
C	-6.07264996	-4.38873816	0.07098700
H	-5.13074589	-2.78733492	-1.06570196
H	-5.50106478	-5.17031384	-0.41606200
N	-7.91294193	-0.10284500	1.46601295
N	-6.53228092	-2.06381798	0.27317899
C	4.86449814	0.11504300	-2.66455507
C	4.86449099	-0.11515300	2.66457605
C	2.40106106	0.04149600	-1.44383800
C	2.47045898	0.09206500	-2.84358811
H	1.55614102	0.10342300	-3.42036700
C	2.47044992	-0.09228700	2.84359789
H	1.55613005	-0.10368300	3.42037296
C	3.71109295	0.12884000	-3.45088291
C	3.71108294	-0.12900700	3.45089793
H	3.80778694	0.16937999	-4.52683306
H	5.84633017	0.14742400	-3.11404610
H	3.80777407	-0.16954100	4.52684879
H	5.84632301	-0.14748199	3.11407089
N	4.81622791	0.06670500	-1.33001697
N	4.81622601	-0.06681600	1.33003700
Ru	6.42850494	-0.00000300	0.00001500
N	7.91293192	-0.10262800	-1.46603000
N	6.53227377	-2.06377697	-0.27348399
N	7.91292000	0.10272300	1.46606696
N	6.53213310	2.06377697	0.27351400
C	8.17437458	-1.38331699	-1.85261202
C	8.62260723	0.85601801	-2.05034494
C	7.43663597	-2.41996193	-1.22915399
C	5.85101223	-3.04690099	0.30313399
C	8.17427063	1.38343000	1.85265100
C	8.62265968	-0.85587400	2.05038095

C	7.43646383	2.42002392	1.22919095
C	5.85080481	3.04685497	-0.30310699
C	9.13602829	-1.68419695	-2.83435202
C	9.58774471	0.53902298	-3.02936101
H	8.43478203	1.87939095	-1.75882602
C	7.65114403	-3.76286101	-1.58873904
C	6.07265186	-4.38872719	-0.07162300
H	5.13070583	-2.78748202	1.06525803
C	9.13589764	1.68437600	2.83439708
C	9.58777332	-0.53881299	3.02940106
H	8.43490887	-1.87925899	1.75885904
C	7.65087605	3.76293802	1.58877897
C	6.07235003	4.38869476	0.07165400
H	5.13052177	2.78738594	-1.06523502
H	10.15079117	1.34243095	-3.49005008
H	5.50106096	-5.17037392	0.41530401
H	10.15087509	-1.34218204	3.49008989
H	5.50070715	5.17030287	-0.41527501
C	-9.34327030	-3.05850792	3.19172502
H	-10.08540344	-3.27068090	3.94998908
C	-8.63014412	-4.05483580	2.59564996
H	-8.78080463	-5.09326315	2.85971498
C	-8.62984943	4.05497885	-2.59569693
H	-8.78043556	5.09341621	-2.85976410
C	-9.34304142	3.05870008	-3.19177294
H	-10.08515739	3.27092409	-3.95004201
C	8.62990475	4.05460215	2.59623194
H	8.78050995	5.09300089	2.86043596
C	9.34308910	3.05823302	3.19216895
H	10.08521461	3.27034497	3.95045805
C	8.63020039	-4.05445814	-2.59618497
H	8.78087902	-5.09284782	-2.86038709
C	9.34331703	-3.05804110	-3.19212008
H	10.08546162	-3.27010202	-3.95040607
N	6.94741917	-4.75382519	-0.99184197
N	9.84696007	-0.69454098	-3.42464089
N	-6.94738913	-4.75396919	0.99117899
N	-9.84695816	-0.69504702	3.42454696
N	-9.84689617	0.69527400	-3.42459202
N	-6.94705486	4.75399685	-0.99121600
N	9.84689808	0.69476801	3.42468596
N	6.94708490	4.75385284	0.99187899

S7.2. Frequencies

Mode	IR frequency	IR intensity	Raman intensity
1	-6.60470000	0.00000000	0.00000000
2	4.71870000	0.19880000	0.00000000
3	12.78580000	0.00620000	0.00000000
4	23.97530000	0.00000000	0.00000000
5	24.83720000	0.46440000	0.00000000
6	27.23190000	0.56240000	0.00000000
7	29.42230000	0.09500000	0.00000000
8	36.41680000	0.02930000	0.00000000
9	39.63940000	0.00100000	0.00000000
10	44.74030000	0.00000000	0.00000000
11	46.10000000	0.00300000	0.00000000
12	46.23180000	0.37290000	0.00000000
13	51.05570000	0.11480000	0.00000000
14	54.28470000	0.00000000	0.00000000
15	63.95890000	0.39590000	0.00000000
16	72.82450000	1.30350000	0.00000000
17	79.28840000	0.00000000	0.00000000
18	86.40960000	9.49110000	0.00000000
19	91.76240000	3.06390000	0.00000000
20	92.78340000	0.01190000	0.00000000
21	97.23830000	0.96980000	0.00000000
22	105.61270000	0.00000000	0.00000000
23	134.94690000	0.00660000	0.00000000
24	147.05470000	0.20990000	0.00000000
25	159.17370000	0.07460000	0.00000000
26	167.57940000	4.57720000	0.00000000
27	170.75580000	1.20660000	0.00000000
28	171.41070000	0.32980000	0.00000000
29	174.98710000	0.00000000	0.00000000
30	177.20550000	0.31500000	0.00000000
31	178.20540000	0.00000000	0.00000000
32	183.12190000	0.71160000	0.00000000
33	186.95670000	0.07550000	0.00000000
34	187.48980000	0.01610000	0.00000000
35	188.94930000	0.00000000	0.00000000
36	193.30620000	0.09740000	0.00000000
37	195.14060000	0.02570000	0.00000000
38	197.00780000	2.80240000	0.00000000
39	206.59600000	0.00000000	0.00000000
40	211.90720000	0.55670000	0.00000000
41	224.22400000	0.00070000	0.00000000
42	224.65720000	24.04920000	0.00000000
43	226.17570000	0.35350000	0.00000000
44	226.72490000	26.42390000	0.00000000
45	231.95480000	0.00110000	0.00000000
46	241.08040000	0.17710000	0.00000000
47	245.37610000	0.00000000	0.00000000
48	251.65010000	0.72970000	0.00000000
49	286.11140000	26.55940000	0.00000000
50	286.20050000	6.54130000	0.00000000
51	286.54440000	6.53250000	0.00000000
52	286.59430000	0.00020000	0.00000000
53	293.94230000	2.75730000	0.00000000
54	294.29400000	1.92870000	0.00000000
55	297.38620000	2.36390000	0.00000000
56	297.40530000	0.00390000	0.00000000
57	304.69720000	0.00000000	0.00000000
58	319.41320000	4.20960000	0.00000000
59	319.81820000	19.94150000	0.00000000
60	325.40320000	0.00660000	0.00000000

61	337.27790000	0.00000000	0.00000000
62	342.90840000	1.36530000	0.00000000
63	350.21560000	3.25540000	0.00000000
64	351.75030000	1.02430000	0.00000000
65	352.40520000	0.05770000	0.00000000
66	388.81230000	0.00000000	0.00000000
67	393.98720000	0.52260000	0.00000000
68	440.97420000	17.47140000	0.00000000
69	444.40900000	0.00000000	0.00000000
70	447.03080000	4.68540000	0.00000000
71	447.09170000	0.04280000	0.00000000
72	447.19030000	0.04110000	0.00000000
73	447.56040000	0.04170000	0.00000000
74	447.63800000	0.00000000	0.00000000
75	452.33820000	0.01560000	0.00000000
76	456.71640000	5.13850000	0.00000000
77	459.35250000	14.66610000	0.00000000
78	460.06670000	0.00000000	0.00000000
79	460.49210000	0.80920000	0.00000000
80	468.61380000	2.15870000	0.00000000
81	471.38830000	1.01730000	0.00000000
82	479.90230000	9.78840000	0.00000000
83	484.32230000	0.00000000	0.00000000
84	485.24330000	36.08350000	0.00000000
85	486.16570000	20.48840000	0.00000000
86	488.28640000	1.06120000	0.00000000
87	494.09340000	0.00000000	0.00000000
88	498.79760000	12.47200000	0.00000000
89	503.54190000	2.65240000	0.00000000
90	504.48020000	24.27140000	0.00000000
91	505.60930000	0.00000000	0.00000000
92	536.85540000	17.69100000	0.00000000
93	546.69110000	0.34230000	0.00000000
94	552.94220000	0.16570000	0.00000000
95	555.20200000	12.23400000	0.00000000
96	555.31170000	8.23220000	0.00000000
97	555.34100000	0.00230000	0.00000000
98	555.42240000	9.83050000	0.00000000
99	555.57310000	7.64420000	0.00000000
100	561.71220000	0.00000000	0.00000000
101	562.00940000	0.00400000	0.00000000
102	570.83500000	0.00000000	0.00000000
103	575.17110000	0.15460000	0.00000000
104	582.07380000	50.11360000	0.00000000
105	582.10200000	28.02560000	0.00000000
106	583.94590000	0.00160000	0.00000000
107	584.01350000	22.73050000	0.00000000
108	592.07550000	0.55430000	0.00000000
109	593.15260000	4.66150000	0.00000000
110	594.59860000	0.00010000	0.00000000
111	594.60280000	0.00000000	0.00000000
112	595.46700000	14.59110000	0.00000000
113	595.67110000	0.91090000	0.00000000
114	620.77490000	0.07230000	0.00000000
115	640.10860000	0.12060000	0.00000000
116	640.80660000	0.66760000	0.00000000
117	644.03300000	0.00000000	0.00000000
118	644.07620000	0.13510000	0.00000000
119	661.55480000	0.00000000	0.00000000
120	663.05090000	1.68540000	0.00000000
121	665.16910000	0.50280000	0.00000000
122	665.98310000	3.46860000	0.00000000
123	670.63830000	8.22670000	0.00000000
124	671.03700000	0.01790000	0.00000000

125	671.06630000	0.00000000	0.00000000
126	675.07460000	0.01770000	0.00000000
127	718.71510000	0.01310000	0.00000000
128	727.26680000	0.00000000	0.00000000
129	743.33050000	0.00000000	0.00000000
130	746.63090000	33.29450000	0.00000000
131	749.62840000	33.87860000	0.00000000
132	749.80460000	54.68690000	0.00000000
133	752.58390000	91.20400000	0.00000000
134	753.75660000	0.00050000	0.00000000
135	753.85420000	0.37080000	0.00000000
136	753.86440000	0.00040000	0.00000000
137	754.06380000	33.41960000	0.00000000
138	756.48320000	0.00540000	0.00000000
139	759.03780000	0.00000000	0.00000000
140	759.54540000	11.18500000	0.00000000
141	761.01180000	35.28520000	0.00000000
142	761.96970000	1.05550000	0.00000000
143	763.42580000	21.07800000	0.00000000
144	763.45870000	0.00000000	0.00000000
145	764.46700000	0.81130000	0.00000000
146	812.23620000	0.00150000	0.00000000
147	819.70640000	0.00000000	0.00000000
148	825.01130000	0.19150000	0.00000000
149	825.99460000	0.00000000	0.00000000
150	835.45410000	130.34330000	0.00000000
151	839.99890000	0.27150000	0.00000000
152	854.23420000	0.26710000	0.00000000
153	854.24250000	0.24410000	0.00000000
154	854.49880000	0.00300000	0.00000000
155	854.54090000	0.24330000	0.00000000
156	856.77090000	0.00000000	0.00000000
157	859.83250000	0.07900000	0.00000000
158	859.84390000	0.36310000	0.00000000
159	860.76480000	0.20590000	0.00000000
160	861.12160000	0.00000000	0.00000000
161	873.26770000	17.25350000	0.00000000
162	877.20680000	0.41110000	0.00000000
163	882.87500000	3.37700000	0.00000000
164	882.89510000	1.34960000	0.00000000
165	883.35850000	0.05720000	0.00000000
166	883.45140000	7.25150000	0.00000000
167	894.32200000	2.55630000	0.00000000
168	894.34040000	196.45230000	0.00000000
169	894.60590000	195.90370000	0.00000000
170	894.61540000	0.30260000	0.00000000
171	925.52940000	0.07050000	0.00000000
172	925.55080000	0.34850000	0.00000000
173	927.54580000	0.00010000	0.00000000
174	927.58520000	1.00780000	0.00000000
175	945.88870000	0.00040000	0.00000000
176	946.53260000	0.17840000	0.00000000
177	948.44990000	0.00060000	0.00000000
178	948.47180000	1.93350000	0.00000000
179	949.20740000	0.54770000	0.00000000
180	985.40190000	0.04610000	0.00000000
181	985.43840000	0.03080000	0.00000000
182	986.34470000	0.00000000	0.00000000
183	986.76950000	0.01080000	0.00000000
184	998.84780000	0.18700000	0.00000000
185	998.86750000	0.16000000	0.00000000
186	998.86950000	0.13670000	0.00000000
187	998.88990000	0.10500000	0.00000000
188	1001.91440000	0.15920000	0.00000000

189	1001.92150000	0.15860000	0.00000000
190	1002.01390000	0.07710000	0.00000000
191	1002.02040000	0.09150000	0.00000000
192	1016.42000000	0.00420000	0.00000000
193	1016.42090000	0.00400000	0.00000000
194	1016.42130000	0.01100000	0.00000000
195	1016.42220000	0.01120000	0.00000000
196	1028.47790000	0.70760000	0.00000000
197	1029.13830000	0.01200000	0.00000000
198	1029.91630000	0.05810000	0.00000000
199	1030.47470000	0.00000000	0.00000000
200	1049.94560000	0.45730000	0.00000000
201	1050.48220000	0.00110000	0.00000000
202	1052.00220000	0.00000000	0.00000000
203	1052.13800000	3.99510000	0.00000000
204	1052.72900000	2.15220000	0.00000000
205	1063.18190000	0.00000000	0.00000000
206	1067.59370000	0.42610000	0.00000000
207	1071.09310000	0.66630000	0.00000000
208	1078.31820000	6.92640000	0.00000000
209	1078.33300000	7.67960000	0.00000000
210	1079.03690000	6.55910000	0.00000000
211	1079.10460000	0.00130000	0.00000000
212	1088.01010000	38.42210000	0.00000000
213	1103.69580000	0.00000000	0.00000000
214	1121.57980000	11.19000000	0.00000000
215	1122.46790000	5.93900000	0.00000000
216	1122.57400000	4.10510000	0.00000000
217	1124.44730000	60.61520000	0.00000000
218	1124.50690000	42.53710000	0.00000000
219	1124.84890000	0.00010000	0.00000000
220	1125.85230000	6.91760000	0.00000000
221	1126.62320000	0.00120000	0.00000000
222	1126.82510000	67.46170000	0.00000000
223	1127.12480000	2.43290000	0.00000000
224	1130.89130000	9.67810000	0.00000000
225	1151.74740000	0.00000000	0.00000000
226	1159.18520000	192.89770000	0.00000000
227	1161.84470000	19.90660000	0.00000000
228	1162.56650000	0.11950000	0.00000000
229	1183.75190000	3.66580000	0.00000000
230	1183.75450000	3.70110000	0.00000000
231	1186.09230000	0.40870000	0.00000000
232	1186.14110000	0.00030000	0.00000000
233	1211.70380000	0.99730000	0.00000000
234	1226.88410000	0.00000000	0.00000000
235	1230.49340000	2.17070000	0.00000000
236	1233.55140000	6.80300000	0.00000000
237	1233.56730000	2.65210000	0.00000000
238	1234.99640000	2.91620000	0.00000000
239	1235.03790000	0.00000000	0.00000000
240	1250.48460000	10.65600000	0.00000000
241	1250.48770000	3.28380000	0.00000000
242	1251.03260000	30.92520000	0.00000000
243	1251.09520000	10.65360000	0.00000000
244	1264.19000000	5.87360000	0.00000000
245	1264.35320000	0.91580000	0.00000000
246	1264.42700000	2.87950000	0.00000000
247	1264.43440000	0.19020000	0.00000000
248	1265.98240000	0.02640000	0.00000000
249	1284.93050000	19.60950000	0.00000000
250	1306.03690000	0.00000000	0.00000000
251	1309.56400000	47.96300000	0.00000000
252	1309.57200000	239.84110000	0.00000000

253	1309.93920000	111.38370000	0.00000000
254	1309.95250000	0.01360000	0.00000000
255	1312.60640000	35.20740000	0.00000000
256	1312.72410000	69.95180000	0.00000000
257	1314.69280000	21.05210000	0.00000000
258	1314.69520000	1.91200000	0.00000000
259	1315.08660000	1.74090000	0.00000000
260	1322.73400000	45.01000000	0.00000000
261	1323.44750000	3.62080000	0.00000000
262	1333.51700000	0.07620000	0.00000000
263	1336.58620000	0.00410000	0.00000000
264	1353.07840000	0.00000000	0.00000000
265	1360.31990000	29.26290000	0.00000000
266	1372.56090000	0.26980000	0.00000000
267	1372.56740000	0.17540000	0.00000000
268	1373.10910000	0.56190000	0.00000000
269	1373.11510000	0.26080000	0.00000000
270	1389.80640000	339.52570000	0.00000000
271	1394.97070000	0.00000000	0.00000000
272	1413.60330000	54.76920000	0.00000000
273	1413.60760000	3.82130000	0.00000000
274	1413.69120000	53.18280000	0.00000000
275	1413.69700000	135.10490000	0.00000000
276	1428.62940000	42.19220000	0.00000000
277	1428.63250000	0.07240000	0.00000000
278	1428.75640000	57.75610000	0.00000000
279	1428.75820000	93.98500000	0.00000000
280	1435.37310000	180.56820000	0.00000000
281	1443.74970000	130.79750000	0.00000000
282	1443.83130000	60.46030000	0.00000000
283	1445.23530000	42.51020000	0.00000000
284	1445.24900000	1.34380000	0.00000000
285	1458.17780000	0.66310000	0.00000000
286	1469.88000000	0.00000000	0.00000000
287	1473.78360000	40.67120000	0.00000000
288	1487.65390000	0.00000000	0.00000000
289	1490.77300000	1.96150000	0.00000000
290	1490.87230000	0.00020000	0.00000000
291	1490.88660000	2.81190000	0.00000000
292	1490.94770000	11.26330000	0.00000000
293	1501.21780000	37.91360000	0.00000000
294	1510.34580000	0.71170000	0.00000000
295	1521.48800000	104.21250000	0.00000000
296	1521.49150000	252.86860000	0.00000000
297	1521.75150000	179.92100000	0.00000000
298	1521.80180000	0.00330000	0.00000000
299	1523.61510000	65.98040000	0.00000000
300	1534.96750000	16.59060000	0.00000000
301	1535.44520000	144.84240000	0.00000000
302	1536.27250000	83.40370000	0.00000000
303	1539.91180000	80.96590000	0.00000000
304	1539.98270000	0.08710000	0.00000000
305	1548.80420000	0.00000000	0.00000000
306	1582.95390000	7.81620000	0.00000000
307	1582.95780000	0.07090000	0.00000000
308	1582.96550000	1.98880000	0.00000000
309	1582.96750000	12.29300000	0.00000000
310	1584.02570000	5.52140000	0.00000000
311	1585.40080000	33.47000000	0.00000000
312	1585.45280000	109.17900000	0.00000000
313	1585.95690000	43.46980000	0.00000000
314	1585.96960000	0.00010000	0.00000000
315	1606.17540000	0.85920000	0.00000000
316	1614.70090000	0.00000000	0.00000000

317	1614.81260000	12.04080000	0.00000000
318	1614.85350000	29.81110000	0.00000000
319	1616.72170000	3.69900000	0.00000000
320	1616.94720000	0.00000000	0.00000000
321	1622.93400000	39.35100000	0.00000000
322	1624.26520000	6.81250000	0.00000000
323	1626.63670000	0.00820000	0.00000000
324	1634.74840000	0.00000000	0.00000000
325	1640.40890000	0.00870000	0.00000000
326	1648.78650000	5.03740000	0.00000000
327	1655.54780000	19.99180000	0.00000000
328	1655.55150000	11.21770000	0.00000000
329	1656.75770000	12.38530000	0.00000000
330	1656.77300000	0.00180000	0.00000000
331	3186.98740000	12.09990000	0.00000000
332	3186.98950000	18.03020000	0.00000000
333	3187.02480000	11.43060000	0.00000000
334	3187.02660000	2.76940000	0.00000000
335	3188.95310000	14.06630000	0.00000000
336	3188.95830000	16.77940000	0.00000000
337	3188.99440000	5.22510000	0.00000000
338	3188.99960000	3.67570000	0.00000000
339	3205.02020000	0.27360000	0.00000000
340	3205.02040000	0.12530000	0.00000000
341	3205.02110000	0.26490000	0.00000000
342	3205.02140000	0.12510000	0.00000000
343	3214.66160000	0.06560000	0.00000000
344	3214.72790000	0.24120000	0.00000000
345	3214.82460000	0.02030000	0.00000000
346	3214.90310000	0.00000000	0.00000000
347	3217.11620000	5.71500000	0.00000000
348	3217.11720000	5.83200000	0.00000000
349	3217.13110000	2.84930000	0.00000000
350	3217.13210000	1.03920000	0.00000000
351	3224.56560000	0.23030000	0.00000000
352	3224.59990000	11.20010000	0.00000000
353	3225.01980000	3.84120000	0.00000000
354	3225.05790000	0.00010000	0.00000000
355	3229.57890000	3.78980000	0.00000000
356	3229.58520000	0.58580000	0.00000000
357	3229.61170000	5.26230000	0.00000000
358	3229.61590000	5.34930000	0.00000000
359	3231.06310000	0.30530000	0.00000000
360	3231.15020000	3.33560000	0.00000000
361	3231.21640000	11.72060000	0.00000000
362	3231.30500000	0.00010000	0.00000000
363	3234.13900000	6.25180000	0.00000000
364	3234.15010000	11.95110000	0.00000000
365	3234.38950000	0.15670000	0.00000000
366	3234.40470000	0.03450000	0.00000000

S8. CALCULATIONS ON 2^{4+} (^{3}A) IN MeCN

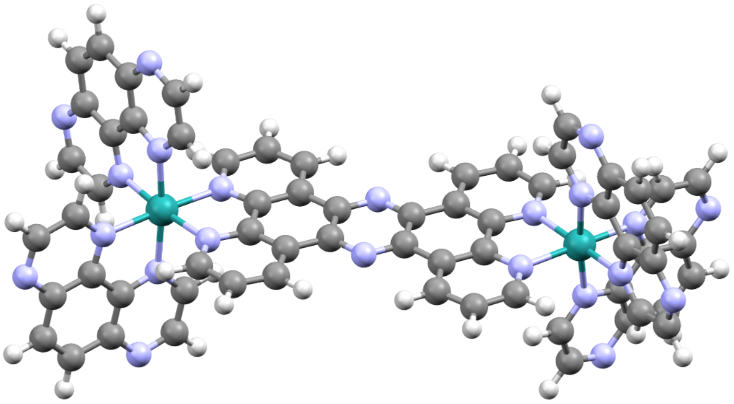

Route	: # opt freq b3lyp/genecp scrf=(solvent=acetonitrile) geom=connectivity	
	: empiricaldispersion=gd3bj int=ultrafine pop=regular	
SMILES	: c1cc2c3c(c4ccc[n+]5c4c2[n+](c1)[Ru]567([n+]8ccnc9c8c1[n+]6ccnc1cc9)[n+]1ccnc2c1c1[n+]7ccnc1cc2)nc1c2ccc[n+]4c2c2c(c1n3)ccc[n+]2[Ru]412([n+]3ccnc4c3c3[n+]1ccnc3cc4)[n+]1ccnc3c1c1[n+]2ccnc1cc3	
Formula	: $C_{64}H_{36}N_{22}Ru_2^{4+,3}$	
Charge	: 4	
Multiplicity	: 3	
Energy	: -3855.84374892	a.u.
Gibbs Energy	: -3855.02329800	a.u.
Number of imaginary frequencies	: 2	

S8.1. Cartesian Co-ordinates (XYZ format)

124

Ru	6.40251398	-0.00062600	-0.01198300
N	4.78503084	-0.06406200	-1.35669804
N	-0.02729600	-0.09500600	-1.40506601
N	-0.01941900	0.11045200	1.37960804
N	4.79194212	0.06861400	1.30105805
C	4.83978319	-0.14752200	-2.68888807
H	5.82208014	-0.16664401	-3.13846993
C	3.68440795	-0.19635700	-3.47043991
H	3.77901101	-0.25823000	-4.54531288
C	2.44429207	-0.16339099	-2.86049509
H	1.53022301	-0.19793400	-3.43695593
C	2.37211394	-0.08279400	-1.46204603
C	1.11640799	-0.04089000	-0.72583199
C	-1.16759002	-0.04909800	-0.71987301
C	-3.62506509	-0.06057400	-0.72238398
C	-3.62175703	0.06480500	0.71451300
C	-2.42085099	0.12663101	1.43321395
C	-1.16379404	0.06246700	0.70076102

C	1.12056100	0.05734300	0.69368899
C	2.38199902	0.09887000	1.42080998
C	2.46626306	0.18944600	2.81816292
H	1.55658603	0.23178700	3.40112209
C	3.71101594	0.22569200	3.41997409
H	3.81216788	0.29931700	4.49346495
C	4.86110020	0.16744100	2.63267493
H	5.84997606	0.19265901	3.06671095
C	3.57535791	0.04305100	0.69281602
C	3.57167792	-0.03600600	-0.74327701
C	7.52021122	-2.48468900	-1.06657898
C	5.76594400	-3.05895710	0.30107901
C	7.77664518	-3.83987093	-1.34495604
C	8.31058598	-1.46984899	-1.65690696
C	6.03268480	-4.41503477	0.00865100
H	4.96336603	-2.77135491	0.96486801
C	9.37761974	-1.80337501	-2.51097107
H	5.41159201	-5.18091679	0.45758301
C	8.76001835	0.77035397	-1.90788496
C	9.83203697	0.41817001	-2.75863290
H	8.52667904	1.80087101	-1.68201005
H	10.43803406	1.20529997	-3.19111395
N	8.00445175	-0.17644501	-1.37010300
N	6.51432180	-2.10473800	-0.23366401
C	8.17565441	1.50045395	1.70758200
C	8.65983677	-0.75045300	2.03096199
C	9.19535160	1.84430897	2.63991189
C	7.43850422	2.47159791	1.05058706
C	9.65233421	-0.37688100	2.92640209
H	8.46210670	-1.79110003	1.81449199
C	7.62725401	3.85415792	1.32333004
H	10.24432564	-1.14204705	3.41190100
C	5.73461914	3.01945901	-0.44423100
C	5.94469690	4.35137177	-0.14624500
H	4.98116016	2.71258998	-1.15546298
H	5.33813095	5.10565805	-0.63085598
N	7.89508486	0.17454500	1.42494905
N	6.49239016	2.05384302	0.12502600
C	-4.89290380	-0.24035200	-2.65722990
C	-4.88175678	0.23947200	2.65495992
C	-2.42753196	-0.11715800	-1.44656003
C	-2.49938798	-0.24012500	-2.84162211
H	-1.58641005	-0.28718501	-3.41863990
C	-2.48725200	0.25006601	2.82851100
H	-1.57208896	0.30151400	3.40172791
C	-3.74145103	-0.30015501	-3.44410610
C	-3.72693706	0.30505401	3.43647003
H	-3.84062195	-0.39465699	-4.51640797
H	-5.87584591	-0.28983200	-3.10251999
H	-3.82162905	0.39996201	4.50914288
H	-5.86300421	0.28478399	3.10445690
N	-4.84182119	-0.12582199	-1.32672298
N	-4.83613300	0.12451400	1.32430303
Ru	-6.45081615	-0.00510600	0.00221100
N	-7.93634796	0.04060800	-1.46622705
N	-6.56282091	2.04802489	-0.34326801
N	-7.93223381	-0.05921500	1.47453201
N	-6.55071592	-2.05890608	0.34774300
C	-8.20509148	1.30639303	-1.89463401
C	-8.63841057	-0.94077599	-2.02096701
C	-7.47215509	2.36729693	-1.30719995
C	-5.88595104	3.05367708	0.19910499
C	-8.19468403	-1.32673097	1.90175295
C	-8.63866901	0.91813999	2.03077793

C	-7.45735121	-2.38353491	1.31242204
C	-5.86933899	-3.06074500	-0.19611900
C	-9.16844463	1.56908798	-2.88567591
C	-9.60309982	-0.66230297	-3.01200008
H	-8.44469547	-1.95290303	-1.69629204
C	-7.69584990	3.69649196	-1.70966005
C	-6.11738300	4.38122892	-0.21790899
H	-5.16195679	2.82337809	0.96736300
C	-9.15643024	-1.59523296	2.89275694
C	-9.60155201	0.63387501	3.02194595
H	-8.45025158	1.93152702	1.70682800
C	-7.67447186	-3.71428204	1.71342802
C	-6.09386683	-4.38978195	0.21975900
H	-5.14719391	-2.82617593	-0.96482003
H	-10.15881920	-1.48412395	-3.44842005
H	-5.54960394	5.18191814	0.24173400
H	-10.16101933	1.45254004	3.45950794
H	-5.52256298	-5.18722391	-0.24117100
C	9.39976501	3.24750304	2.89954591
H	10.17114067	3.51248002	3.61137891
C	8.65340710	4.19931984	2.27835703
H	8.80341625	5.25224209	2.48081398
C	8.86284733	-4.16596603	-2.22286797
H	9.04700279	-5.21315908	-2.42221308
C	9.63259220	-3.18896198	-2.78003407
H	10.45638847	-3.42627406	-3.43985105
C	-8.65562439	-3.97286010	2.72786498
H	-8.80878258	-5.00224209	3.02396393
C	-9.36684513	-2.95702410	3.29236388
H	-10.10973454	-3.14395595	4.05651522
C	-8.67865753	3.94918990	-2.72401190
H	-8.83672810	4.97745323	-3.02142596
C	-9.38531017	2.92933512	-3.28700709
H	-10.12925720	3.11180210	-4.05120993
N	-6.99699593	4.71061802	-1.14698195
N	-9.87116909	0.55629700	-3.44568396
N	6.89192581	4.79088306	0.72780401
N	9.92581844	0.91055298	3.24557805
N	10.13936806	-0.82924199	-3.06089902
N	7.00952005	-4.80598021	-0.78831297
N	-9.86374283	-0.58648002	3.45425606
N	-6.97124386	-4.72444010	1.14907897

S8.2. Frequencies

Mode	IR frequency	IR intensity	Raman intensity
1	-9.68000000	0.88020000	0.00000000
2	-7.21580000	0.45570000	0.00000000
3	8.34590000	0.46530000	0.00000000
4	19.55890000	3.49510000	0.00000000
5	20.50170000	0.88950000	0.00000000
6	26.97130000	0.53150000	0.00000000
7	27.49290000	0.26110000	0.00000000
8	35.84120000	1.74850000	0.00000000
9	38.45170000	0.16340000	0.00000000
10	41.31770000	1.31950000	0.00000000
11	44.83780000	0.02950000	0.00000000
12	47.00700000	0.00640000	0.00000000
13	49.56810000	0.17630000	0.00000000
14	51.93960000	1.20180000	0.00000000
15	64.48410000	0.27580000	0.00000000
16	72.46230000	2.92530000	0.00000000
17	77.26580000	0.12070000	0.00000000
18	84.79960000	8.37310000	0.00000000
19	87.27720000	0.33490000	0.00000000
20	96.93360000	1.32020000	0.00000000
21	100.37080000	0.87520000	0.00000000
22	104.63340000	0.89820000	0.00000000
23	128.63530000	0.84430000	0.00000000
24	134.18500000	75.38000000	0.00000000
25	146.19790000	0.99560000	0.00000000
26	157.14540000	1.18140000	0.00000000
27	164.09780000	4.29340000	0.00000000
28	171.10520000	1.00190000	0.00000000
29	172.89390000	1.76830000	0.00000000
30	174.28830000	0.22500000	0.00000000
31	178.50120000	1.02650000	0.00000000
32	180.72840000	0.53330000	0.00000000
33	183.89360000	1.23240000	0.00000000
34	185.56390000	2.17400000	0.00000000
35	189.12260000	0.60580000	0.00000000
36	190.95490000	1.58330000	0.00000000
37	193.00640000	5.52930000	0.00000000
38	194.79050000	0.08300000	0.00000000
39	201.62750000	3.89480000	0.00000000
40	206.45270000	5.21790000	0.00000000
41	222.36440000	8.70240000	0.00000000
42	224.21600000	16.70750000	0.00000000
43	224.92740000	3.16890000	0.00000000
44	228.61960000	34.15650000	0.00000000
45	232.33790000	6.15440000	0.00000000
46	238.38020000	4.38610000	0.00000000
47	243.56410000	0.36680000	0.00000000
48	248.93390000	0.44940000	0.00000000
49	258.34600000	25.26240000	0.00000000
50	272.67330000	15.34020000	0.00000000
51	282.04020000	11.71640000	0.00000000
52	285.75750000	9.37450000	0.00000000
53	286.50030000	11.82890000	0.00000000
54	288.86610000	1.39390000	0.00000000
55	293.59160000	3.98710000	0.00000000
56	294.25670000	8.29860000	0.00000000
57	296.87980000	1.48320000	0.00000000
58	302.90250000	0.78710000	0.00000000
59	319.44580000	11.64090000	0.00000000
60	325.35290000	3.31080000	0.00000000

61	331.02890000	26.78130000	0.00000000
62	338.98420000	2.26890000	0.00000000
63	342.91630000	0.43110000	0.00000000
64	350.00930000	0.78270000	0.00000000
65	351.06940000	2.51410000	0.00000000
66	387.86100000	5.39960000	0.00000000
67	390.82550000	33.75070000	0.00000000
68	392.28450000	272.40850000	0.00000000
69	418.85570000	112.29110000	0.00000000
70	439.16890000	25.02250000	0.00000000
71	443.18000000	23.91770000	0.00000000
72	445.57700000	4.15140000	0.00000000
73	446.84400000	0.09680000	0.00000000
74	447.48750000	0.62860000	0.00000000
75	447.57230000	13.22390000	0.00000000
76	451.19740000	47.37320000	0.00000000
77	452.74080000	19.13840000	0.00000000
78	454.96700000	28.74190000	0.00000000
79	456.84970000	114.31550000	0.00000000
80	458.93620000	31.43740000	0.00000000
81	460.22400000	1.69190000	0.00000000
82	466.47730000	38.35420000	0.00000000
83	474.65280000	51.31530000	0.00000000
84	476.80330000	54.23280000	0.00000000
85	480.79730000	44.35440000	0.00000000
86	484.49310000	16.51260000	0.00000000
87	487.83420000	9.88040000	0.00000000
88	491.98060000	0.74950000	0.00000000
89	498.39620000	14.36780000	0.00000000
90	502.21330000	7.38610000	0.00000000
91	504.25190000	14.05560000	0.00000000
92	523.96650000	14.42590000	0.00000000
93	536.79340000	13.32880000	0.00000000
94	543.00570000	8.20230000	0.00000000
95	545.45370000	9.88710000	0.00000000
96	551.83500000	65.86950000	0.00000000
97	553.92130000	0.57630000	0.00000000
98	554.71490000	4.63090000	0.00000000
99	554.78850000	15.36710000	0.00000000
100	554.88190000	7.17730000	0.00000000
101	555.67770000	0.30330000	0.00000000
102	561.28180000	0.00130000	0.00000000
103	565.52260000	0.35290000	0.00000000
104	571.56270000	0.82360000	0.00000000
105	576.65970000	19.03810000	0.00000000
106	582.11600000	39.47680000	0.00000000
107	582.26600000	5.82690000	0.00000000
108	584.23650000	11.12970000	0.00000000
109	585.06570000	5.49270000	0.00000000
110	592.09150000	1.67670000	0.00000000
111	593.38560000	11.24080000	0.00000000
112	594.11620000	0.06410000	0.00000000
113	594.86110000	3.28510000	0.00000000
114	610.61240000	4.60450000	0.00000000
115	618.90310000	2.05320000	0.00000000
116	634.82160000	10.00060000	0.00000000
117	640.57160000	0.58930000	0.00000000
118	643.42350000	517.12030000	0.00000000
119	644.29840000	0.06930000	0.00000000
120	662.23780000	2.74190000	0.00000000
121	663.26790000	0.97860000	0.00000000
122	665.80150000	2.22510000	0.00000000
123	671.10130000	0.03560000	0.00000000
124	671.87940000	7.38410000	0.00000000

125	677.03480000	3.95070000	0.00000000
126	678.16800000	2.68840000	0.00000000
127	708.07900000	25.01820000	0.00000000
128	719.87060000	1.04210000	0.00000000
129	726.68740000	1.56680000	0.00000000
130	741.63440000	203.86980000	0.00000000
131	744.25380000	31.33040000	0.00000000
132	746.42980000	33.44410000	0.00000000
133	749.53510000	13.68510000	0.00000000
134	750.12680000	26.43180000	0.00000000
135	751.01990000	121.52550000	0.00000000
136	753.04160000	5.14270000	0.00000000
137	753.65510000	4.34580000	0.00000000
138	753.88870000	0.45560000	0.00000000
139	754.04580000	2.94860000	0.00000000
140	757.71870000	4.01400000	0.00000000
141	759.19800000	9.20270000	0.00000000
142	760.18810000	23.16210000	0.00000000
143	761.81650000	14.12310000	0.00000000
144	763.77530000	10.16790000	0.00000000
145	766.06110000	13.90660000	0.00000000
146	805.20250000	0.98220000	0.00000000
147	806.94370000	35.94240000	0.00000000
148	811.97740000	0.07150000	0.00000000
149	818.17830000	0.81990000	0.00000000
150	820.34970000	10.77310000	0.00000000
151	822.87440000	0.88220000	0.00000000
152	825.82200000	2.92010000	0.00000000
153	835.65620000	102.13340000	0.00000000
154	841.81600000	17.94490000	0.00000000
155	853.06060000	0.83260000	0.00000000
156	853.95620000	0.13620000	0.00000000
157	854.24890000	0.13500000	0.00000000
158	855.54070000	0.82050000	0.00000000
159	858.38210000	2.49340000	0.00000000
160	859.73350000	89.45330000	0.00000000
161	859.83600000	0.76470000	0.00000000
162	861.12240000	0.20940000	0.00000000
163	873.19740000	57.32000000	0.00000000
164	878.68980000	2.12380000	0.00000000
165	881.35610000	2.33070000	0.00000000
166	882.02940000	6.61970000	0.00000000
167	882.90750000	8.86270000	0.00000000
168	894.15250000	80.71800000	0.00000000
169	894.83720000	94.36930000	0.00000000
170	895.08880000	93.90260000	0.00000000
171	907.86250000	1249.64860000	0.00000000
172	923.16310000	13.75620000	0.00000000
173	925.55510000	0.52160000	0.00000000
174	926.49090000	76.57900000	0.00000000
175	927.51570000	0.32280000	0.00000000
176	946.13360000	0.67440000	0.00000000
177	948.30050000	0.75380000	0.00000000
178	949.11290000	1.95700000	0.00000000
179	950.12890000	6.32740000	0.00000000
180	950.83690000	5.90620000	0.00000000
181	958.00230000	0.99880000	0.00000000
182	979.67780000	0.06530000	0.00000000
183	981.03530000	0.05540000	0.00000000
184	984.95890000	1353.02870000	0.00000000
185	992.40970000	0.61370000	0.00000000
186	993.47030000	14.99070000	0.00000000
187	997.95600000	0.24880000	0.00000000
188	998.41980000	0.23660000	0.00000000

189	1002.08750000	0.11020000	0.00000000
190	1002.68230000	0.11110000	0.00000000
191	1002.76350000	0.09740000	0.00000000
192	1002.87580000	0.68920000	0.00000000
193	1006.03220000	0.04590000	0.00000000
194	1016.49590000	0.00850000	0.00000000
195	1016.52610000	0.00710000	0.00000000
196	1016.93850000	0.01350000	0.00000000
197	1026.86220000	0.15320000	0.00000000
198	1028.00230000	0.08020000	0.00000000
199	1034.27940000	0.31270000	0.00000000
200	1035.16430000	0.20710000	0.00000000
201	1048.24040000	9.29130000	0.00000000
202	1051.16350000	0.21420000	0.00000000
203	1052.74940000	1.08340000	0.00000000
204	1052.97860000	1.04400000	0.00000000
205	1063.90220000	19.30800000	0.00000000
206	1066.50280000	4.24160000	0.00000000
207	1071.14440000	1.38470000	0.00000000
208	1074.25330000	3.46650000	0.00000000
209	1079.60060000	7.49940000	0.00000000
210	1080.55970000	1.72530000	0.00000000
211	1087.51290000	78.28850000	0.00000000
212	1090.84890000	56.25100000	0.00000000
213	1104.96570000	7.95650000	0.00000000
214	1114.78950000	276.79490000	0.00000000
215	1119.76930000	53.46180000	0.00000000
216	1122.10930000	104.60210000	0.00000000
217	1122.21900000	55.52620000	0.00000000
218	1124.71880000	67.15160000	0.00000000
219	1125.46240000	121.66200000	0.00000000
220	1126.15830000	60.16710000	0.00000000
221	1126.83760000	25.29690000	0.00000000
222	1127.80840000	384.58470000	0.00000000
223	1128.68440000	71.39180000	0.00000000
224	1131.14250000	123.69240000	0.00000000
225	1150.89660000	531.53980000	0.00000000
226	1153.66050000	40.81460000	0.00000000
227	1159.81980000	144.27150000	0.00000000
228	1162.90580000	11.02820000	0.00000000
229	1165.74730000	18.90350000	0.00000000
230	1169.88190000	509.67560000	0.00000000
231	1184.45850000	3.23560000	0.00000000
232	1186.96340000	0.06660000	0.00000000
233	1190.23690000	6.86530000	0.00000000
234	1211.31980000	14.93690000	0.00000000
235	1216.82350000	9.79100000	0.00000000
236	1228.53820000	11.70960000	0.00000000
237	1230.78300000	5.03110000	0.00000000
238	1233.43720000	3.94220000	0.00000000
239	1234.92400000	1.02920000	0.00000000
240	1241.07570000	7.18000000	0.00000000
241	1248.27600000	2.31320000	0.00000000
242	1251.09670000	7.01290000	0.00000000
243	1251.69460000	20.59850000	0.00000000
244	1253.26190000	33.92880000	0.00000000
245	1264.66000000	1.38670000	0.00000000
246	1265.22990000	2.09790000	0.00000000
247	1265.58390000	2.87660000	0.00000000
248	1265.81650000	3.19660000	0.00000000
249	1271.88370000	13.88860000	0.00000000
250	1275.97240000	101.03880000	0.00000000
251	1286.34980000	21.34900000	0.00000000
252	1309.09910000	144.72860000	0.00000000

253	1309.62230000	55.32580000	0.00000000
254	1310.12240000	88.06340000	0.00000000
255	1310.63370000	5.43340000	0.00000000
256	1313.25310000	51.11480000	0.00000000
257	1315.03460000	17.97070000	0.00000000
258	1316.24180000	1.17200000	0.00000000
259	1318.91000000	1.50450000	0.00000000
260	1324.61460000	4.42060000	0.00000000
261	1327.72850000	47.93330000	0.00000000
262	1333.83330000	1.48570000	0.00000000
263	1338.25970000	0.80910000	0.00000000
264	1354.71210000	17.89600000	0.00000000
265	1355.44220000	39.62510000	0.00000000
266	1361.75490000	9.65100000	0.00000000
267	1372.75370000	3.31390000	0.00000000
268	1373.79180000	0.55470000	0.00000000
269	1374.35650000	0.46140000	0.00000000
270	1388.25040000	208.06550000	0.00000000
271	1389.80150000	275.17820000	0.00000000
272	1395.45570000	13.41070000	0.00000000
273	1413.11480000	91.63160000	0.00000000
274	1414.05210000	42.50840000	0.00000000
275	1414.19350000	71.20340000	0.00000000
276	1425.42210000	85.44960000	0.00000000
277	1428.80870000	22.41310000	0.00000000
278	1429.00020000	68.31880000	0.00000000
279	1429.03100000	67.60020000	0.00000000
280	1431.15280000	509.46920000	0.00000000
281	1437.00120000	173.92940000	0.00000000
282	1445.33950000	96.99830000	0.00000000
283	1446.66030000	26.14220000	0.00000000
284	1447.01260000	42.80840000	0.00000000
285	1459.42220000	2.63610000	0.00000000
286	1467.00280000	38.58640000	0.00000000
287	1471.66390000	0.15290000	0.00000000
288	1473.70060000	622.34630000	0.00000000
289	1475.24000000	211.34270000	0.00000000
290	1488.47870000	3.09330000	0.00000000
291	1490.85700000	18.31010000	0.00000000
292	1491.02450000	3.63890000	0.00000000
293	1491.06690000	4.33780000	0.00000000
294	1493.91440000	243.39600000	0.00000000
295	1502.03370000	19.89420000	0.00000000
296	1510.26580000	5.38840000	0.00000000
297	1519.33340000	147.06870000	0.00000000
298	1521.45690000	180.58260000	0.00000000
299	1521.74710000	81.07670000	0.00000000
300	1522.14720000	13.62580000	0.00000000
301	1525.98050000	191.83930000	0.00000000
302	1535.44820000	229.78250000	0.00000000
303	1535.85330000	25.07640000	0.00000000
304	1536.68100000	110.24160000	0.00000000
305	1540.85990000	38.89390000	0.00000000
306	1545.00280000	0.20460000	0.00000000
307	1550.04620000	22.73390000	0.00000000
308	1565.14360000	77.99150000	0.00000000
309	1582.96630000	6.61180000	0.00000000
310	1583.17250000	5.01550000	0.00000000
311	1584.89540000	4.62660000	0.00000000
312	1585.69350000	68.64380000	0.00000000
313	1586.00740000	20.85730000	0.00000000
314	1587.43910000	0.82780000	0.00000000
315	1590.46340000	31.83070000	0.00000000
316	1606.04900000	18.41670000	0.00000000

317	1614.34500000	1.39610000	0.00000000
318	1615.10550000	21.26110000	0.00000000
319	1617.12350000	1.40330000	0.00000000
320	1621.64290000	2.39760000	0.00000000
321	1622.26280000	27.66220000	0.00000000
322	1623.81690000	17.34110000	0.00000000
323	1626.37780000	0.57460000	0.00000000
324	1634.72220000	4.45630000	0.00000000
325	1640.67630000	7.53730000	0.00000000
326	1642.44380000	13.77000000	0.00000000
327	1649.18830000	18.72260000	0.00000000
328	1654.15450000	8.02750000	0.00000000
329	1655.57200000	15.15330000	0.00000000
330	1656.82240000	6.21040000	0.00000000
331	3187.47140000	12.18460000	0.00000000
332	3187.66480000	10.33960000	0.00000000
333	3189.84140000	13.92440000	0.00000000
334	3189.90350000	6.31200000	0.00000000
335	3193.44880000	50.90910000	0.00000000
336	3194.36970000	9.04330000	0.00000000
337	3194.47910000	1.73320000	0.00000000
338	3194.80780000	23.61390000	0.00000000
339	3194.91330000	0.47270000	0.00000000
340	3205.12800000	0.18850000	0.00000000
341	3205.17670000	0.18610000	0.00000000
342	3208.46340000	14.66770000	0.00000000
343	3209.46810000	0.76940000	0.00000000
344	3215.30410000	0.12000000	0.00000000
345	3215.44540000	0.24910000	0.00000000
346	3217.23330000	4.36320000	0.00000000
347	3217.28040000	3.19780000	0.00000000
348	3218.85060000	0.25880000	0.00000000
349	3219.25760000	0.14450000	0.00000000
350	3220.97250000	0.84320000	0.00000000
351	3221.72650000	23.77510000	0.00000000
352	3224.21910000	16.98270000	0.00000000
353	3225.35810000	6.88070000	0.00000000
354	3225.71190000	6.56100000	0.00000000
355	3227.77290000	3.45230000	0.00000000
356	3228.15380000	5.48590000	0.00000000
357	3232.13920000	2.18120000	0.00000000
358	3232.34620000	6.49510000	0.00000000
359	3232.43210000	2.04210000	0.00000000
360	3233.18740000	4.28800000	0.00000000
361	3233.79490000	4.41030000	0.00000000
362	3234.55250000	5.59310000	0.00000000
363	3234.86450000	1.04090000	0.00000000
364	3235.04140000	2.74990000	0.00000000
365	3235.32300000	1.67590000	0.00000000
366	3235.85520000	4.60450000	0.00000000

S9. CALCULATIONS ON 2^{4+} (^{1}A) AT ^{3}A STRUCTURE IN MeCN

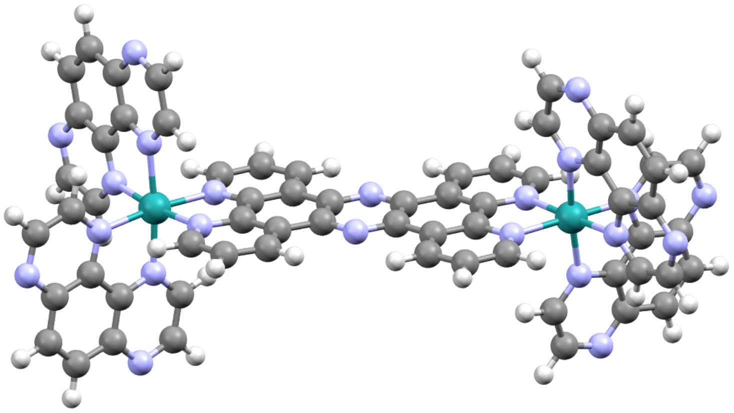

Route	: # b3lyp/genecp scrf=(solvent=acetonitrile) geom=connectivity empirical	
	: dispersion=gd3bj int=ultrafine pop=regular	
SMILES	:	
Formula	: $C_{64}H_{36}N_{22}Ru_2^{4+}$	
Charge	: 4	
Multiplicity	: 1	
Energy	: -3855.91420118	a.u.

S9.1. Cartesian Co-ordinates (XYZ format)

124

Ru	-6.40251398	0.00062600	-0.01198300
N	-4.78503084	0.06406200	-1.35669804
N	0.02729600	0.09500600	-1.40506601
N	0.01941900	-0.11045200	1.37960804
N	-4.79194212	-0.06861400	1.30105805
C	-4.83978319	0.14752200	-2.68888807
H	-5.82208014	0.16664401	-3.13846993
C	-3.68440795	0.19635700	-3.47043991
H	-3.77901101	0.25823000	-4.54531288
C	-2.44429207	0.16339099	-2.86049509
H	-1.53022301	0.19793400	-3.43695593
C	-2.37211394	0.08279400	-1.46204603
C	-1.11640799	0.04089000	-0.72583199
C	1.16759002	0.04909800	-0.71987301
C	3.62506509	0.06057400	-0.72238398
C	3.62175703	-0.06480500	0.71451300
C	2.42085099	-0.12663101	1.43321395
C	1.16379404	-0.06246700	0.70076102
C	-1.12056100	-0.05734300	0.69368899
C	-2.38199902	-0.09887000	1.42080998
C	-2.46626306	-0.18944600	2.81816292
H	-1.55658603	-0.23178700	3.40112209
C	-3.71101594	-0.22569200	3.41997409

H	-3.81216788	-0.29931700	4.49346495
C	-4.86110020	-0.16744100	2.63267493
H	-5.84997606	-0.19265901	3.06671095
C	-3.57535791	-0.04305100	0.69281602
C	-3.57167792	0.03600600	-0.74327701
C	-7.52021122	2.48468900	-1.06657898
C	-5.76594400	3.05895710	0.30107901
C	-7.77664518	3.83987093	-1.34495604
C	-8.31058598	1.46984899	-1.65690696
C	-6.03268480	4.41503477	0.00865100
H	-4.96336603	2.77135491	0.96486801
C	-9.37761974	1.80337501	-2.51097107
H	-5.41159201	5.18091679	0.45758301
C	-8.76001835	-0.77035397	-1.90788496
C	-9.83203697	-0.41817001	-2.75863290
H	-8.52667904	-1.80087101	-1.68201005
H	-10.43803406	-1.20529997	-3.19111395
N	-8.00445175	0.17644501	-1.37010300
N	-6.51432180	2.10473800	-0.23366401
C	-8.17565441	-1.50045395	1.70758200
C	-8.65983677	0.75045300	2.03096199
C	-9.19535160	-1.84430897	2.63991189
C	-7.43850422	-2.47159791	1.05058706
C	-9.65233421	0.37688100	2.92640209
H	-8.46210670	1.79110003	1.81449199
C	-7.62725401	-3.85415792	1.32333004
H	-10.24432564	1.14204705	3.41190100
C	-5.73461914	-3.01945901	-0.44423100
C	-5.94469690	-4.35137177	-0.14624500
H	-4.98116016	-2.71258998	-1.15546298
H	-5.33813095	-5.10565805	-0.63085598
N	-7.89508486	-0.17454500	1.42494905
N	-6.49239016	-2.05384302	0.12502600
C	4.89290380	0.24035200	-2.65722990
C	4.88175678	-0.23947200	2.65495992
C	2.42753196	0.11715800	-1.44656003
C	2.49938798	0.24012500	-2.84162211
H	1.58641005	0.28718501	-3.41863990
C	2.48725200	-0.25006601	2.82851100
H	1.57208896	-0.30151400	3.40172791
C	3.74145103	0.30015501	-3.44410610
C	3.72693706	-0.30505401	3.43647003
H	3.84062195	0.39465699	-4.51640797
H	5.87584591	0.28983200	-3.10251999
H	3.82162905	-0.39996201	4.50914288
H	5.86300421	-0.28478399	3.10445690
N	4.84182119	0.12582199	-1.32672298
N	4.83613300	-0.12451400	1.32430303
Ru	6.45081615	0.00510600	0.00221100
N	7.93634796	-0.04060800	-1.46622705
N	6.56282091	-2.04802489	-0.34326801
N	7.93223381	0.05921500	1.47453201
N	6.55071592	2.05890608	0.34774300
C	8.20509148	-1.30639303	-1.89463401
C	8.63841057	0.94077599	-2.02096701
C	7.47215509	-2.36729693	-1.30719995
C	5.88595104	-3.05367708	0.19910499
C	8.19468403	1.32673097	1.90175295
C	8.63866901	-0.91813999	2.03077793
C	7.45735121	2.38353491	1.31242204
C	5.86933899	3.06074500	-0.19611900
C	9.16844463	-1.56908798	-2.88567591
C	9.60309982	0.66230297	-3.01200008
H	8.44469547	1.95290303	-1.69629204

C	7.69584990	-3.69649196	-1.70966005
C	6.11738300	-4.38122892	-0.21790899
H	5.16195679	-2.82337809	0.96736300
C	9.15643024	1.59523296	2.89275694
C	9.60155201	-0.63387501	3.02194595
H	8.45025158	-1.93152702	1.70682800
C	7.67447186	3.71428204	1.71342802
C	6.09386683	4.38978195	0.21975900
H	5.14719391	2.82617593	-0.96482003
H	10.15881920	1.48412395	-3.44842005
H	5.54960394	-5.18191814	0.24173400
H	10.16101933	-1.45254004	3.45950794
H	5.52256298	5.18722391	-0.24117100
C	-9.39976501	-3.24750304	2.89954591
H	-10.17114067	-3.51248002	3.61137891
C	-8.65340710	-4.19931984	2.27835703
H	-8.80341625	-5.25224209	2.48081398
C	-8.86284733	4.16596603	-2.22286797
H	-9.04700279	5.21315908	-2.42221308
C	-9.63259220	3.18896198	-2.78003407
H	-10.45638847	3.42627406	-3.43985105
C	8.65562439	3.97286010	2.72786498
H	8.80878258	5.00224209	3.02396393
C	9.36684513	2.95702410	3.29236388
H	10.10973454	3.14395595	4.05651522
C	8.67865753	-3.94918990	-2.72401190
H	8.83672810	-4.97745323	-3.02142596
C	9.38531017	-2.92933512	-3.28700709
H	10.12925720	-3.11180210	-4.05120993
N	6.99699593	-4.71061802	-1.14698195
N	9.87116909	-0.55629700	-3.44568396
N	-6.89192581	-4.79088306	0.72780401
N	-9.92581844	-0.91055298	3.24557805
N	-10.13936806	0.82924199	-3.06089902
N	-7.00952005	4.80598021	-0.78831297
N	9.86374283	0.58648002	3.45425606
N	6.97124386	4.72444010	1.14907897

S10. CALCULATIONS ON 2^{4+} (^{1}A) IN WATER (EXPLICIT + PCM)

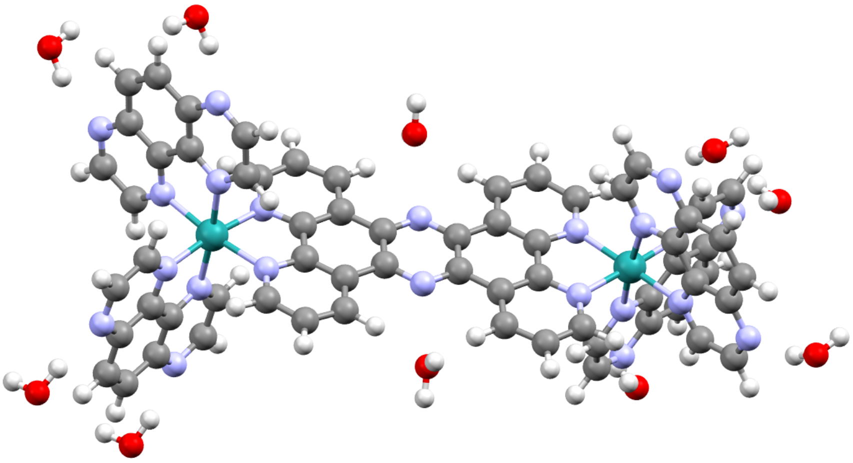

Route	: # opt freq b3lyp/genecp scrf=(solvent=water) geom=connectivity empiric
	: aldispersion=gd3bj int=ultrafine pop=regular
SMILES	: c1cc2c3c(c4ccc[n+]5c4c2[n+](c1)[Ru]567([n+]8ccnc9c8c1[n+]6ccnc1cc9)[n+]1ccnc2c1c1[n+]7ccnc1cc2)nc1c2ccc[n+]4c2c2c(c1n3)ccc[n+]2[Ru]412([n+]3ccnc4c3c3[n+]1ccnc3cc4)[n+]1ccnc3c1c1[n+]2ccnc1cc3.O.O.O.O.O.O.O.O.O.O
Formula	: $C_{64}H_{56}N_{22}O_{10}Ru_2^{4+}$
Charge	: 4
Multiplicity	: 1
Energy	: -4620.61294951 a.u.
Gibbs Energy	: -4619.59213800 a.u.
Number of imaginary frequencies	: 2

S10.1. Cartesian Co-ordinates (XYZ format)

154

Ru	6.42090511	0.00522900	-0.00712200
N	4.80897284	0.09718400	-1.33584702
N	-0.00571700	0.04646300	-1.40217495
N	-0.00613700	-0.05438000	1.38513696
N	4.80866385	-0.08888500	1.32097995
C	4.85820198	0.17442501	-2.66920590
H	5.84033489	0.21657200	-3.11724496
C	3.70481610	0.20503500	-3.45403910
H	3.80204296	0.26753500	-4.52920580
C	2.46032190	0.15679400	-2.85284209
H	1.55157006	0.17930999	-3.44514799
C	2.39433694	0.07716200	-1.45265400
C	1.13693404	0.02912200	-0.71932602
C	-1.14857996	0.01447000	-0.72027498
C	-3.60414195	-0.00184400	-0.73071098
C	-3.60442090	-0.01810100	0.71208298
C	-2.40625310	-0.03680700	1.43772304
C	-1.14882195	-0.02713400	0.70275998

C	1.13672602	-0.03271300	0.70278299
C	2.39393711	-0.07633500	1.43669605
C	2.45956993	-0.15527400	2.83693099
H	1.55049598	-0.18020700	3.42876506
C	3.70391989	-0.19896100	3.43874788
H	3.80082393	-0.26030901	4.51401186
C	4.85754299	-0.16519800	2.65440297
H	5.83960915	-0.20408300	3.10288310
C	3.59273005	-0.04085000	0.71246099
C	3.59291410	0.04533800	-0.72790700
C	7.42011499	-2.39665699	-1.27428102
C	5.83124304	-3.04140210	0.24915600
C	7.63022709	-3.73211789	-1.65953302
C	8.16079235	-1.35336697	-1.88201904
C	6.04402494	-4.37779903	-0.14677000
H	5.11255217	-2.79086995	1.01547599
C	9.11818886	-1.64641201	-2.86905289
H	5.47107601	-5.16687918	0.32479700
C	8.61610222	0.88832802	-2.04611707
C	9.57892704	0.58580202	-3.03093791
H	8.43161488	1.90757406	-1.73929703
H	10.14586067	1.39117396	-3.48171997
N	7.90482807	-0.07780600	-1.47593498
N	6.51832581	-2.05291796	-0.31190300
C	8.15974712	1.36529100	1.86763203
C	8.61574745	-0.87618297	2.03285289
C	9.11692238	1.65911996	2.85466695
C	7.41861391	2.40801692	1.25948095
C	9.57834625	-0.57286298	3.01763391
H	8.43152714	-1.89563096	1.72651696
C	7.62857008	3.74376988	1.64376903
H	10.14466953	-1.37794995	3.46969700
C	5.82915306	3.05140495	-0.26391801
C	6.04167223	4.38810301	0.13117100
H	5.11033583	2.80022097	-1.02990997
H	5.46833801	5.17673397	-0.34067801
N	7.90432501	0.08947400	1.46202898
N	6.51676893	2.06345892	0.29743201
C	-4.86999178	0.00558400	-2.67588902
C	-4.87121391	-0.02991400	2.65668297
C	-2.40569496	0.01938400	-1.45579004
C	-2.47210598	0.04096600	-2.85812712
H	-1.56335294	0.06389600	-3.45068908
C	-2.47334290	-0.05984600	2.84000897
H	-1.56507194	-0.08031300	3.43318200
C	-3.71673608	0.03373900	-3.46105099
C	-3.71829295	-0.05635600	3.44237494
H	-3.81408691	0.04866700	-4.53791380
H	-5.85245514	-0.00780000	-3.12506509
H	-3.81605101	-0.07231000	4.51919079
H	-5.85406399	-0.01874600	3.10507989
N	-4.82022381	-0.01215400	-1.34049296
N	-4.82078218	-0.01058300	1.32136202
Ru	-6.43257284	-0.00879100	-0.00958000
N	-7.91364384	0.17576499	-1.47226501
N	-6.52043104	2.06567693	-0.18200999
N	-7.91667414	-0.18587300	1.45071399
N	-6.53286123	-2.08273911	0.16091999
C	-8.15726471	1.47567403	-1.80193806
C	-8.63179016	-0.74818301	-2.10086489
C	-7.41287804	2.47440195	-1.12753403
C	-5.83182716	3.01313305	0.44414499
C	-8.17241764	-1.48473704	1.77515900
C	-8.63008690	0.74193698	2.07905197

C	-7.43306303	-2.48731089	1.10094094
C	-5.84906387	-3.03378010	-0.46511200
C	-9.10724354	1.83571506	-2.77385592
C	-9.58825970	-0.37842000	-3.06887007
H	-8.45703793	-1.78569901	-1.85582602
C	-7.61142588	3.83254600	-1.43113899
C	-6.03331518	4.37291098	0.13025101
H	-5.12017679	2.71186590	1.19865596
C	-9.13158321	-1.84019494	2.73972392
C	-9.59542656	0.37687400	3.03995609
H	-8.44518757	1.77873003	1.83835399
C	-7.64594507	-3.84486103	1.39730406
C	-6.06469488	-4.39298487	-0.15814599
H	-5.13103580	-2.73577404	-1.21486104
H	-10.16109276	-1.15004694	-3.56859398
H	-5.45921803	5.12737799	0.65423203
H	-10.16408825	1.15161097	3.53964591
H	-5.49424410	-5.15042019	-0.68181598
C	9.32039928	3.02661991	3.23831701
H	10.06312466	3.22072005	4.00200987
C	8.60409069	4.02896500	2.65660095
H	8.74107456	5.06698179	2.93273592
C	8.60509205	-4.01640081	-2.67324805
H	8.74149895	-5.05410290	-2.95085001
C	9.32198238	-3.01363993	-3.25352311
H	10.06259727	-3.20680094	-4.01948500
C	-8.62150669	-4.19302702	2.39016891
H	-8.76492882	-5.24668694	2.59421611
C	-9.33736515	-3.22922897	3.03416896
H	-10.08286762	-3.47105598	3.78132200
C	-8.57698345	4.18529510	-2.43212295
H	-8.70426846	5.23895979	-2.64671493
C	-9.29780006	3.22524405	-3.07621288
H	-10.03145790	3.47021890	-3.83395410
N	-6.89734411	4.78316498	-0.78065199
N	-9.82446384	0.87616998	-3.40741491
N	6.91583395	4.73661804	1.05805802
N	9.82811260	0.65768498	3.42728901
N	9.82895184	-0.64444703	-3.44128895
N	6.91790199	-4.72549677	-1.07422805
N	-9.84512806	-0.87692100	3.37175488
N	-6.93786192	-4.79927301	0.74580699
O	-0.00481900	-0.15035801	4.95246983
O	-0.00061100	0.19781300	-4.96509790
H	-0.02733600	-0.87209499	5.59064198
H	0.04270400	0.64843398	5.48952007
H	-0.07856400	0.99954200	-5.49410677
H	0.00344700	-0.51949698	-5.60863304
O	11.68898678	-1.93449402	-5.28986692
O	7.90687180	-7.21591091	-2.24139595
O	7.90245390	7.22758007	2.22625494
O	11.51377964	1.92776299	5.44946480
O	-11.63536072	2.29309106	-5.21430779
O	-7.86593819	7.34877014	-1.79167998
O	-11.54640484	-2.27464604	5.29620409
O	-7.79935789	-7.35579586	1.87276399
H	-10.91143417	-2.38237810	6.01276684
H	-7.24028778	-7.38042021	2.65701199
H	-12.34461975	2.50636101	-4.59814501
H	-8.64847279	7.45102978	-1.23904502
H	8.68356323	-7.35332489	-1.68815601
H	10.87194061	1.98748398	6.16554117
H	8.67893028	7.36717415	1.67325604
H	12.38537216	-2.17671800	-4.66971922

H	7.42507410	6.51444292	1.76636004
H	11.12436676	-1.33557200	-4.76984406
H	7.42827988	-6.50290394	-1.78256202
H	11.07543468	1.34453106	4.80482721
H	-7.39449215	6.60463381	-1.37697506
H	-11.09024811	1.65780795	-4.71717501
H	-7.42781115	-6.61895895	1.35601795
H	-11.10187531	-1.65069199	4.69543505

S10.2. Frequencies

Mode	IR frequency	IR intensity	Raman intensity
1	-8.29050000	0.64710000	0.00000000
2	-7.59230000	0.01660000	0.00000000
3	4.42130000	0.40050000	0.00000000
4	8.61150000	3.14620000	0.00000000
5	11.95240000	4.77450000	0.00000000
6	15.63630000	5.24460000	0.00000000
7	17.12380000	2.23020000	0.00000000
8	18.39480000	5.49730000	0.00000000
9	20.14180000	4.31570000	0.00000000
10	21.61570000	0.68700000	0.00000000
11	24.42950000	0.44410000	0.00000000
12	25.56310000	0.49070000	0.00000000
13	28.87380000	0.36140000	0.00000000
14	37.89060000	0.42400000	0.00000000
15	42.35980000	5.07090000	0.00000000
16	43.40050000	4.48490000	0.00000000
17	46.61350000	6.15520000	0.00000000
18	47.71900000	0.29240000	0.00000000
19	47.95490000	3.03240000	0.00000000
20	50.75830000	6.08410000	0.00000000
21	52.14860000	3.94950000	0.00000000
22	52.53220000	3.12500000	0.00000000
23	53.53890000	2.12410000	0.00000000
24	56.49110000	0.81430000	0.00000000
25	64.42890000	40.25050000	0.00000000
26	64.64660000	31.30500000	0.00000000
27	66.41790000	29.52450000	0.00000000
28	66.85960000	32.04220000	0.00000000
29	67.49300000	185.50630000	0.00000000
30	69.09670000	22.93880000	0.00000000
31	69.46390000	333.75660000	0.00000000
32	70.69470000	37.31620000	0.00000000
33	76.37690000	0.13710000	0.00000000
34	82.59400000	16.07580000	0.00000000
35	83.37320000	1.40820000	0.00000000
36	84.13080000	4.75560000	0.00000000
37	85.38980000	3.28430000	0.00000000
38	90.24220000	16.37510000	0.00000000
39	90.97420000	17.90950000	0.00000000
40	92.66430000	22.37900000	0.00000000
41	97.65870000	7.51820000	0.00000000
42	99.51690000	14.36890000	0.00000000
43	101.18630000	7.93540000	0.00000000
44	102.29130000	5.41980000	0.00000000
45	114.37950000	2.24780000	0.00000000
46	117.68930000	10.11600000	0.00000000
47	128.17410000	1.64250000	0.00000000
48	130.80160000	0.17910000	0.00000000
49	131.90920000	0.09790000	0.00000000
50	136.45770000	0.62310000	0.00000000
51	139.17480000	8.94740000	0.00000000
52	140.32050000	0.42380000	0.00000000
53	145.02740000	31.99770000	0.00000000
54	146.24020000	2.32700000	0.00000000
55	147.85120000	3.39900000	0.00000000
56	149.28890000	1.84900000	0.00000000
57	153.31330000	139.22690000	0.00000000
58	155.80010000	38.02630000	0.00000000
59	173.57740000	3.65360000	0.00000000
60	177.60160000	0.83830000	0.00000000

61	179.10640000	0.33280000	0.00000000
62	179.89360000	2.80820000	0.00000000
63	182.52880000	4.93900000	0.00000000
64	183.07680000	6.49980000	0.00000000
65	185.95570000	2.36660000	0.00000000
66	189.38270000	1.18490000	0.00000000
67	193.99790000	0.54950000	0.00000000
68	195.17660000	1.09760000	0.00000000
69	197.65620000	0.56470000	0.00000000
70	200.14200000	6.08890000	0.00000000
71	204.37300000	3.14990000	0.00000000
72	207.11810000	0.66500000	0.00000000
73	207.98220000	4.62770000	0.00000000
74	212.25950000	12.48840000	0.00000000
75	214.61400000	9.45540000	0.00000000
76	215.24920000	14.15400000	0.00000000
77	216.67490000	39.40980000	0.00000000
78	217.04250000	64.13410000	0.00000000
79	224.24520000	1.64750000	0.00000000
80	227.05240000	0.54310000	0.00000000
81	232.66820000	0.15940000	0.00000000
82	240.35340000	316.97630000	0.00000000
83	243.46360000	271.95060000	0.00000000
84	243.99640000	288.98550000	0.00000000
85	244.51280000	20.46360000	0.00000000
86	246.94770000	3.33360000	0.00000000
87	250.08850000	1.44560000	0.00000000
88	251.78480000	0.59810000	0.00000000
89	261.21570000	1.97680000	0.00000000
90	263.67090000	11.58330000	0.00000000
91	264.89460000	185.05110000	0.00000000
92	266.30170000	33.55950000	0.00000000
93	287.92670000	6.91570000	0.00000000
94	288.84810000	5.46640000	0.00000000
95	290.03050000	3.29850000	0.00000000
96	291.19850000	12.57090000	0.00000000
97	297.79610000	17.31250000	0.00000000
98	299.44690000	12.67510000	0.00000000
99	301.16010000	29.27350000	0.00000000
100	302.70610000	8.74190000	0.00000000
101	306.12570000	2.99450000	0.00000000
102	319.56290000	13.99520000	0.00000000
103	320.26370000	4.02810000	0.00000000
104	326.44680000	12.84920000	0.00000000
105	337.44380000	51.37260000	0.00000000
106	338.76430000	114.60150000	0.00000000
107	341.95650000	2.31200000	0.00000000
108	343.24820000	37.89890000	0.00000000
109	343.94140000	6.55630000	0.00000000
110	345.62360000	105.75100000	0.00000000
111	346.36470000	124.45730000	0.00000000
112	348.01890000	67.89740000	0.00000000
113	348.42160000	125.53230000	0.00000000
114	352.08150000	51.02200000	0.00000000
115	354.07890000	53.55950000	0.00000000
116	354.71150000	103.24380000	0.00000000
117	356.40290000	48.55570000	0.00000000
118	390.54820000	1.08020000	0.00000000
119	393.59130000	0.60000000	0.00000000
120	442.84810000	8.85010000	0.00000000
121	446.86680000	0.00820000	0.00000000
122	447.67880000	0.25500000	0.00000000
123	447.84660000	0.14320000	0.00000000
124	448.45450000	0.15160000	0.00000000

125	448.64510000	0.23970000	0.00000000
126	449.13750000	7.48410000	0.00000000
127	457.85200000	11.77060000	0.00000000
128	458.63420000	7.97130000	0.00000000
129	461.76110000	1.82580000	0.00000000
130	466.49880000	0.87400000	0.00000000
131	466.90710000	4.61670000	0.00000000
132	470.44020000	0.97810000	0.00000000
133	474.52820000	1.05170000	0.00000000
134	481.28640000	4.25200000	0.00000000
135	486.99620000	9.26530000	0.00000000
136	488.15270000	32.84530000	0.00000000
137	488.51430000	17.93840000	0.00000000
138	490.42780000	1.84160000	0.00000000
139	497.68360000	0.11590000	0.00000000
140	501.09460000	3.70970000	0.00000000
141	506.02190000	0.19320000	0.00000000
142	506.64390000	7.65100000	0.00000000
143	509.54460000	0.02130000	0.00000000
144	536.96820000	10.50080000	0.00000000
145	555.39030000	0.03110000	0.00000000
146	561.50610000	3.10400000	0.00000000
147	563.31870000	16.93050000	0.00000000
148	563.51400000	4.49300000	0.00000000
149	563.72770000	12.27580000	0.00000000
150	564.11540000	8.72490000	0.00000000
151	564.80170000	2.10620000	0.00000000
152	570.60730000	1.30460000	0.00000000
153	570.77300000	0.68580000	0.00000000
154	573.47260000	0.24210000	0.00000000
155	576.24170000	1.29400000	0.00000000
156	586.61320000	68.81390000	0.00000000
157	586.76750000	61.25600000	0.00000000
158	588.71340000	2.15450000	0.00000000
159	588.78330000	33.44650000	0.00000000
160	594.11390000	3.45760000	0.00000000
161	595.57690000	0.79530000	0.00000000
162	596.62010000	0.24870000	0.00000000
163	596.99840000	0.72090000	0.00000000
164	597.77600000	6.03890000	0.00000000
165	599.17590000	8.59810000	0.00000000
166	624.31510000	0.00410000	0.00000000
167	643.98870000	0.20790000	0.00000000
168	644.70560000	0.74090000	0.00000000
169	647.61020000	0.20630000	0.00000000
170	648.02730000	0.02800000	0.00000000
171	662.41810000	0.00310000	0.00000000
172	663.93070000	1.60470000	0.00000000
173	667.01630000	126.59570000	0.00000000
174	667.91210000	223.72620000	0.00000000
175	670.65490000	55.17490000	0.00000000
176	670.89110000	163.64190000	0.00000000
177	671.36680000	86.49590000	0.00000000
178	674.12170000	59.94190000	0.00000000
179	678.63900000	59.45250000	0.00000000
180	679.50700000	61.42760000	0.00000000
181	680.04400000	41.47530000	0.00000000
182	681.91630000	51.20890000	0.00000000
183	686.43240000	690.66010000	0.00000000
184	687.32300000	648.29380000	0.00000000
185	688.31270000	174.23140000	0.00000000
186	689.24910000	263.61010000	0.00000000
187	720.54430000	0.12170000	0.00000000
188	733.05870000	0.00420000	0.00000000

189	743.78310000	0.00850000	0.00000000
190	747.35720000	8.99750000	0.00000000
191	754.02630000	1.09730000	0.00000000
192	754.04150000	0.69910000	0.00000000
193	754.13240000	0.38290000	0.00000000
194	754.15380000	0.57960000	0.00000000
195	758.02680000	78.66160000	0.00000000
196	758.44190000	130.61610000	0.00000000
197	762.27920000	121.59680000	0.00000000
198	765.22880000	40.16370000	0.00000000
199	766.66840000	0.81450000	0.00000000
200	768.79670000	18.93400000	0.00000000
201	768.98910000	41.85280000	0.00000000
202	772.46130000	2.52320000	0.00000000
203	773.03180000	3.35840000	0.00000000
204	773.83870000	4.33550000	0.00000000
205	774.42190000	2.27680000	0.00000000
206	823.35220000	0.18280000	0.00000000
207	827.60190000	0.00530000	0.00000000
208	830.27480000	0.05410000	0.00000000
209	832.55800000	0.03460000	0.00000000
210	845.42540000	148.71150000	0.00000000
211	848.35050000	2.97380000	0.00000000
212	854.70970000	0.36360000	0.00000000
213	855.40170000	1.12510000	0.00000000
214	855.57200000	0.14440000	0.00000000
215	856.24200000	0.13000000	0.00000000
216	861.62720000	0.03090000	0.00000000
217	871.54960000	25.01010000	0.00000000
218	871.76520000	26.63770000	0.00000000
219	871.92840000	23.83590000	0.00000000
220	872.99180000	22.24230000	0.00000000
221	874.80350000	13.01690000	0.00000000
222	882.64780000	0.08370000	0.00000000
223	882.68840000	0.14410000	0.00000000
224	883.61860000	0.89650000	0.00000000
225	883.84060000	0.17240000	0.00000000
226	883.91320000	0.00900000	0.00000000
227	921.12930000	71.99050000	0.00000000
228	921.42270000	69.94430000	0.00000000
229	921.58950000	72.51700000	0.00000000
230	922.01140000	76.00100000	0.00000000
231	925.86720000	9.02070000	0.00000000
232	925.95310000	1.23600000	0.00000000
233	927.93030000	4.45950000	0.00000000
234	927.96430000	4.13900000	0.00000000
235	948.43970000	0.01860000	0.00000000
236	950.18950000	0.07650000	0.00000000
237	951.14170000	1.19620000	0.00000000
238	952.11450000	1.06100000	0.00000000
239	952.22040000	2.28520000	0.00000000
240	994.65260000	0.03190000	0.00000000
241	995.43700000	0.02050000	0.00000000
242	995.71580000	0.07890000	0.00000000
243	995.98780000	0.03340000	0.00000000
244	999.82740000	0.16530000	0.00000000
245	999.87570000	0.22290000	0.00000000
246	1000.08650000	0.19960000	0.00000000
247	1000.43530000	0.28180000	0.00000000
248	1001.85360000	0.31140000	0.00000000
249	1001.92140000	0.29850000	0.00000000
250	1002.53570000	0.25430000	0.00000000
251	1002.87390000	0.37070000	0.00000000
252	1051.63300000	0.26370000	0.00000000

253	1056.61570000	0.31100000	0.00000000
254	1057.78200000	0.22630000	0.00000000
255	1058.22480000	0.83530000	0.00000000
256	1058.96470000	1.88690000	0.00000000
257	1064.44640000	0.00250000	0.00000000
258	1066.39930000	0.15040000	0.00000000
259	1070.92450000	5.56860000	0.00000000
260	1075.00160000	1.60150000	0.00000000
261	1077.38260000	0.42780000	0.00000000
262	1078.41350000	4.04290000	0.00000000
263	1078.92430000	0.70260000	0.00000000
264	1079.30200000	0.39350000	0.00000000
265	1079.36800000	0.05000000	0.00000000
266	1079.61520000	0.74050000	0.00000000
267	1079.88610000	0.70360000	0.00000000
268	1081.80450000	8.63420000	0.00000000
269	1082.85220000	3.41290000	0.00000000
270	1083.30090000	8.95720000	0.00000000
271	1084.06370000	2.71770000	0.00000000
272	1091.90130000	54.57790000	0.00000000
273	1112.61080000	0.04520000	0.00000000
274	1128.26280000	35.46900000	0.00000000
275	1129.58230000	59.95520000	0.00000000
276	1129.62620000	34.49710000	0.00000000
277	1130.49280000	40.09710000	0.00000000
278	1131.90810000	25.19850000	0.00000000
279	1134.75270000	7.70190000	0.00000000
280	1137.47870000	6.39500000	0.00000000
281	1137.77400000	5.64290000	0.00000000
282	1138.86860000	18.27690000	0.00000000
283	1142.29960000	0.21410000	0.00000000
284	1144.34450000	3.76700000	0.00000000
285	1154.27390000	0.15740000	0.00000000
286	1157.96360000	211.33120000	0.00000000
287	1167.06840000	27.58850000	0.00000000
288	1170.27900000	0.24840000	0.00000000
289	1195.78540000	0.30940000	0.00000000
290	1196.22400000	0.18480000	0.00000000
291	1197.29310000	0.09070000	0.00000000
292	1197.82250000	0.07340000	0.00000000
293	1218.11620000	11.80740000	0.00000000
294	1233.49690000	6.17090000	0.00000000
295	1236.87700000	0.04880000	0.00000000
296	1237.03190000	1.27620000	0.00000000
297	1237.07900000	0.56590000	0.00000000
298	1238.43560000	0.10500000	0.00000000
299	1238.70410000	0.05040000	0.00000000
300	1260.98320000	3.99860000	0.00000000
301	1261.78020000	11.58550000	0.00000000
302	1262.42970000	5.78070000	0.00000000
303	1262.90700000	12.01290000	0.00000000
304	1264.59230000	33.47490000	0.00000000
305	1264.70910000	30.76390000	0.00000000
306	1264.88170000	12.28120000	0.00000000
307	1265.01410000	20.74280000	0.00000000
308	1268.07740000	1.54110000	0.00000000
309	1290.19140000	5.54780000	0.00000000
310	1306.78470000	0.00340000	0.00000000
311	1313.08890000	175.33350000	0.00000000
312	1313.42890000	93.73300000	0.00000000
313	1314.91550000	107.63840000	0.00000000
314	1314.97510000	92.79510000	0.00000000
315	1315.08620000	124.95490000	0.00000000
316	1315.10840000	74.58730000	0.00000000

317	1315.77790000	2.46900000	0.00000000
318	1316.02650000	1.68050000	0.00000000
319	1316.23480000	10.67160000	0.00000000
320	1323.25200000	1.27510000	0.00000000
321	1324.00180000	42.06760000	0.00000000
322	1341.55470000	0.09260000	0.00000000
323	1344.42750000	1.37170000	0.00000000
324	1355.95150000	0.00220000	0.00000000
325	1361.38560000	1.84310000	0.00000000
326	1376.93910000	0.47020000	0.00000000
327	1377.57890000	0.21330000	0.00000000
328	1378.54110000	0.77120000	0.00000000
329	1378.99970000	0.04480000	0.00000000
330	1391.37990000	299.64570000	0.00000000
331	1396.99650000	0.23580000	0.00000000
332	1422.34430000	86.78840000	0.00000000
333	1422.56770000	60.82310000	0.00000000
334	1422.66780000	73.69430000	0.00000000
335	1422.74430000	65.81760000	0.00000000
336	1431.15150000	18.05060000	0.00000000
337	1431.24060000	25.42370000	0.00000000
338	1431.29680000	57.16990000	0.00000000
339	1431.33910000	66.20230000	0.00000000
340	1439.78310000	227.14560000	0.00000000
341	1443.66090000	105.26790000	0.00000000
342	1445.22570000	109.78590000	0.00000000
343	1445.39920000	26.43350000	0.00000000
344	1446.59470000	27.58420000	0.00000000
345	1459.89880000	0.30640000	0.00000000
346	1471.03680000	0.00980000	0.00000000
347	1477.41440000	42.55380000	0.00000000
348	1488.87700000	0.00410000	0.00000000
349	1493.72760000	1.59950000	0.00000000
350	1493.92140000	0.84070000	0.00000000
351	1493.98050000	3.52460000	0.00000000
352	1494.07620000	16.59900000	0.00000000
353	1501.49670000	34.08420000	0.00000000
354	1511.54330000	1.29120000	0.00000000
355	1524.45290000	238.35050000	0.00000000
356	1524.66760000	236.04450000	0.00000000
357	1524.79150000	222.27540000	0.00000000
358	1525.04500000	93.78840000	0.00000000
359	1525.53640000	6.40260000	0.00000000
360	1536.01310000	10.51170000	0.00000000
361	1537.03290000	169.56350000	0.00000000
362	1537.78690000	140.41210000	0.00000000
363	1541.56060000	44.99600000	0.00000000
364	1542.95380000	39.73930000	0.00000000
365	1550.90250000	0.00810000	0.00000000
366	1585.45910000	13.11970000	0.00000000
367	1585.46860000	15.13990000	0.00000000
368	1585.59680000	8.31370000	0.00000000
369	1585.63820000	8.86700000	0.00000000
370	1586.45290000	19.84330000	0.00000000
371	1587.12170000	54.74100000	0.00000000
372	1587.34080000	90.28490000	0.00000000
373	1587.92480000	25.74790000	0.00000000
374	1587.98730000	15.98050000	0.00000000
375	1603.82820000	7.94150000	0.00000000
376	1613.78150000	0.34300000	0.00000000
377	1615.25250000	105.21100000	0.00000000
378	1615.72150000	26.42970000	0.00000000
379	1618.04580000	20.28160000	0.00000000
380	1618.53110000	23.43670000	0.00000000

381	1619.87690000	1.68130000	0.00000000
382	1620.36280000	0.12540000	0.00000000
383	1622.83320000	40.15260000	0.00000000
384	1624.22000000	13.69110000	0.00000000
385	1626.37680000	0.15660000	0.00000000
386	1632.94700000	0.00710000	0.00000000
387	1639.11970000	0.01230000	0.00000000
388	1647.76020000	18.70850000	0.00000000
389	1658.97270000	179.88980000	0.00000000
390	1659.64720000	329.46920000	0.00000000
391	1660.18860000	9.19400000	0.00000000
392	1660.20800000	75.33300000	0.00000000
393	1660.44640000	14.52080000	0.00000000
394	1661.18180000	82.58810000	0.00000000
395	1661.45470000	78.50230000	0.00000000
396	1662.65120000	72.95060000	0.00000000
397	1662.73040000	75.18140000	0.00000000
398	1662.97270000	12.47490000	0.00000000
399	1663.48040000	3.09710000	0.00000000
400	1663.88240000	85.28170000	0.00000000
401	3164.01670000	151.91580000	0.00000000
402	3165.06520000	182.69080000	0.00000000
403	3170.60480000	243.32510000	0.00000000
404	3170.78400000	28.86100000	0.00000000
405	3192.87050000	35.36480000	0.00000000
406	3193.33860000	35.52760000	0.00000000
407	3193.80390000	35.60980000	0.00000000
408	3193.86210000	36.24270000	0.00000000
409	3196.30960000	5.31490000	0.00000000
410	3196.37170000	5.25710000	0.00000000
411	3196.97580000	5.63990000	0.00000000
412	3196.98560000	5.99090000	0.00000000
413	3197.01480000	5.90150000	0.00000000
414	3197.10880000	3.84460000	0.00000000
415	3198.10650000	7.09610000	0.00000000
416	3198.17660000	3.31160000	0.00000000
417	3202.02110000	9.46670000	0.00000000
418	3202.59790000	9.61030000	0.00000000
419	3203.01040000	9.57780000	0.00000000
420	3203.05950000	9.60630000	0.00000000
421	3213.23590000	1.78500000	0.00000000
422	3213.36420000	3.15480000	0.00000000
423	3213.47300000	0.38010000	0.00000000
424	3213.52120000	2.38270000	0.00000000
425	3227.70870000	6.89390000	0.00000000
426	3227.89200000	2.63860000	0.00000000
427	3228.83270000	9.53620000	0.00000000
428	3228.92880000	0.57170000	0.00000000
429	3233.14070000	2.80150000	0.00000000
430	3233.68610000	3.05150000	0.00000000
431	3233.90490000	2.84900000	0.00000000
432	3234.06610000	3.02290000	0.00000000
433	3234.72380000	4.51220000	0.00000000
434	3235.57640000	2.82640000	0.00000000
435	3238.22220000	6.84920000	0.00000000
436	3238.45520000	0.37350000	0.00000000
437	3616.17170000	256.20940000	0.00000000
438	3616.45760000	761.82930000	0.00000000
439	3616.48960000	653.61360000	0.00000000
440	3616.75050000	260.59310000	0.00000000
441	3617.07960000	1604.70080000	0.00000000
442	3617.52650000	1862.58230000	0.00000000
443	3618.08180000	1091.21140000	0.00000000
444	3618.21750000	733.86910000	0.00000000

445	3800.25250000	39.06740000	0.00000000
446	3800.58310000	37.82020000	0.00000000
447	3846.79010000	83.81190000	0.00000000
448	3847.27130000	75.65940000	0.00000000
449	3847.52940000	101.18890000	0.00000000
450	3847.53230000	3.67860000	0.00000000
451	3847.55650000	119.32760000	0.00000000
452	3847.62050000	105.45890000	0.00000000
453	3847.63100000	171.42880000	0.00000000
454	3848.16280000	94.50570000	0.00000000
455	3892.79330000	87.80870000	0.00000000
456	3893.06720000	88.56530000	0.00000000

S11. CALCULATIONS ON 2^{4+} (^{3}A) IN WATER (EXPLICIT + PCM)

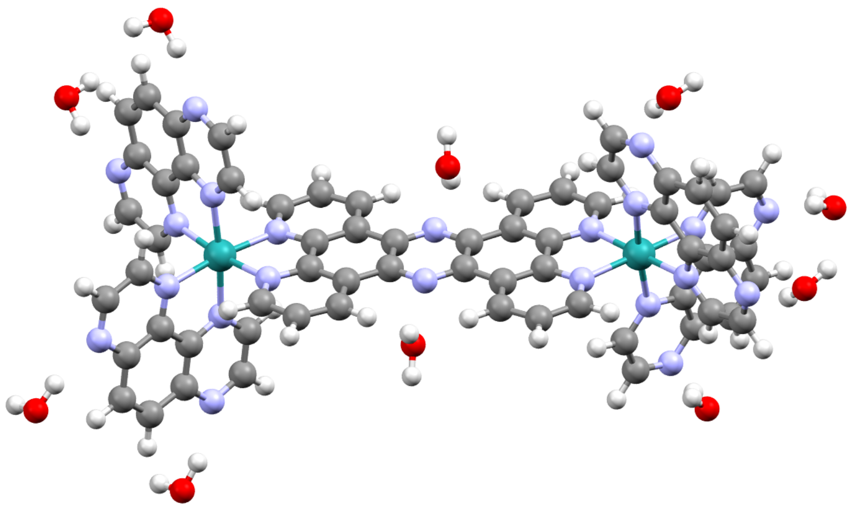

Route	: # opt freq b3lyp/genecp scrf=(solvent=water) geom=connectivity empiric	
	: aldispersion=gd3bj int=ultrafine pop=regular	
SMILES	: c1cc2c3c(c4ccc[n+]5c4c2[n+](c1)[Ru]567([n+]8ccnc9c8c1[n+]6ccnc1cc9)[n+]1ccnc2c1c1[n+]7ccnc1cc2)nc1c2ccc[n+]4c2c2c(c1n3)ccc[n+]2[Ru]412([n+]3ccnc4c3c3[n+]1ccnc3cc4)[n+]1ccnc3c1c1[n+]2ccnc1cc3.O.O.O.O.O.O.O.O.O.O	
Formula	: $C_{64}H_{56}N_{22}O_{10}Ru_2^{4+,3}$	
Charge	: 4	
Multiplicity	: 3	
Energy	: -4620.53783411	a.u.
Gibbs Energy	: -4619.52119800	a.u.
Number of imaginary frequencies	: 3	

S11.1. Cartesian Co-ordinates (XYZ format)

154

Ru	6.45210886	0.01507200	-0.00355500
N	4.84286499	0.12706199	-1.33341002
N	0.03033600	0.07950000	-1.41216195
N	0.02214200	-0.11628200	1.37125897
N	4.83709717	-0.11477100	1.31860495
C	4.89455080	0.23560500	-2.66462803
H	5.87750292	0.29004201	-3.10941195
C	3.74307203	0.28316799	-3.45126200
H	3.84277511	0.37150699	-4.52435303
C	2.49734807	0.21948200	-2.85408807
H	1.58913803	0.25675499	-3.44678092
C	2.42928100	0.10585300	-1.45643401
C	1.17058396	0.03832500	-0.72741401
C	-1.11348295	0.02606100	-0.73394501
C	-3.56589699	0.01755200	-0.75171500
C	-3.57007694	-0.05825500	0.68449402
C	-2.37818193	-0.11327300	1.41518295
C	-1.11786497	-0.06940200	0.68583697

C	1.16661096	-0.06626600	0.69350302
C	2.42235804	-0.12164500	1.42903900
C	2.48473191	-0.23486300	2.82698488
H	1.57381201	-0.28344700	3.41482711
C	3.72810102	-0.28379801	3.43058896
H	3.82321000	-0.37123501	4.50417805
C	4.88314009	-0.22214700	2.65011692
H	5.86452198	-0.26438099	3.09975195
C	3.62243104	-0.06081800	0.70852602
C	3.62582994	0.05847300	-0.72968698
C	7.46537590	-2.35834002	-1.31319201
C	5.87985706	-3.03967595	0.19783400
C	7.68294382	-3.68534589	-1.72274399
C	8.20028114	-1.30009198	-1.90177798
C	6.10042286	-4.36749315	-0.22208500
H	5.15972424	-2.80721593	0.96858603
C	9.15818119	-1.56955898	-2.89505196
H	5.53194380	-5.16826010	0.23495600
C	8.64182377	0.94681501	-2.02654505
C	9.60453701	0.66801602	-3.01845193
H	8.45146942	1.95926201	-1.70161402
H	10.16533375	1.48487794	-3.45602393
N	7.93737602	-0.03354500	-1.47260201
N	6.56104183	-2.03718495	-0.34541801
C	8.18505573	1.34948599	1.89525497
C	8.64708233	-0.89299703	2.02445292
C	9.14196301	1.62998998	2.88641405
C	7.44039679	2.39985490	1.30475795
C	9.60837746	-0.60313499	3.01456690
H	8.46588898	-1.90782404	1.70156300
C	7.64699697	3.72982001	1.71044302
H	10.17628860	-1.41401994	3.45408392
C	5.84788322	3.06363702	-0.20672700
C	6.05679607	4.39428616	0.21003100
H	5.12929392	2.82315207	-0.97643298
H	5.48067522	5.18886089	-0.24824600
N	7.93337107	0.07971100	1.46889198
N	6.53876305	2.06851292	0.33789000
C	-4.83650923	0.12751999	-2.69688797
C	-4.85895109	-0.17969701	2.62334394
C	-2.36775994	0.06641500	-1.47281206
C	-2.43683600	0.15051199	-2.87280297
H	-1.52830803	0.19388101	-3.46564102
C	-2.45971608	-0.20551901	2.81417990
H	-1.55578804	-0.25287101	3.41396308
C	-3.68145394	0.18055300	-3.47700691
C	-3.70933199	-0.24026901	3.40955591
H	-3.77697492	0.24472900	-4.55196810
H	-5.81914091	0.14428400	-3.14563394
H	-3.81173205	-0.31432801	4.48321915
H	-5.84798908	-0.20147499	3.05707502
N	-4.78036308	0.04327200	-1.36426699
N	-4.78834009	-0.08149000	1.29110503
Ru	-6.39388418	-0.01053700	-0.02241400
N	-7.98725891	0.16365600	-1.39683795
N	-6.49348783	2.09266591	-0.26141900
N	-7.89415216	-0.17615700	1.41447401
N	-6.48762989	-2.06714106	0.13064000
C	-8.27418041	1.45620501	-1.70427001
C	-8.74343967	-0.78313202	-1.93351305
C	-7.48230219	2.47025108	-1.11492205
C	-5.74430609	3.04507709	0.27512699
C	-8.17120171	-1.49951100	1.70845604
C	-8.65277672	0.75378197	2.01861191

C	-7.43373203	-2.47589493	1.05869901
C	-5.73307180	-3.03509402	-0.43585199
C	-9.32289505	1.79386199	-2.57816291
C	-9.79873371	-0.43329200	-2.80526900
H	-8.52418423	-1.81315100	-1.69222903
C	-7.72391987	3.82249594	-1.41673195
C	-5.99147606	4.40022087	-0.03687600
H	-4.95528793	2.75818801	0.95496798
C	-9.18481445	-1.84145105	2.64468193
C	-9.64247990	0.39005601	2.92127299
H	-8.45503426	1.79257500	1.79459798
C	-7.62718391	-3.85284710	1.34396899
C	-5.94250202	-4.36608601	-0.13205400
H	-4.97996902	-2.73278308	-1.14912701
H	-10.40820980	-1.21659005	-3.23833609
H	-5.37298298	5.16788101	0.41115099
H	-10.23331642	1.15505302	3.40671897
H	-5.34037590	-5.12682009	-0.61027497
C	9.34239292	2.99173188	3.29153299
H	10.08525753	3.17548394	4.05764484
C	8.62293530	4.00136900	2.72659492
H	8.75710297	5.03514290	3.01948905
C	8.65894985	-3.94560003	-2.74183702
H	8.80049419	-4.97722197	-3.03883410
C	9.36954594	-2.92836905	-3.30438805
H	10.11010838	-3.10317111	-4.07481003
C	-8.64972591	-4.19716311	2.30246091
H	-8.79556465	-5.25116682	2.50392008
C	-9.39382076	-3.24076796	2.91896009
H	-10.16724682	-3.49113011	3.63431692
C	-8.79107857	4.15312481	-2.31622910
H	-8.95140839	5.20303917	-2.52761698
C	-9.56227779	3.17705393	-2.87283897
H	-10.37444305	3.40385604	-3.55228996
N	-6.95218611	4.78418016	-0.85570103
N	-10.08329487	0.81482899	-3.12449288
N	6.93077707	4.73003006	1.14178801
N	9.85541439	0.62137097	3.44336295
N	9.86158752	-0.55319798	-3.45072889
N	6.97606993	-4.69318819	-1.15584695
N	-9.91022491	-0.89659399	3.24376702
N	-6.88986778	-4.79041290	0.74722803
O	-0.01487600	-0.42133799	4.89435387
O	0.01705300	0.34692600	-4.94518900
H	0.02607200	0.24491499	5.58945417
H	0.00284900	-1.26373601	5.36214781
H	0.05781500	-0.36321399	-5.59544086
H	0.01792000	1.15674698	-5.46767998
O	11.72029972	-1.79622197	-5.33273792
O	7.96470308	-7.15511608	-2.38408208
O	7.90007401	7.20405006	2.36095810
O	11.53895760	1.86403203	5.48429823
O	-12.11053276	2.23151803	-4.72119617
O	-8.08572006	7.32369280	-1.81805003
O	-11.68532276	-2.21572208	5.09142494
O	-7.69729614	-7.34356308	1.78436601
H	-11.07223988	-2.35599899	5.82102823
H	-7.19620609	-7.34898376	2.60696507
H	-12.75242233	2.42482805	-4.02904177
H	-8.79482174	7.43696213	-1.17555106
H	8.74129486	-7.30937815	-1.83518302
H	10.89750767	1.91134405	6.20164824
H	8.67480469	7.36358309	1.81093502
H	12.42304897	-2.04378390	-4.72195482

H	7.42905807	6.49714804	1.88518500
H	11.15488434	-1.21096206	-4.79825592
H	7.48798323	-6.45365620	-1.90594494
H	11.10163116	1.28950000	4.83123398
H	-7.54215717	6.61671400	-1.43093503
H	-11.51939297	1.58197904	-4.30434418
H	-7.34972286	-6.55799484	1.31677198
H	-11.18245125	-1.63598597	4.48657179

S11.2. Frequencies

Mode	IR frequency	IR intensity	Raman intensity
1	-18.14000000	45.07630000	0.00000000
2	-8.41230000	1.90570000	0.00000000
3	-5.75200000	0.34310000	0.00000000
4	5.08640000	0.63750000	0.00000000
5	8.93580000	5.64730000	0.00000000
6	9.69370000	6.80770000	0.00000000
7	11.17580000	6.41130000	0.00000000
8	16.48640000	2.71330000	0.00000000
9	17.21080000	1.43890000	0.00000000
10	19.20890000	2.18330000	0.00000000
11	19.93330000	4.54170000	0.00000000
12	22.59430000	0.55560000	0.00000000
13	26.62550000	2.60300000	0.00000000
14	35.08340000	12.89390000	0.00000000
15	36.76020000	0.57650000	0.00000000
16	39.82290000	4.16140000	0.00000000
17	41.46580000	3.58130000	0.00000000
18	45.52820000	3.93220000	0.00000000
19	46.60780000	4.18130000	0.00000000
20	49.42320000	4.63610000	0.00000000
21	51.39130000	2.72610000	0.00000000
22	52.08750000	1.56880000	0.00000000
23	54.43940000	1.53000000	0.00000000
24	56.69680000	1.60230000	0.00000000
25	64.73490000	0.49510000	0.00000000
26	65.61390000	0.49470000	0.00000000
27	66.43290000	2.43670000	0.00000000
28	67.78570000	1.06800000	0.00000000
29	69.12110000	3.17930000	0.00000000
30	70.82820000	6.43250000	0.00000000
31	75.80590000	38.20830000	0.00000000
32	77.53540000	12.09520000	0.00000000
33	81.44530000	28.38540000	0.00000000
34	83.57260000	258.59990000	0.00000000
35	84.58140000	4.28990000	0.00000000
36	86.41810000	158.83940000	0.00000000
37	87.41020000	195.77980000	0.00000000
38	88.92990000	5.45150000	0.00000000
39	90.46890000	5.83590000	0.00000000
40	93.76690000	54.76490000	0.00000000
41	96.71370000	57.28230000	0.00000000
42	99.03520000	5.34830000	0.00000000
43	101.82030000	7.58800000	0.00000000
44	104.24590000	0.48980000	0.00000000
45	104.62230000	10.61610000	0.00000000
46	113.64410000	9.97670000	0.00000000
47	116.82160000	11.88150000	0.00000000
48	128.00830000	0.31630000	0.00000000
49	128.86580000	0.44400000	0.00000000
50	131.75080000	0.43120000	0.00000000
51	133.08200000	29.54150000	0.00000000
52	137.27400000	7.36770000	0.00000000
53	139.44280000	48.66220000	0.00000000
54	140.80400000	28.12120000	0.00000000
55	147.81840000	3.10080000	0.00000000
56	148.50010000	40.53790000	0.00000000
57	149.77940000	26.58220000	0.00000000
58	152.13990000	46.87640000	0.00000000
59	155.13790000	14.05550000	0.00000000
60	170.22760000	3.49620000	0.00000000

61	178.28060000	1.17670000	0.00000000
62	179.01860000	4.76510000	0.00000000
63	180.56380000	1.33170000	0.00000000
64	181.66770000	4.80870000	0.00000000
65	184.46130000	1.86870000	0.00000000
66	188.99070000	0.66570000	0.00000000
67	190.13920000	4.87310000	0.00000000
68	195.22240000	7.24460000	0.00000000
69	196.92410000	4.50580000	0.00000000
70	198.39760000	7.22550000	0.00000000
71	202.69530000	8.87190000	0.00000000
72	206.75440000	7.86930000	0.00000000
73	208.74240000	10.06390000	0.00000000
74	210.49720000	30.63330000	0.00000000
75	212.32180000	13.79270000	0.00000000
76	216.42150000	48.36890000	0.00000000
77	217.68560000	45.68000000	0.00000000
78	218.50840000	20.77230000	0.00000000
79	227.86030000	0.59650000	0.00000000
80	230.85750000	9.25560000	0.00000000
81	231.24090000	0.08710000	0.00000000
82	238.81100000	193.21330000	0.00000000
83	239.49260000	409.97230000	0.00000000
84	243.75600000	5.84550000	0.00000000
85	245.61570000	241.34980000	0.00000000
86	246.18440000	71.83560000	0.00000000
87	249.42660000	3.83010000	0.00000000
88	251.35770000	2.43680000	0.00000000
89	260.47480000	10.95710000	0.00000000
90	263.55050000	8.97340000	0.00000000
91	264.92310000	20.45120000	0.00000000
92	265.39360000	192.47540000	0.00000000
93	267.29180000	19.46030000	0.00000000
94	276.74860000	30.54720000	0.00000000
95	288.22070000	8.54270000	0.00000000
96	288.44940000	6.89170000	0.00000000
97	290.89170000	11.51440000	0.00000000
98	294.23630000	23.54090000	0.00000000
99	296.62710000	40.78260000	0.00000000
100	298.92700000	7.15490000	0.00000000
101	301.68250000	8.68140000	0.00000000
102	304.47730000	2.86640000	0.00000000
103	317.84680000	92.87480000	0.00000000
104	319.85450000	25.42160000	0.00000000
105	320.31930000	111.00880000	0.00000000
106	327.48410000	3.22010000	0.00000000
107	333.93200000	23.25770000	0.00000000
108	338.28730000	71.82420000	0.00000000
109	340.68960000	21.52940000	0.00000000
110	343.33610000	2.79380000	0.00000000
111	345.12820000	131.19230000	0.00000000
112	347.72930000	57.17230000	0.00000000
113	351.12240000	25.17860000	0.00000000
114	353.75400000	44.67230000	0.00000000
115	356.74170000	94.01090000	0.00000000
116	363.41640000	148.47340000	0.00000000
117	373.52100000	105.11350000	0.00000000
118	389.06290000	8.75780000	0.00000000
119	391.29410000	3.87720000	0.00000000
120	408.04220000	425.37640000	0.00000000
121	428.81960000	169.53190000	0.00000000
122	441.77250000	20.35670000	0.00000000
123	444.82560000	21.49410000	0.00000000
124	447.06680000	11.89130000	0.00000000

125	447.85060000	0.46460000	0.00000000
126	448.72210000	0.15290000	0.00000000
127	449.01420000	19.10450000	0.00000000
128	452.93190000	60.01690000	0.00000000
129	457.61680000	55.21760000	0.00000000
130	458.19030000	52.75370000	0.00000000
131	459.46570000	70.38780000	0.00000000
132	461.87140000	115.13470000	0.00000000
133	466.65130000	3.91440000	0.00000000
134	469.41480000	21.27730000	0.00000000
135	476.19780000	114.09430000	0.00000000
136	479.16780000	38.96180000	0.00000000
137	483.16790000	49.65960000	0.00000000
138	487.86970000	20.18390000	0.00000000
139	490.19070000	7.49970000	0.00000000
140	496.96990000	0.24130000	0.00000000
141	500.34630000	3.33360000	0.00000000
142	506.26050000	4.23810000	0.00000000
143	506.50110000	4.09140000	0.00000000
144	530.05460000	21.63250000	0.00000000
145	536.97430000	10.02880000	0.00000000
146	552.00770000	23.25450000	0.00000000
147	554.31470000	6.06570000	0.00000000
148	559.72660000	61.62840000	0.00000000
149	560.89370000	12.32620000	0.00000000
150	562.36200000	6.62970000	0.00000000
151	562.97940000	13.68970000	0.00000000
152	563.85300000	7.95590000	0.00000000
153	564.26210000	8.01040000	0.00000000
154	569.50340000	0.47530000	0.00000000
155	570.20070000	1.43380000	0.00000000
156	573.67990000	1.04410000	0.00000000
157	581.28740000	35.00920000	0.00000000
158	586.08160000	10.45770000	0.00000000
159	586.84660000	64.37730000	0.00000000
160	588.33960000	17.18360000	0.00000000
161	588.86690000	18.30520000	0.00000000
162	594.22250000	4.81050000	0.00000000
163	596.21300000	4.03500000	0.00000000
164	596.72780000	1.57390000	0.00000000
165	597.26030000	5.39880000	0.00000000
166	613.22410000	4.37200000	0.00000000
167	622.66950000	1.98700000	0.00000000
168	637.74670000	5.36090000	0.00000000
169	644.41620000	0.82560000	0.00000000
170	647.67420000	445.24120000	0.00000000
171	648.09290000	0.05870000	0.00000000
172	649.85660000	139.61900000	0.00000000
173	651.52600000	794.28330000	0.00000000
174	662.77270000	5.16310000	0.00000000
175	664.06840000	2.82530000	0.00000000
176	667.22510000	182.27730000	0.00000000
177	670.55650000	155.71490000	0.00000000
178	671.70170000	55.75130000	0.00000000
179	677.30490000	22.59530000	0.00000000
180	678.50550000	61.15360000	0.00000000
181	679.45860000	34.93100000	0.00000000
182	686.40610000	701.52290000	0.00000000
183	686.93780000	30.24410000	0.00000000
184	688.20360000	155.82330000	0.00000000
185	716.53790000	104.18890000	0.00000000
186	721.92010000	3.01730000	0.00000000
187	729.32000000	592.34050000	0.00000000
188	732.35340000	24.73100000	0.00000000

189	738.41600000	515.19060000	0.00000000
190	744.75790000	18.04320000	0.00000000
191	749.68500000	217.30880000	0.00000000
192	750.80590000	11.41550000	0.00000000
193	753.50030000	3.56320000	0.00000000
194	754.15070000	1.67440000	0.00000000
195	754.24220000	0.08180000	0.00000000
196	758.45400000	95.82830000	0.00000000
197	761.06550000	100.80250000	0.00000000
198	765.03680000	54.52000000	0.00000000
199	766.43520000	2.02520000	0.00000000
200	767.51090000	42.29870000	0.00000000
201	768.80670000	34.71440000	0.00000000
202	770.81730000	33.35650000	0.00000000
203	771.52160000	9.12380000	0.00000000
204	772.92350000	4.39540000	0.00000000
205	774.44930000	2.42270000	0.00000000
206	799.66250000	1.19540000	0.00000000
207	808.93790000	42.70030000	0.00000000
208	820.71010000	20.24250000	0.00000000
209	822.84960000	0.70900000	0.00000000
210	827.26150000	3.15070000	0.00000000
211	831.02280000	1.29340000	0.00000000
212	831.96130000	0.11560000	0.00000000
213	846.20110000	131.82990000	0.00000000
214	849.54970000	9.56930000	0.00000000
215	850.62910000	0.63990000	0.00000000
216	855.16810000	1.07040000	0.00000000
217	856.12670000	0.08130000	0.00000000
218	863.05310000	2.61210000	0.00000000
219	870.19590000	18.01550000	0.00000000
220	871.22290000	25.42110000	0.00000000
221	871.60600000	23.43760000	0.00000000
222	875.02900000	53.21770000	0.00000000
223	881.02490000	1.71950000	0.00000000
224	882.93870000	0.93580000	0.00000000
225	883.63740000	1.44080000	0.00000000
226	884.29150000	0.82670000	0.00000000
227	903.20960000	61.13250000	0.00000000
228	912.50590000	1332.57120000	0.00000000
229	921.32510000	73.43370000	0.00000000
230	921.98850000	75.28370000	0.00000000
231	922.98590000	39.76390000	0.00000000
232	923.98430000	17.97700000	0.00000000
233	926.11710000	2.08610000	0.00000000
234	927.28560000	178.80260000	0.00000000
235	928.06500000	4.69410000	0.00000000
236	949.49840000	2.50060000	0.00000000
237	950.86310000	2.12240000	0.00000000
238	951.37920000	3.63770000	0.00000000
239	952.21740000	1.27220000	0.00000000
240	954.01150000	11.96600000	0.00000000
241	957.88900000	0.34090000	0.00000000
242	990.13350000	1799.74980000	0.00000000
243	994.28660000	0.07310000	0.00000000
244	995.24570000	0.05430000	0.00000000
245	997.69580000	7.81270000	0.00000000
246	999.38160000	0.29170000	0.00000000
247	999.76840000	0.25900000	0.00000000
248	1000.84390000	2.16950000	0.00000000
249	1001.77030000	0.44220000	0.00000000
250	1002.28540000	0.25620000	0.00000000
251	1002.72180000	0.25540000	0.00000000
252	1003.94210000	0.64350000	0.00000000

253	1051.53370000	5.39990000	0.00000000
254	1054.32720000	9.87370000	0.00000000
255	1058.14100000	0.92140000	0.00000000
256	1059.31760000	0.18850000	0.00000000
257	1064.79560000	14.94150000	0.00000000
258	1065.24140000	11.88270000	0.00000000
259	1067.29690000	1.05130000	0.00000000
260	1070.72150000	6.55460000	0.00000000
261	1077.24040000	2.92050000	0.00000000
262	1079.16200000	0.68290000	0.00000000
263	1079.57220000	0.03070000	0.00000000
264	1079.92370000	2.09570000	0.00000000
265	1080.56740000	0.60470000	0.00000000
266	1081.57400000	0.90180000	0.00000000
267	1083.63810000	8.71320000	0.00000000
268	1084.55420000	1.76070000	0.00000000
269	1088.57590000	3.14720000	0.00000000
270	1090.18860000	4.79710000	0.00000000
271	1092.02850000	105.36740000	0.00000000
272	1101.79510000	75.20850000	0.00000000
273	1112.29670000	11.15590000	0.00000000
274	1120.54480000	506.32840000	0.00000000
275	1126.65770000	74.11880000	0.00000000
276	1129.15310000	471.46900000	0.00000000
277	1129.79070000	32.08920000	0.00000000
278	1130.82390000	58.43010000	0.00000000
279	1132.44760000	151.32670000	0.00000000
280	1135.26730000	15.03780000	0.00000000
281	1138.08920000	5.94460000	0.00000000
282	1139.70580000	133.02420000	0.00000000
283	1141.78720000	18.82510000	0.00000000
284	1144.36430000	18.84230000	0.00000000
285	1155.33540000	62.61120000	0.00000000
286	1157.50260000	1013.00340000	0.00000000
287	1158.79520000	101.62270000	0.00000000
288	1168.42430000	29.23080000	0.00000000
289	1172.80320000	7.51930000	0.00000000
290	1184.02140000	187.96160000	0.00000000
291	1196.74990000	0.13880000	0.00000000
292	1198.39760000	0.12600000	0.00000000
293	1201.51450000	4.48260000	0.00000000
294	1218.38570000	0.79930000	0.00000000
295	1223.55980000	4.27810000	0.00000000
296	1232.60570000	5.80510000	0.00000000
297	1237.33130000	7.06620000	0.00000000
298	1237.40360000	3.79710000	0.00000000
299	1238.85730000	0.65810000	0.00000000
300	1243.18120000	13.45220000	0.00000000
301	1255.93660000	2.29380000	0.00000000
302	1262.79150000	6.77520000	0.00000000
303	1263.30900000	16.18480000	0.00000000
304	1263.77380000	33.35860000	0.00000000
305	1264.91190000	36.71090000	0.00000000
306	1265.16460000	11.28320000	0.00000000
307	1265.39920000	21.42400000	0.00000000
308	1266.24530000	3.65870000	0.00000000
309	1271.54870000	52.58450000	0.00000000
310	1279.38670000	87.13970000	0.00000000
311	1291.79530000	7.17930000	0.00000000
312	1311.38040000	9.60090000	0.00000000
313	1313.48460000	155.06400000	0.00000000
314	1315.23640000	64.95840000	0.00000000
315	1315.36410000	86.93960000	0.00000000
316	1316.25810000	77.07460000	0.00000000

317	1316.34980000	19.24750000	0.00000000
318	1316.81830000	25.81670000	0.00000000
319	1321.02810000	4.91130000	0.00000000
320	1323.89650000	1.60040000	0.00000000
321	1329.00940000	43.86580000	0.00000000
322	1342.34820000	0.81200000	0.00000000
323	1345.01550000	0.09640000	0.00000000
324	1356.16180000	8.78930000	0.00000000
325	1361.75890000	0.79440000	0.00000000
326	1364.61810000	51.39000000	0.00000000
327	1377.29260000	4.01050000	0.00000000
328	1378.84310000	0.72590000	0.00000000
329	1379.42710000	0.18260000	0.00000000
330	1391.25770000	217.36600000	0.00000000
331	1397.18530000	22.64720000	0.00000000
332	1400.17120000	315.14280000	0.00000000
333	1420.81860000	108.82290000	0.00000000
334	1423.02430000	73.13520000	0.00000000
335	1423.08720000	61.36500000	0.00000000
336	1428.37630000	110.36830000	0.00000000
337	1430.77730000	36.84460000	0.00000000
338	1431.31270000	20.69660000	0.00000000
339	1431.57330000	62.75610000	0.00000000
340	1434.71990000	583.74330000	0.00000000
341	1440.63120000	214.35800000	0.00000000
342	1445.69410000	109.01440000	0.00000000
343	1447.11730000	38.42230000	0.00000000
344	1447.18750000	36.57860000	0.00000000
345	1459.95950000	1.49400000	0.00000000
346	1470.24170000	103.65040000	0.00000000
347	1472.87380000	0.60360000	0.00000000
348	1474.30280000	883.12920000	0.00000000
349	1478.67460000	78.35200000	0.00000000
350	1488.96160000	2.88660000	0.00000000
351	1493.96480000	27.50820000	0.00000000
352	1494.06980000	2.26600000	0.00000000
353	1494.22160000	9.35460000	0.00000000
354	1497.81890000	189.11860000	0.00000000
355	1501.45430000	25.84820000	0.00000000
356	1511.49900000	6.17860000	0.00000000
357	1522.09460000	201.18060000	0.00000000
358	1524.31420000	12.78500000	0.00000000
359	1524.68820000	239.01010000	0.00000000
360	1525.07970000	128.07820000	0.00000000
361	1526.99570000	206.65780000	0.00000000
362	1536.52040000	22.21670000	0.00000000
363	1537.20770000	298.81590000	0.00000000
364	1538.10650000	162.66500000	0.00000000
365	1543.23360000	40.22910000	0.00000000
366	1547.35770000	0.04690000	0.00000000
367	1551.45650000	20.14250000	0.00000000
368	1571.45060000	73.27630000	0.00000000
369	1585.57170000	13.85220000	0.00000000
370	1585.82780000	10.82130000	0.00000000
371	1587.21230000	22.49720000	0.00000000
372	1587.65440000	58.84520000	0.00000000
373	1588.20220000	19.10010000	0.00000000
374	1590.68930000	2.19610000	0.00000000
375	1593.03680000	29.82230000	0.00000000
376	1603.20210000	38.83620000	0.00000000
377	1612.73800000	7.88800000	0.00000000
378	1613.10760000	71.13750000	0.00000000
379	1613.96810000	41.84480000	0.00000000
380	1618.67090000	21.72300000	0.00000000

381	1620.41260000	0.62530000	0.00000000
382	1621.71420000	29.17260000	0.00000000
383	1622.64620000	30.85420000	0.00000000
384	1625.85890000	0.67060000	0.00000000
385	1626.26370000	0.03550000	0.00000000
386	1632.68400000	4.85470000	0.00000000
387	1638.33940000	13.35620000	0.00000000
388	1647.40610000	38.69310000	0.00000000
389	1647.94040000	32.87350000	0.00000000
390	1658.53770000	161.82040000	0.00000000
391	1659.50880000	13.86690000	0.00000000
392	1659.56170000	340.60580000	0.00000000
393	1659.99330000	3.29190000	0.00000000
394	1660.23630000	15.94640000	0.00000000
395	1660.84980000	53.38890000	0.00000000
396	1661.46810000	70.01690000	0.00000000
397	1662.67620000	70.06380000	0.00000000
398	1663.52480000	3.58260000	0.00000000
399	1664.92710000	105.05010000	0.00000000
400	1666.95650000	134.73310000	0.00000000
401	3157.48240000	165.35510000	0.00000000
402	3158.66940000	206.17430000	0.00000000
403	3166.40670000	159.22320000	0.00000000
404	3167.47230000	132.03600000	0.00000000
405	3190.84530000	6.72290000	0.00000000
406	3192.63180000	45.38350000	0.00000000
407	3193.17430000	35.38250000	0.00000000
408	3193.67110000	35.65330000	0.00000000
409	3197.21350000	5.90560000	0.00000000
410	3197.38410000	5.84300000	0.00000000
411	3198.42330000	5.70700000	0.00000000
412	3198.69750000	4.86200000	0.00000000
413	3201.19650000	21.86220000	0.00000000
414	3201.26960000	0.90600000	0.00000000
415	3202.30300000	9.38430000	0.00000000
416	3202.72940000	2.71310000	0.00000000
417	3202.84960000	9.48180000	0.00000000
418	3202.85300000	2.14690000	0.00000000
419	3203.90090000	30.87100000	0.00000000
420	3204.72390000	14.53250000	0.00000000
421	3213.97720000	1.61910000	0.00000000
422	3214.19280000	2.19850000	0.00000000
423	3217.38070000	3.01230000	0.00000000
424	3218.77700000	3.95070000	0.00000000
425	3224.85810000	20.50320000	0.00000000
426	3228.61150000	14.49130000	0.00000000
427	3229.47260000	5.61910000	0.00000000
428	3230.16620000	0.25550000	0.00000000
429	3230.17690000	7.76790000	0.00000000
430	3233.91570000	6.96590000	0.00000000
431	3234.51410000	3.34990000	0.00000000
432	3235.77520000	2.97200000	0.00000000
433	3237.29480000	3.11210000	0.00000000
434	3238.42690000	4.56110000	0.00000000
435	3238.76460000	5.21210000	0.00000000
436	3239.11490000	1.38040000	0.00000000
437	3520.50270000	1737.29320000	0.00000000
438	3540.24760000	1798.12790000	0.00000000
439	3616.46410000	589.85900000	0.00000000
440	3616.60700000	467.14290000	0.00000000
441	3617.47060000	1582.81630000	0.00000000
442	3618.42380000	982.33870000	0.00000000
443	3647.13990000	179.08280000	0.00000000
444	3648.02050000	1262.88310000	0.00000000

445	3800.02730000	41.87060000	0.00000000
446	3800.12430000	36.91690000	0.00000000
447	3846.72340000	70.59170000	0.00000000
448	3847.09280000	72.44790000	0.00000000
449	3847.31670000	98.83760000	0.00000000
450	3847.45120000	131.80980000	0.00000000
451	3847.58620000	8.25810000	0.00000000
452	3847.59460000	105.09240000	0.00000000
453	3847.69320000	171.10050000	0.00000000
454	3848.52960000	94.21260000	0.00000000
455	3891.22810000	90.24350000	0.00000000
456	3892.24190000	90.10970000	0.00000000

S12. CALCULATIONS ON 2^{4+} (^{1}A) AT ^{3}A STRUCTURE IN WATER (EXPLICIT + PCM) (SINGLE POINT)

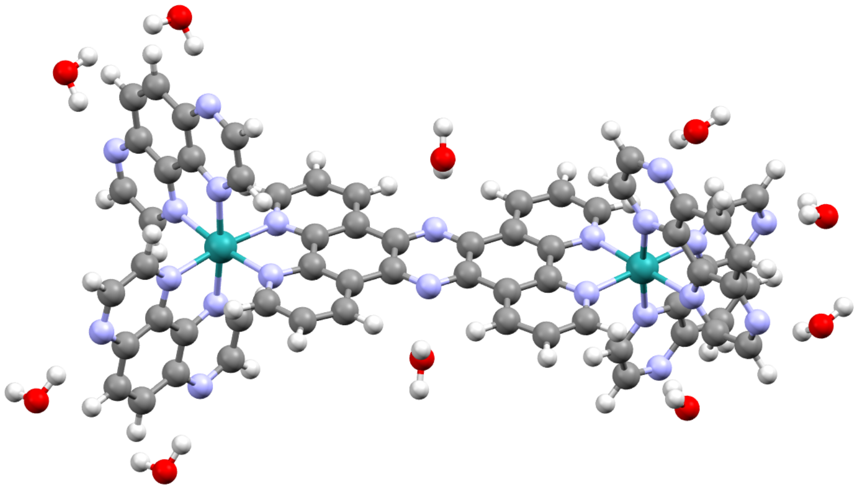

Route	: # b3lyp/genecp scrf=(solvent=water) geom=connectivity empiricaldispers
	: ion=gd3bj int=ultrafine pop=regular
SMILES	:
Formula	: $C_{64}H_{56}N_{22}O_{10}Ru_2^{4+}$
Charge	: 4
Multiplicity	: 1
Energy	: -4620.60511773 a.u.

S12.1. Cartesian Co-ordinates (XYZ format)

154

Ru	6.45210886	0.01507200	-0.00355500
N	4.84286499	0.12706199	-1.33341002
N	0.03033600	0.07950000	-1.41216195
N	0.02214200	-0.11628200	1.37125897
N	4.83709717	-0.11477100	1.31860495
C	4.89455080	0.23560500	-2.66462803
H	5.87750292	0.29004201	-3.10941195
C	3.74307203	0.28316799	-3.45126200
H	3.84277511	0.37150699	-4.52435303
C	2.49734807	0.21948200	-2.85408807
H	1.58913803	0.25675499	-3.44678092
C	2.42928100	0.10585300	-1.45643401
C	1.17058396	0.03832500	-0.72741401
C	-1.11348295	0.02606100	-0.73394501
C	-3.56589699	0.01755200	-0.75171500
C	-3.57007694	-0.05825500	0.68449402
C	-2.37818193	-0.11327300	1.41518295
C	-1.11786497	-0.06940200	0.68583697
C	1.16661096	-0.06626600	0.69350302
C	2.42235804	-0.12164500	1.42903900
C	2.48473191	-0.23486300	2.82698488
H	1.57381201	-0.28344700	3.41482711

C	3.72810102	-0.28379801	3.43058896
H	3.82321000	-0.37123501	4.50417805
C	4.88314009	-0.22214700	2.65011692
H	5.86452198	-0.26438099	3.09975195
C	3.62243104	-0.06081800	0.70852602
C	3.62582994	0.05847300	-0.72968698
C	7.46537590	-2.35834002	-1.31319201
C	5.87985706	-3.03967595	0.19783400
C	7.68294382	-3.68534589	-1.72274399
C	8.20028114	-1.30009198	-1.90177798
C	6.10042286	-4.36749315	-0.22208500
H	5.15972424	-2.80721593	0.96858603
C	9.15818119	-1.56955898	-2.89505196
H	5.53194380	-5.16826010	0.23495600
C	8.64182377	0.94681501	-2.02654505
C	9.60453701	0.66801602	-3.01845193
H	8.45146942	1.95926201	-1.70161402
H	10.16533375	1.48487794	-3.45602393
N	7.93737602	-0.03354500	-1.47260201
N	6.56104183	-2.03718495	-0.34541801
C	8.18505573	1.34948599	1.89525497
C	8.64708233	-0.89299703	2.02445292
C	9.14196301	1.62998998	2.88641405
C	7.44039679	2.39985490	1.30475795
C	9.60837746	-0.60313499	3.01456690
H	8.46588898	-1.90782404	1.70156300
C	7.64699697	3.72982001	1.71044302
H	10.17628860	-1.41401994	3.45408392
C	5.84788322	3.06363702	-0.20672700
C	6.05679607	4.39428616	0.21003100
H	5.12929392	2.82315207	-0.97643298
H	5.48067522	5.18886089	-0.24824600
N	7.93337107	0.07971100	1.46889198
N	6.53876305	2.06851292	0.33789000
C	-4.83650923	0.12751999	-2.69688797
C	-4.85895109	-0.17969701	2.62334394
C	-2.36775994	0.06641500	-1.47281206
C	-2.43683600	0.15051199	-2.87280297
H	-1.52830803	0.19388101	-3.46564102
C	-2.45971608	-0.20551901	2.81417990
H	-1.55578804	-0.25287101	3.41396308
C	-3.68145394	0.18055300	-3.47700691
C	-3.70933199	-0.24026901	3.40955591
H	-3.77697492	0.24472900	-4.55196810
H	-5.81914091	0.14428400	-3.14563394
H	-3.81173205	-0.31432801	4.48321915
H	-5.84798908	-0.20147499	3.05707502
N	-4.78036308	0.04327200	-1.36426699
N	-4.78834009	-0.08149000	1.29110503
Ru	-6.39388418	-0.01053700	-0.02241400
N	-7.98725891	0.16365600	-1.39683795
N	-6.49348783	2.09266591	-0.26141900
N	-7.89415216	-0.17615700	1.41447401
N	-6.48762989	-2.06714106	0.13064000
C	-8.27418041	1.45620501	-1.70427001
C	-8.74343967	-0.78313202	-1.93351305
C	-7.48230219	2.47025108	-1.11492205
C	-5.74430609	3.04507709	0.27512699
C	-8.17120171	-1.49951100	1.70845604
C	-8.65277672	0.75378197	2.01861191
C	-7.43373203	-2.47589493	1.05869901
C	-5.73307180	-3.03509402	-0.43585199
C	-9.32289505	1.79386199	-2.57816291
C	-9.79873371	-0.43329200	-2.80526900

H	-8.52418423	-1.81315100	-1.69222903
C	-7.72391987	3.82249594	-1.41673195
C	-5.99147606	4.40022087	-0.03687600
H	-4.95528793	2.75818801	0.95496798
C	-9.18481445	-1.84145105	2.64468193
C	-9.64247990	0.39005601	2.92127299
H	-8.45503426	1.79257500	1.79459798
C	-7.62718391	-3.85284710	1.34396899
C	-5.94250202	-4.36608601	-0.13205400
H	-4.97996902	-2.73278308	-1.14912701
H	-10.40820980	-1.21659005	-3.23833609
H	-5.37298298	5.16788101	0.41115099
H	-10.23331642	1.15505302	3.40671897
H	-5.34037590	-5.12682009	-0.61027497
C	9.34239292	2.99173188	3.29153299
H	10.08525753	3.17548394	4.05764484
C	8.62293530	4.00136900	2.72659492
H	8.75710297	5.03514290	3.01948905
C	8.65894985	-3.94560003	-2.74183702
H	8.80049419	-4.97722197	-3.03883410
C	9.36954594	-2.92836905	-3.30438805
H	10.11010838	-3.10317111	-4.07481003
C	-8.64972591	-4.19716311	2.30246091
H	-8.79556465	-5.25116682	2.50392008
C	-9.39382076	-3.24076796	2.91896009
H	-10.16724682	-3.49113011	3.63431692
C	-8.79107857	4.15312481	-2.31622910
H	-8.95140839	5.20303917	-2.52761698
C	-9.56227779	3.17705393	-2.87283897
H	-10.37444305	3.40385604	-3.55228996
N	-6.95218611	4.78418016	-0.85570103
N	-10.08329487	0.81482899	-3.12449288
N	6.93077707	4.73003006	1.14178801
N	9.85541439	0.62137097	3.44336295
N	9.86158752	-0.55319798	-3.45072889
N	6.97606993	-4.69318819	-1.15584695
N	-9.91022491	-0.89659399	3.24376702
N	-6.88986778	-4.79041290	0.74722803
O	-0.01487600	-0.42133799	4.89435387
O	0.01705300	0.34692600	-4.94518900
H	0.02607200	0.24491499	5.58945417
H	0.00284900	-1.26373601	5.36214781
H	0.05781500	-0.36321399	-5.59544086
H	0.01792000	1.15674698	-5.46767998
O	11.72029972	-1.79622197	-5.33273792
O	7.96470308	-7.15511608	-2.38408208
O	7.90007401	7.20405006	2.36095810
O	11.53895760	1.86403203	5.48429823
O	-12.11053276	2.23151803	-4.72119617
O	-8.08572006	7.32369280	-1.81805003
O	-11.68532276	-2.21572208	5.09142494
O	-7.69729614	-7.34356308	1.78436601
H	-11.07223988	-2.35599899	5.82102823
H	-7.19620609	-7.34898376	2.60696507
H	-12.75242233	2.42482805	-4.02904177
H	-8.79482174	7.43696213	-1.17555106
H	8.74129486	-7.30937815	-1.83518302
H	10.89750767	1.91134405	6.20164824
H	8.67480469	7.36358309	1.81093502
H	12.42304897	-2.04378390	-4.72195482
H	7.42905807	6.49714804	1.88518500
H	11.15488434	-1.21096206	-4.79825592
H	7.48798323	-6.45365620	-1.90594494
H	11.10163116	1.28950000	4.83123398

H	-7.54215717	6.61671400	-1.43093503
H	-11.51939297	1.58197904	-4.30434418
H	-7.34972286	-6.55799484	1.31677198
H	-11.18245125	-1.63598597	4.48657179

S13. CALCULATIONS ON 2^{3+} (^{2}A) AT 2^{4+} (^{1}A) STRUCTURE IN MeCN (SINGLE POINT)

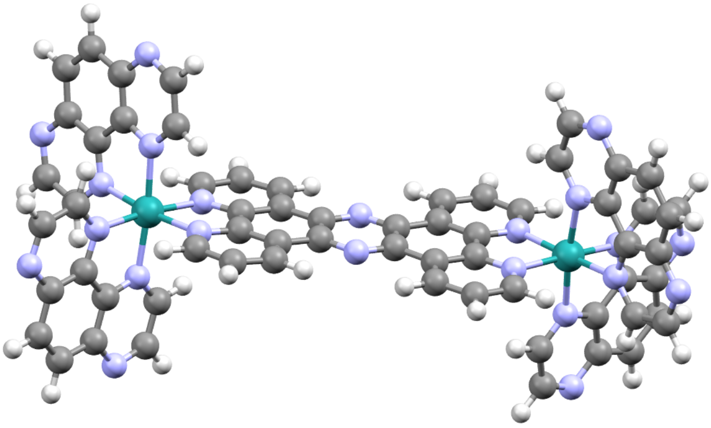

Route	: # b3lyp/genecp scrf=(solvent=acetonitrile) geom=connectivity empirical
	: dispersion=gd3bj int=ultrafine pop=regular
SMILES	:
Formula	: $C_{64}H_{36}N_{22}Ru_2^{3+,2}$
Charge	: 3
Multiplicity	: 2
Energy	: -3856.05908712 a.u.

S13.1. Cartesian Co-ordinates (XYZ format)

124

Ru	6.42851686	-0.00000300	0.00001500
N	4.81623602	-0.06660500	1.33004606
N	0.00000300	-0.00001800	1.39570904
N	0.00000800	-0.00025400	-1.39570904
N	4.81623888	0.06649500	-1.33002603
C	4.86450291	-0.11472600	2.66459298
H	5.84633589	-0.14699100	3.11409092
C	3.71109605	-0.12844600	3.45091796
H	3.80778790	-0.16880600	4.52687597
C	2.47046208	-0.09181800	2.84361291
H	1.55614305	-0.10311600	3.42039108
C	2.40106606	-0.04148200	1.44385397
C	1.14257002	-0.00929500	0.71174401
C	-1.14256203	0.00914900	0.71173799
C	-3.60060096	0.02712300	0.72063202
C	-3.60059810	-0.02729600	-0.72064698
C	-2.40105605	-0.04172300	-1.44384694
C	-1.14255905	-0.00941600	-0.71174198
C	1.14257205	0.00902800	-0.71173900
C	2.40107107	0.04125600	-1.44384503
C	2.47047091	0.09159600	-2.84360290
H	1.55615401	0.10285600	-3.42038488
C	3.71110606	0.12827800	-3.45090294

H	3.80780101	0.16864499	-4.52686024
C	4.86451006	0.11461600	-2.66457200
H	5.84634304	0.14693300	-3.11406493
C	3.60061002	0.02700600	-0.72063702
C	3.60060811	-0.02717900	0.72065097
C	7.43644810	2.42020392	1.22885597
C	5.85082197	3.04681301	-0.30356601
C	7.65084124	3.76316905	1.58826602
C	8.17425919	1.38370299	1.85246694
C	6.07234716	4.38870621	0.07101800
H	5.13056183	2.78723907	-1.06567895
C	9.13587666	1.68479300	2.83417797
H	5.50071096	5.17024279	-0.41603300
C	8.62268925	-0.85556799	2.05048490
C	9.58779907	-0.53836298	3.02946091
H	8.43495274	-1.87899494	1.75909805
H	10.15091991	-1.34166098	3.49024892
N	7.91292906	0.10294100	1.46604896
N	6.53213978	2.06381893	0.27320901
C	8.17436314	-1.38358998	-1.85242701
C	8.62263584	0.85571301	-2.05044794
C	9.13600731	-1.68461502	-2.83413291
C	7.43662119	-2.42014194	-1.22881997
C	9.58777237	0.53857303	-3.02942109
H	8.43482685	1.87912703	-1.75906503
C	7.65111017	-3.76309204	-1.58822596
H	10.15083694	1.34190905	-3.49020910
C	5.85102892	-3.04685903	0.30359301
C	6.07264996	-4.38873816	-0.07098700
H	5.13074589	-2.78733492	1.06570196
H	5.50106478	-5.17031384	0.41606200
N	7.91294193	-0.10284500	-1.46601295
N	6.53228092	-2.06381798	-0.27317899
C	-4.86449814	0.11504300	2.66455507
C	-4.86449099	-0.11515300	-2.66457605
C	-2.40106106	0.04149600	1.44383800
C	-2.47045898	0.09206500	2.84358811
H	-1.55614102	0.10342300	3.42036700
C	-2.47044992	-0.09228700	-2.84359789
H	-1.55613005	-0.10368300	-3.42037296
C	-3.71109295	0.12884000	3.45088291
C	-3.71108294	-0.12900700	-3.45089793
H	-3.80778694	0.16937999	4.52683306
H	-5.84633017	0.14742400	3.11404610
H	-3.80777407	-0.16954100	-4.52684879
H	-5.84632301	-0.14748199	-3.11407089
N	-4.81622791	0.06670500	1.33001697
N	-4.81622601	-0.06681600	-1.33003700
Ru	-6.42850494	-0.00000300	-0.00001500
N	-7.91293192	-0.10262800	1.46603000
N	-6.53227377	-2.06377697	0.27348399
N	-7.91292000	0.10272300	-1.46606696
N	-6.53213310	2.06377697	-0.27351400
C	-8.17437458	-1.38331699	1.85261202
C	-8.62260723	0.85601801	2.05034494
C	-7.43663597	-2.41996193	1.22915399
C	-5.85101223	-3.04690099	-0.30313399
C	-8.17427063	1.38343000	-1.85265100
C	-8.62265968	-0.85587400	-2.05038095
C	-7.43646383	2.42002392	-1.22919095
C	-5.85080481	3.04685497	0.30310699
C	-9.13602829	-1.68419695	2.83435202
C	-9.58774471	0.53902298	3.02936101
H	-8.43478203	1.87939095	1.75882602

C	-7.65114403	-3.76286101	1.58873904
C	-6.07265186	-4.38872719	0.07162300
H	-5.13070583	-2.78748202	-1.06525803
C	-9.13589764	1.68437600	-2.83439708
C	-9.58777332	-0.53881299	-3.02940106
H	-8.43490887	-1.87925899	-1.75885904
C	-7.65087605	3.76293802	-1.58877897
C	-6.07235003	4.38869476	-0.07165400
H	-5.13052177	2.78738594	1.06523502
H	-10.15079117	1.34243095	3.49005008
H	-5.50106096	-5.17037392	-0.41530401
H	-10.15087509	-1.34218204	-3.49008989
H	-5.50070715	5.17030287	0.41527501
C	9.34327030	-3.05850792	-3.19172502
H	10.08540344	-3.27068090	-3.94998908
C	8.63014412	-4.05483580	-2.59564996
H	8.78080463	-5.09326315	-2.85971498
C	8.62984943	4.05497885	2.59569693
H	8.78043556	5.09341621	2.85976410
C	9.34304142	3.05870008	3.19177294
H	10.08515739	3.27092409	3.95004201
C	-8.62990475	4.05460215	-2.59623194
H	-8.78050995	5.09300089	-2.86043596
C	-9.34308910	3.05823302	-3.19216895
H	-10.08521461	3.27034497	-3.95045805
C	-8.63020039	-4.05445814	2.59618497
H	-8.78087902	-5.09284782	2.86038709
C	-9.34331703	-3.05804110	3.19212008
H	-10.08546162	-3.27010202	3.95040607
N	-6.94741917	-4.75382519	0.99184197
N	-9.84696007	-0.69454098	3.42464089
N	6.94738913	-4.75396919	-0.99117899
N	9.84695816	-0.69504702	-3.42454696
N	9.84689617	0.69527400	3.42459202
N	6.94705486	4.75399685	0.99121600
N	-9.84689808	0.69476801	-3.42468596
N	-6.94708490	4.75385284	-0.99187899

S14. CALCULATIONS ON 2^{3+} (^{2}A) IN MeCN

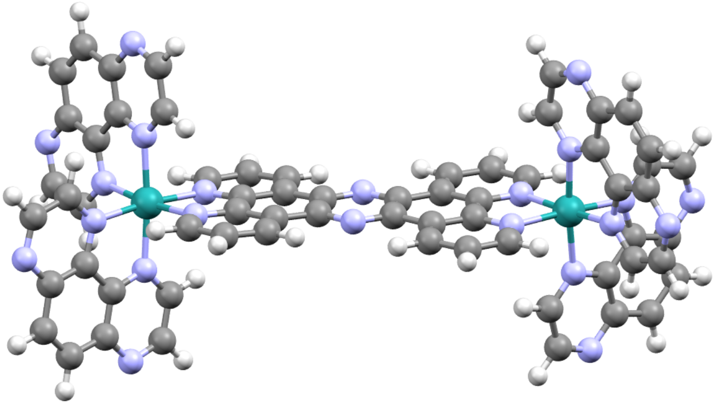

Route	: # opt freq b3lyp/genecp scrf=(solvent=acetonitrile) geom=connectivity	
	: empiricaldispersion=gd3bj int=ultrafine pop=regular	
SMILES	: c1cc2c3c(c4ccc[n+]5c4c2[n+](c1)[Ru]567([n+]8ccnc9c8c1[n+]6ccnc1cc9)[n+]1ccnc2c1c1[n+]7ccnc1cc2)nc1c2ccc[n+]4c2c2c(c1n3)ccc[n+]2[Ru]412([n+]3ccnc4c3c3[n+]1ccnc3cc4)[n+]1ccnc3c1c1[n+]2ccnc1cc3	
Formula	: $C_{64}H_{36}N_{22}Ru_2^{3+,2}$	
Charge	: 3	
Multiplicity	: 2	
Energy	: -3856.06264446	a.u.
Gibbs Energy	: -3855.24191300	a.u.
Number of imaginary frequencies	: 2	

S14.1. Cartesian Co-ordinates (XYZ format)

124

Ru	6.44484091	0.00000400	0.00005000
N	4.83778095	-0.06668300	1.33466399
N	-0.00000700	0.00050600	1.42044902
N	0.00002000	0.00028700	-1.42044902
N	4.83781195	0.06700000	-1.33457994
C	4.88182020	-0.11967700	2.66391492
H	5.86098289	-0.15057200	3.12003899
C	3.71653390	-0.13962699	3.44479990
H	3.80752707	-0.18497400	4.52146387
C	2.48286009	-0.10167100	2.83123803
H	1.56552899	-0.11671000	3.40335298
C	2.40521908	-0.04500600	1.42525899
C	1.15003395	-0.00932400	0.70856702
C	-1.15003300	0.01022500	0.70854402
C	-3.62319207	0.02838100	0.71078300
C	-3.62317705	-0.02788700	-0.71084601
C	-2.40520501	-0.04522600	-1.42525101
C	-1.15002000	-0.00943400	-0.70856500

C	1.15004694	0.01011500	-0.70854503
C	2.40524793	0.04566800	-1.42521501
C	2.48291993	0.10232200	-2.83119297
H	1.56560099	0.11747800	-3.40332294
C	3.71660900	0.14011900	-3.44473505
H	3.80762601	0.18544801	-4.52139616
C	4.88187885	0.11999900	-2.66383004
H	5.86105490	0.15074199	-3.11993694
C	3.62320495	0.02827200	-0.71078700
C	3.62318993	-0.02777800	0.71085101
C	7.43202019	2.43024111	1.21966600
C	5.84333420	3.03361702	-0.31920400
C	7.63081408	3.77640510	1.57489896
C	8.17556095	1.40268004	1.85123801
C	6.04930305	4.37870979	0.05147000
H	5.12693310	2.76013589	-1.08034301
C	9.13005543	1.71591198	2.83595300
H	5.46974277	5.15241623	-0.43887001
C	8.63964844	-0.83183098	2.06196094
C	9.59807205	-0.50277603	3.04290104
H	8.45900440	-1.85790098	1.77471304
H	10.16552734	-1.29942799	3.51006198
N	7.92496395	0.11832200	1.46921599
N	6.53528690	2.06022596	0.26199701
C	8.17529678	-1.40301001	-1.85113096
C	8.63983536	0.83140898	-2.06183195
C	9.12973404	-1.71642494	-2.83584309
C	7.43154812	-2.43042493	-1.21956801
C	9.59820366	0.50217003	-3.04276490
H	8.45939159	1.85751295	-1.77457702
C	7.63007689	-3.77662706	-1.57480705
H	10.16582203	1.29871297	-3.50991392
C	5.84273195	-3.03349090	0.31928799
C	6.04843807	-4.37862301	-0.05138900
H	5.12637997	-2.75987196	1.08042300
H	5.46871996	-5.15221691	0.43894300
N	7.92495394	-0.11860400	-1.46910095
N	6.53488398	-2.06023502	-0.26190099
C	-4.88186312	0.12040300	2.66381407
C	-4.88180399	-0.12008200	-2.66389894
C	-2.40523410	0.04588800	1.42520702
C	-2.48290396	0.10275600	2.83117700
H	-1.56558299	0.11800000	3.40330195
C	-2.48284411	-0.10210500	-2.83122206
H	-1.56551194	-0.11723200	-3.40333200
C	-3.71659207	0.14064300	3.44471407
C	-3.71651697	-0.14015201	-3.44478011
H	-3.80760789	0.18613701	4.52136898
H	-5.86103821	0.15121301	3.11991906
H	-3.80750895	-0.18566300	-4.52143621
H	-5.86096621	-0.15104300	-3.12001991
N	-4.83779812	0.06720300	1.33457196
N	-4.83776712	-0.06688600	-1.33465505
Ru	-6.44482613	0.00000400	-0.00005000
N	-7.92494011	-0.11837900	1.46912003
N	-6.53488016	-2.06019592	0.26220599
N	-7.92495012	0.11809700	-1.46923494
N	-6.53528214	2.06018710	-0.26230201
C	-8.17530823	-1.40272903	1.85132098
C	-8.63979912	0.83172703	2.06172800
C	-7.43156624	-2.43024206	1.21990705
C	-5.84272480	-3.03354096	-0.31883201
C	-8.17557144	1.40240002	-1.85142803
C	-8.63961220	-0.83214802	-2.06185699

C	-7.43203783	2.43005800	-1.22000504
C	-5.84332705	3.03366709	0.31874800
C	-9.12975502	-1.71599495	2.83607006
C	-9.59817219	0.50263798	3.04270697
H	-8.45933819	1.85778797	1.77433300
C	-7.63011885	-3.77639198	1.57532895
C	-6.04845619	-4.37861919	0.05202700
H	-5.12635088	-2.76003408	-1.07998705
C	-9.13007641	1.71548200	-2.83617997
C	-9.59803963	-0.50324303	-3.04284406
H	-8.45895004	-1.85817599	-1.77446997
C	-7.63085604	3.77617097	-1.57542098
C	-6.04932117	4.37870598	-0.05210800
H	-5.12690401	2.76029897	1.07990599
H	-10.16576862	1.29925501	3.50975490
H	-5.46873713	-5.15228796	-0.43818501
H	-10.16547394	-1.29997003	-3.50990200
H	-5.46975899	5.15248823	0.43811199
C	9.32243633	-3.09400606	-3.18834496
H	10.05894947	-3.31655908	-3.94918299
C	8.60232067	-4.08123207	-2.58531594
H	8.74185944	-5.12198114	-2.84659410
C	8.60311508	4.08082294	2.58541012
H	8.74285793	5.12154484	2.84668398
C	9.32302856	3.09345698	3.18844795
H	10.05958080	3.31586790	3.94929099
C	-8.60317612	4.08043623	-2.58595800
H	-8.74294090	5.12111998	-2.84737110
C	-9.32307911	3.09297609	-3.18885708
H	-10.05964184	3.31527090	-3.94972301
C	-8.60238266	-4.08084488	2.58586407
H	-8.74194241	-5.12155581	2.84728193
C	-9.32248592	-3.09352493	3.18875408
H	-10.05900955	-3.31596303	3.94961500
N	-6.91748810	-4.75775290	0.97288299
N	-9.84644032	-0.73508102	3.43407607
N	6.91744423	-4.75789499	-0.97221202
N	9.84644222	-0.73560500	-3.43397593
N	9.84655762	0.73495299	3.43410206
N	6.91837883	4.75781298	0.97229600
N	-9.84655666	0.73442900	-3.43420291
N	-6.91842318	4.75767088	-0.97296602

S14.2. Frequencies

Mode	IR frequency	IR intensity	Raman intensity
1	-8.14590000	0.00000000	0.00000000
2	-6.50670000	0.06440000	0.00000000
3	9.46650000	0.00220000	0.00000000
4	23.81410000	0.00000000	0.00000000
5	24.18950000	0.00360000	0.00000000
6	26.94130000	0.04500000	0.00000000
7	28.78380000	0.22690000	0.00000000
8	38.34490000	1.00760000	0.00000000
9	39.78850000	0.18860000	0.00000000
10	42.61470000	0.00000000	0.00000000
11	43.75020000	0.39420000	0.00000000
12	45.21000000	0.19040000	0.00000000
13	49.36260000	1.27090000	0.00000000
14	53.24070000	0.00000000	0.00000000
15	63.62350000	5.03390000	0.00000000
16	72.99410000	2.18180000	0.00000000
17	79.46000000	0.00000000	0.00000000
18	84.25480000	9.71980000	0.00000000
19	90.83610000	4.29760000	0.00000000
20	92.48750000	0.30400000	0.00000000
21	96.54340000	0.14670000	0.00000000
22	104.79370000	0.00000000	0.00000000
23	132.34540000	0.01110000	0.00000000
24	146.05920000	0.60440000	0.00000000
25	158.28100000	2.30390000	0.00000000
26	168.25330000	3.14630000	0.00000000
27	172.19840000	2.17920000	0.00000000
28	173.52990000	0.80190000	0.00000000
29	174.77910000	0.00000000	0.00000000
30	177.60870000	0.59690000	0.00000000
31	180.21160000	0.00000000	0.00000000
32	182.31810000	2.68360000	0.00000000
33	187.13850000	0.00620000	0.00000000
34	188.77580000	0.01510000	0.00000000
35	190.52070000	0.00000000	0.00000000
36	193.34430000	0.09980000	0.00000000
37	194.31260000	0.37750000	0.00000000
38	196.16710000	4.04150000	0.00000000
39	207.13740000	0.00000000	0.00000000
40	211.39550000	0.01780000	0.00000000
41	213.96870000	0.59440000	0.00000000
42	223.86580000	0.00130000	0.00000000
43	224.43790000	18.72440000	0.00000000
44	226.30100000	26.38150000	0.00000000
45	226.81130000	0.20960000	0.00000000
46	230.80950000	7.12620000	0.00000000
47	234.48190000	0.06210000	0.00000000
48	243.92490000	0.00000000	0.00000000
49	286.41210000	8.30130000	0.00000000
50	286.70180000	10.72610000	0.00000000
51	286.77440000	22.89630000	0.00000000
52	286.80780000	0.05330000	0.00000000
53	293.94530000	4.21150000	0.00000000
54	294.29020000	2.41870000	0.00000000
55	297.88230000	0.00730000	0.00000000
56	297.89560000	2.84560000	0.00000000
57	302.70260000	0.00000000	0.00000000
58	319.15190000	6.00720000	0.00000000
59	319.91160000	17.20660000	0.00000000
60	325.44520000	0.89480000	0.00000000

61	338.00660000	0.00000000	0.00000000
62	338.71020000	4.34730000	0.00000000
63	345.27060000	1.22390000	0.00000000
64	350.59630000	2.67730000	0.00000000
65	351.67930000	1.56820000	0.00000000
66	380.23450000	0.90980000	0.00000000
67	389.24050000	0.00000000	0.00000000
68	423.54260000	15.02030000	0.00000000
69	445.11210000	13.40910000	0.00000000
70	445.43840000	0.00070000	0.00000000
71	445.47640000	27.87310000	0.00000000
72	447.25310000	0.00080000	0.00000000
73	447.26920000	0.00210000	0.00000000
74	447.77140000	0.19020000	0.00000000
75	447.82640000	0.00000000	0.00000000
76	452.16860000	0.00980000	0.00000000
77	456.37030000	12.98400000	0.00000000
78	460.40730000	0.00000000	0.00000000
79	460.68020000	3.35240000	0.00000000
80	468.15940000	0.10480000	0.00000000
81	471.65680000	0.71860000	0.00000000
82	474.73850000	0.09320000	0.00000000
83	485.01080000	0.00010000	0.00000000
84	485.68080000	38.87660000	0.00000000
85	486.48260000	16.13740000	0.00000000
86	487.98970000	4.62920000	0.00000000
87	495.08330000	0.00000000	0.00000000
88	498.41660000	10.46440000	0.00000000
89	503.45980000	1.79770000	0.00000000
90	503.72640000	29.14650000	0.00000000
91	507.54920000	0.00000000	0.00000000
92	533.93500000	132.60550000	0.00000000
93	536.25160000	31.23990000	0.00000000
94	553.61330000	0.24750000	0.00000000
95	555.10890000	16.75530000	0.00000000
96	555.19810000	8.66690000	0.00000000
97	555.26080000	0.00010000	0.00000000
98	555.29790000	9.54260000	0.00000000
99	556.43630000	1.75490000	0.00000000
100	562.58560000	0.00000000	0.00000000
101	562.74530000	0.30620000	0.00000000
102	568.76390000	0.00000000	0.00000000
103	571.25370000	0.06660000	0.00000000
104	581.69940000	54.53680000	0.00000000
105	581.72210000	31.55930000	0.00000000
106	583.88620000	0.00100000	0.00000000
107	583.92690000	20.04780000	0.00000000
108	591.73090000	2.07570000	0.00000000
109	593.38940000	11.61580000	0.00000000
110	595.10480000	0.00940000	0.00000000
111	595.10550000	0.00500000	0.00000000
112	595.19500000	13.28930000	0.00000000
113	595.24520000	0.92300000	0.00000000
114	605.48530000	0.24930000	0.00000000
115	640.34440000	0.00000000	0.00000000
116	640.42470000	0.00010000	0.00000000
117	641.15780000	0.61970000	0.00000000
118	644.62800000	0.04840000	0.00000000
119	644.63380000	0.00020000	0.00000000
120	647.56650000	1.04490000	0.00000000
121	664.29780000	1.35760000	0.00000000
122	664.86780000	7.17440000	0.00000000
123	669.16340000	5.87230000	0.00000000
124	670.44760000	0.33880000	0.00000000

125	670.47920000	0.00000000	0.00000000
126	675.84830000	0.02150000	0.00000000
127	700.56190000	0.00000000	0.00000000
128	718.55790000	0.00000000	0.00000000
129	735.64380000	103.79520000	0.00000000
130	736.82270000	0.25060000	0.00000000
131	739.23580000	0.00000000	0.00000000
132	743.27290000	12.01640000	0.00000000
133	748.90580000	39.75110000	0.00000000
134	749.08710000	84.36220000	0.00000000
135	753.84380000	0.50210000	0.00000000
136	753.84470000	1.93270000	0.00000000
137	753.84570000	0.03060000	0.00000000
138	753.85410000	0.01100000	0.00000000
139	759.02840000	0.00050000	0.00000000
140	759.10700000	68.06630000	0.00000000
141	759.85570000	62.78290000	0.00000000
142	761.42960000	0.15460000	0.00000000
143	762.82430000	2.09620000	0.00000000
144	762.94920000	26.87320000	0.00000000
145	762.99610000	0.00730000	0.00000000
146	781.01550000	0.31850000	0.00000000
147	797.70780000	0.00000000	0.00000000
148	798.69650000	0.31880000	0.00000000
149	821.82930000	146.77640000	0.00000000
150	822.90420000	0.00020000	0.00000000
151	823.40080000	0.48190000	0.00000000
152	826.41270000	0.00010000	0.00000000
153	839.25780000	0.45030000	0.00000000
154	853.39070000	0.30270000	0.00000000
155	853.39250000	0.18670000	0.00000000
156	853.60950000	0.06790000	0.00000000
157	853.62220000	0.00050000	0.00000000
158	859.71030000	0.17260000	0.00000000
159	859.71290000	0.19520000	0.00000000
160	860.72920000	0.00640000	0.00000000
161	860.73690000	0.14880000	0.00000000
162	870.27560000	157.01440000	0.00000000
163	881.74250000	2.23290000	0.00000000
164	881.75010000	0.28730000	0.00000000
165	882.33800000	0.03510000	0.00000000
166	882.37540000	11.88740000	0.00000000
167	893.02920000	3.48560000	0.00000000
168	893.05010000	196.13110000	0.00000000
169	893.29080000	214.09540000	0.00000000
170	893.30640000	1.80540000	0.00000000
171	924.87550000	0.00240000	0.00000000
172	925.04470000	0.00030000	0.00000000
173	927.30690000	0.00000000	0.00000000
174	927.33640000	0.51680000	0.00000000
175	941.47210000	1.00480000	0.00000000
176	945.99830000	0.70700000	0.00000000
177	946.24210000	0.00280000	0.00000000
178	948.17120000	0.00000000	0.00000000
179	948.20040000	0.73650000	0.00000000
180	961.45640000	0.00010000	0.00000000
181	961.57710000	0.00990000	0.00000000
182	962.53620000	0.00120000	0.00000000
183	962.62510000	0.41970000	0.00000000
184	977.79010000	2.06180000	0.00000000
185	997.77310000	0.12140000	0.00000000
186	997.78260000	0.05250000	0.00000000
187	997.78660000	0.08330000	0.00000000
188	997.79560000	0.04590000	0.00000000

189	1000.19730000	0.24940000	0.00000000
190	1000.20420000	0.18120000	0.00000000
191	1000.23990000	0.00500000	0.00000000
192	1000.24710000	0.00760000	0.00000000
193	1015.39660000	0.01860000	0.00000000
194	1015.39710000	0.01010000	0.00000000
195	1015.39820000	0.03050000	0.00000000
196	1015.39880000	0.01180000	0.00000000
197	1015.42230000	0.61900000	0.00000000
198	1016.16510000	0.00000000	0.00000000
199	1016.52600000	0.92490000	0.00000000
200	1017.27300000	0.22310000	0.00000000
201	1037.20750000	56.23890000	0.00000000
202	1037.36220000	0.00000000	0.00000000
203	1050.24530000	0.18390000	0.00000000
204	1050.28460000	0.51260000	0.00000000
205	1051.81460000	17.14580000	0.00000000
206	1052.04360000	0.00080000	0.00000000
207	1066.53950000	5.14460000	0.00000000
208	1078.41760000	152.25750000	0.00000000
209	1078.92400000	8.45790000	0.00000000
210	1078.97170000	7.44280000	0.00000000
211	1079.67020000	17.08500000	0.00000000
212	1079.69620000	0.21050000	0.00000000
213	1086.62980000	0.00000000	0.00000000
214	1087.82660000	0.40170000	0.00000000
215	1111.48630000	31.09950000	0.00000000
216	1116.90840000	3.27340000	0.00000000
217	1120.50040000	552.34920000	0.00000000
218	1121.71600000	8.44420000	0.00000000
219	1121.72670000	5.40970000	0.00000000
220	1125.01590000	0.00000000	0.00000000
221	1125.96520000	0.14900000	0.00000000
222	1126.75380000	0.00390000	0.00000000
223	1126.91220000	103.74630000	0.00000000
224	1127.26060000	20.92960000	0.00000000
225	1127.27480000	62.08450000	0.00000000
226	1144.06940000	0.00000000	0.00000000
227	1149.97250000	18.23960000	0.00000000
228	1164.60440000	0.01890000	0.00000000
229	1165.56880000	0.44600000	0.00000000
230	1183.26480000	2.90490000	0.00000000
231	1183.27230000	4.53470000	0.00000000
232	1185.51010000	4.48810000	0.00000000
233	1185.55010000	0.00990000	0.00000000
234	1199.40630000	14.54910000	0.00000000
235	1208.15100000	45.39710000	0.00000000
236	1212.09200000	0.00000000	0.00000000
237	1232.34760000	12.57530000	0.00000000
238	1232.34870000	9.28440000	0.00000000
239	1233.66530000	128.83800000	0.00000000
240	1233.90380000	0.00060000	0.00000000
241	1250.41170000	0.05410000	0.00000000
242	1250.43430000	0.00030000	0.00000000
243	1250.93700000	22.27070000	0.00000000
244	1250.98140000	9.22580000	0.00000000
245	1262.26400000	4664.56410000	0.00000000
246	1264.14100000	7.63840000	0.00000000
247	1264.24150000	1.11240000	0.00000000
248	1264.47980000	0.02580000	0.00000000
249	1264.82510000	554.91420000	0.00000000
250	1267.45240000	0.01840000	0.00000000
251	1303.86960000	0.00000000	0.00000000
252	1308.84880000	49.69730000	0.00000000

253	1308.86240000	238.40110000	0.00000000
254	1309.10650000	0.37300000	0.00000000
255	1309.11560000	298.70180000	0.00000000
256	1310.96140000	89.52590000	0.00000000
257	1310.96850000	61.57190000	0.00000000
258	1312.93870000	868.18880000	0.00000000
259	1313.14880000	0.02040000	0.00000000
260	1313.73930000	749.72210000	0.00000000
261	1318.56160000	0.17300000	0.00000000
262	1324.59990000	0.00000000	0.00000000
263	1331.48560000	0.00480000	0.00000000
264	1338.91470000	43.70670000	0.00000000
265	1349.11140000	0.30390000	0.00000000
266	1369.89360000	132.31920000	0.00000000
267	1372.71280000	0.16290000	0.00000000
268	1372.72990000	0.05120000	0.00000000
269	1373.04490000	0.00070000	0.00000000
270	1373.24430000	2.16030000	0.00000000
271	1385.91980000	49.70450000	0.00000000
272	1390.62950000	0.00000000	0.00000000
273	1400.79080000	0.00000000	0.00000000
274	1407.59140000	4.57010000	0.00000000
275	1413.73560000	57.53480000	0.00000000
276	1413.74310000	2.24290000	0.00000000
277	1413.80610000	97.86230000	0.00000000
278	1413.80900000	78.36450000	0.00000000
279	1428.68050000	6.22430000	0.00000000
280	1428.68210000	23.69780000	0.00000000
281	1428.85470000	78.89540000	0.00000000
282	1428.85690000	55.76780000	0.00000000
283	1443.75650000	134.88040000	0.00000000
284	1443.85630000	70.72290000	0.00000000
285	1445.18210000	21.41330000	0.00000000
286	1445.25320000	0.03610000	0.00000000
287	1449.39810000	0.02440000	0.00000000
288	1453.34040000	665.73270000	0.00000000
289	1473.81240000	0.00000000	0.00000000
290	1479.25780000	125.83500000	0.00000000
291	1490.31140000	0.56560000	0.00000000
292	1490.33660000	0.00020000	0.00000000
293	1490.61980000	5.64350000	0.00000000
294	1490.67520000	5.62540000	0.00000000
295	1506.33500000	0.55990000	0.00000000
296	1511.53380000	246.46810000	0.00000000
297	1521.43710000	101.22440000	0.00000000
298	1521.44650000	235.99220000	0.00000000
299	1521.66640000	298.26520000	0.00000000
300	1521.79200000	0.00140000	0.00000000
301	1522.98860000	180.97240000	0.00000000
302	1533.60130000	111.21920000	0.00000000
303	1533.61390000	194.47970000	0.00000000
304	1537.10810000	0.00000000	0.00000000
305	1539.03550000	91.65690000	0.00000000
306	1539.19220000	0.01740000	0.00000000
307	1579.28190000	431.95170000	0.00000000
308	1580.50410000	0.00030000	0.00000000
309	1580.54300000	0.99830000	0.00000000
310	1580.54520000	12.96870000	0.00000000
311	1580.72640000	23.55450000	0.00000000
312	1583.67330000	126.41580000	0.00000000
313	1583.67600000	33.65490000	0.00000000
314	1584.28850000	0.00030000	0.00000000
315	1584.31390000	16.74530000	0.00000000
316	1592.42660000	0.00000000	0.00000000

317	1597.54080000	8.07330000	0.00000000
318	1598.52980000	322.76120000	0.00000000
319	1600.12910000	0.19300000	0.00000000
320	1610.00940000	6.02780000	0.00000000
321	1611.68830000	16.64760000	0.00000000
322	1613.98600000	28.02000000	0.00000000
323	1614.53340000	76.56600000	0.00000000
324	1615.35610000	0.00280000	0.00000000
325	1615.43800000	33.87390000	0.00000000
326	1620.06360000	0.00000000	0.00000000
327	1655.25630000	13.57210000	0.00000000
328	1655.25910000	24.94610000	0.00000000
329	1656.54470000	14.33020000	0.00000000
330	1656.56000000	0.00410000	0.00000000
331	3185.70620000	13.87290000	0.00000000
332	3185.70930000	21.77150000	0.00000000
333	3185.74740000	13.46260000	0.00000000
334	3185.75000000	3.09610000	0.00000000
335	3186.89610000	14.78850000	0.00000000
336	3186.90050000	18.28570000	0.00000000
337	3186.94490000	7.89780000	0.00000000
338	3186.94960000	4.01690000	0.00000000
339	3203.86350000	0.13910000	0.00000000
340	3203.86380000	0.13580000	0.00000000
341	3203.86430000	0.14550000	0.00000000
342	3203.86460000	0.13930000	0.00000000
343	3205.75970000	0.00010000	0.00000000
344	3205.92450000	23.50720000	0.00000000
345	3206.03000000	7.75160000	0.00000000
346	3206.05130000	0.00730000	0.00000000
347	3216.12130000	8.08130000	0.00000000
348	3216.12170000	7.24020000	0.00000000
349	3216.13760000	5.63860000	0.00000000
350	3216.13870000	0.30360000	0.00000000
351	3218.50780000	0.01280000	0.00000000
352	3218.55600000	0.00000000	0.00000000
353	3218.94540000	0.16100000	0.00000000
354	3219.04730000	6.43820000	0.00000000
355	3224.47830000	25.04860000	0.00000000
356	3224.54190000	0.00010000	0.00000000
357	3224.68960000	0.32480000	0.00000000
358	3224.76540000	7.77920000	0.00000000
359	3226.54200000	6.62730000	0.00000000
360	3226.54640000	3.58980000	0.00000000
361	3226.54900000	5.15150000	0.00000000
362	3226.55280000	4.60950000	0.00000000
363	3230.07100000	9.26820000	0.00000000
364	3230.08240000	9.56010000	0.00000000
365	3230.31170000	0.97500000	0.00000000
366	3230.32920000	0.14000000	0.00000000

S15. CALCULATIONS ON 2^{2+} (^{1}A) AT 2^{3+} (^{2}A) STRUCTURE IN MeCN (SINGLE POINT)

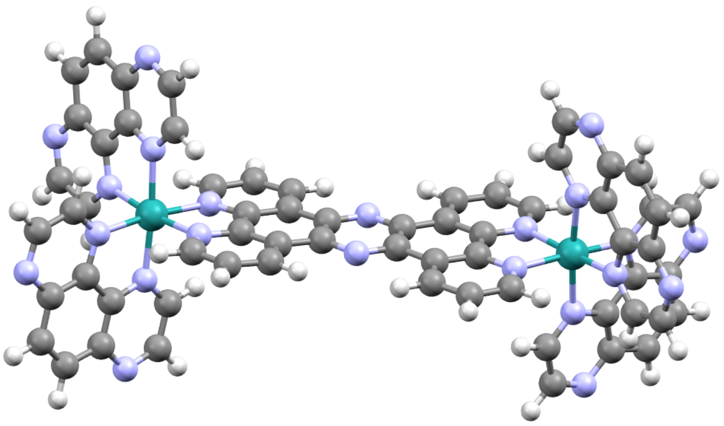

Route	: # b3lyp/genecp scrf=(solvent=acetonitrile) nosymm geom=connectivity em	
	: piricaldispersion=gd3bj int=ultrafine pop=regular	
SMILES	:	
Formula	: $C_{64}H_{36}N_{22}Ru_2^{2+}$	
Charge	: 2	
Multiplicity	: 1	
Energy	: -3856.18021022	a.u.

S15.1. Cartesian Co-ordinates (XYZ format)

124

Ru	6.44484091	0.00000400	0.00005000
N	4.83778095	-0.06668300	1.33466399
N	-0.00000700	0.00050600	1.42044902
N	0.00002000	0.00028700	-1.42044902
N	4.83781195	0.06700000	-1.33457994
C	4.88182020	-0.11967700	2.66391492
H	5.86098289	-0.15057200	3.12003899
C	3.71653390	-0.13962699	3.44479990
H	3.80752707	-0.18497400	4.52146387
C	2.48286009	-0.10167100	2.83123803
H	1.56552899	-0.11671000	3.40335298
C	2.40521908	-0.04500600	1.42525899
C	1.15003395	-0.00932400	0.70856702
C	-1.15003300	0.01022500	0.70854402
C	-3.62319207	0.02838100	0.71078300
C	-3.62317705	-0.02788700	-0.71084601
C	-2.40520501	-0.04522600	-1.42525101
C	-1.15002000	-0.00943400	-0.70856500
C	1.15004694	0.01011500	-0.70854503
C	2.40524793	0.04566800	-1.42521501
C	2.48291993	0.10232200	-2.83119297
H	1.56560099	0.11747800	-3.40332294
C	3.71660900	0.14011900	-3.44473505

H	3.80762601	0.18544801	-4.52139616
C	4.88187885	0.11999900	-2.66383004
H	5.86105490	0.15074199	-3.11993694
C	3.62320495	0.02827200	-0.71078700
C	3.62318993	-0.02777800	0.71085101
C	7.43202019	2.43024111	1.21966600
C	5.84333420	3.03361702	-0.31920400
C	7.63081408	3.77640510	1.57489896
C	8.17556095	1.40268004	1.85123801
C	6.04930305	4.37870979	0.05147000
H	5.12693310	2.76013589	-1.08034301
C	9.13005543	1.71591198	2.83595300
H	5.46974277	5.15241623	-0.43887001
C	8.63964844	-0.83183098	2.06196094
C	9.59807205	-0.50277603	3.04290104
H	8.45900440	-1.85790098	1.77471304
H	10.16552734	-1.29942799	3.51006198
N	7.92496395	0.11832200	1.46921599
N	6.53528690	2.06022596	0.26199701
C	8.17529678	-1.40301001	-1.85113096
C	8.63983536	0.83140898	-2.06183195
C	9.12973404	-1.71642494	-2.83584309
C	7.43154812	-2.43042493	-1.21956801
C	9.59820366	0.50217003	-3.04276490
H	8.45939159	1.85751295	-1.77457702
C	7.63007689	-3.77662706	-1.57480705
H	10.16582203	1.29871297	-3.50991392
C	5.84273195	-3.03349090	0.31928799
C	6.04843807	-4.37862301	-0.05138900
H	5.12637997	-2.75987196	1.08042300
H	5.46871996	-5.15221691	0.43894300
N	7.92495394	-0.11860400	-1.46910095
N	6.53488398	-2.06023502	-0.26190099
C	-4.88186312	0.12040300	2.66381407
C	-4.88180399	-0.12008200	-2.66389894
C	-2.40523410	0.04588800	1.42520702
C	-2.48290396	0.10275600	2.83117700
H	-1.56558299	0.11800000	3.40330195
C	-2.48284411	-0.10210500	-2.83122206
H	-1.56551194	-0.11723200	-3.40333200
C	-3.71659207	0.14064300	3.44471407
C	-3.71651697	-0.14015201	-3.44478011
H	-3.80760789	0.18613701	4.52136898
H	-5.86103821	0.15121301	3.11991906
H	-3.80750895	-0.18566300	-4.52143621
H	-5.86096621	-0.15104300	-3.12001991
N	-4.83779812	0.06720300	1.33457196
N	-4.83776712	-0.06688600	-1.33465505
Ru	-6.44482613	0.00000400	-0.00005000
N	-7.92494011	-0.11837900	1.46912003
N	-6.53488016	-2.06019592	0.26220599
N	-7.92495012	0.11809700	-1.46923494
N	-6.53528214	2.06018710	-0.26230201
C	-8.17530823	-1.40272903	1.85132098
C	-8.63979912	0.83172703	2.06172800
C	-7.43156624	-2.43024206	1.21990705
C	-5.84272480	-3.03354096	-0.31883201
C	-8.17557144	1.40240002	-1.85142803
C	-8.63961220	-0.83214802	-2.06185699
C	-7.43203783	2.43005800	-1.22000504
C	-5.84332705	3.03366709	0.31874800
C	-9.12975502	-1.71599495	2.83607006
C	-9.59817219	0.50263798	3.04270697
H	-8.45933819	1.85778797	1.77433300

C	-7.63011885	-3.77639198	1.57532895
C	-6.04845619	-4.37861919	0.05202700
H	-5.12635088	-2.76003408	-1.07998705
C	-9.13007641	1.71548200	-2.83617997
C	-9.59803963	-0.50324303	-3.04284406
H	-8.45895004	-1.85817599	-1.77446997
C	-7.63085604	3.77617097	-1.57542098
C	-6.04932117	4.37870598	-0.05210800
H	-5.12690401	2.76029897	1.07990599
H	-10.16576862	1.29925501	3.50975490
H	-5.46873713	-5.15228796	-0.43818501
H	-10.16547394	-1.29997003	-3.50990200
H	-5.46975899	5.15248823	0.43811199
C	9.32243633	-3.09400606	-3.18834496
H	10.05894947	-3.31655908	-3.94918299
C	8.60232067	-4.08123207	-2.58531594
H	8.74185944	-5.12198114	-2.84659410
C	8.60311508	4.08082294	2.58541012
H	8.74285793	5.12154484	2.84668398
C	9.32302856	3.09345698	3.18844795
H	10.05958080	3.31586790	3.94929099
C	-8.60317612	4.08043623	-2.58595800
H	-8.74294090	5.12111998	-2.84737110
C	-9.32307911	3.09297609	-3.18885708
H	-10.05964184	3.31527090	-3.94972301
C	-8.60238266	-4.08084488	2.58586407
H	-8.74194241	-5.12155581	2.84728193
C	-9.32248592	-3.09352493	3.18875408
H	-10.05900955	-3.31596303	3.94961500
N	-6.91748810	-4.75775290	0.97288299
N	-9.84644032	-0.73508102	3.43407607
N	6.91744423	-4.75789499	-0.97221202
N	9.84644222	-0.73560500	-3.43397593
N	9.84655762	0.73495299	3.43410206
N	6.91837883	4.75781298	0.97229600
N	-9.84655666	0.73442900	-3.43420291
N	-6.91842318	4.75767088	-0.97296602

S16. CALCULATIONS ON 2^{3+} (^{2}A) AT 2^{4+} (^{1}A) STRUCTURE IN WATER (EXPLICIT + PCM) (SINGLE POINT)

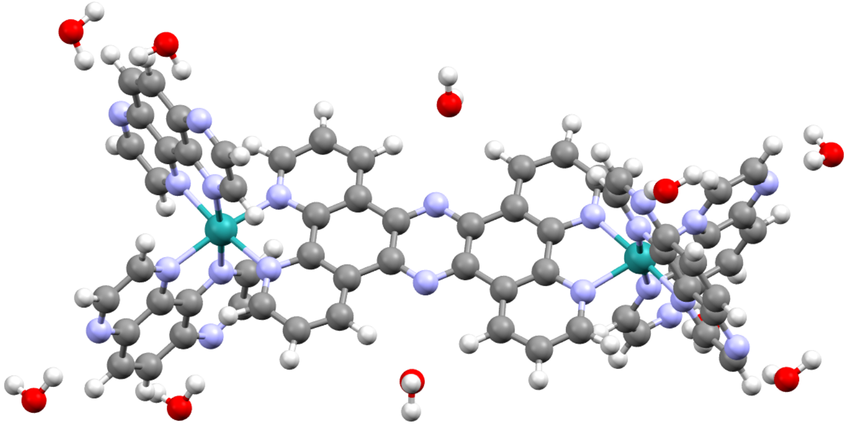

Route	: # b3lyp/genecp scrf=(solvent=water) geom=connectivity empiricaldispers	
	: ion=gd3bj int=ultrafine pop=regular	
SMILES	:	
Formula	: $C_{64}H_{56}N_{22}O_{10}Ru_2^{3+,2}$	
Charge	: 3	
Multiplicity	: 2	
Energy	: -4620.74877714	a.u.

S16.1. Cartesian Co-ordinates (XYZ format)

154

Ru	6.42092419	0.00522900	-0.00710900
N	4.80899191	0.09715600	-1.33583903
N	-0.00569900	0.04637800	-1.40217102
N	-0.00611900	-0.05435100	1.38514304
N	4.80868196	-0.08886400	1.32099104
C	4.85822296	0.17437001	-2.66919994
H	5.84035587	0.21651600	-3.11723900
C	3.70483708	0.20495000	-3.45403504
H	3.80206609	0.26742500	-4.52920294
C	2.46034193	0.15670501	-2.85283995
H	1.55159199	0.17918099	-3.44515204
C	2.39435601	0.07710600	-1.45264995
C	1.13695300	0.02906800	-0.71932203
C	-1.14856195	0.01440300	-0.72027099
C	-3.60412407	-0.00190800	-0.73070699
C	-3.60440397	-0.01808300	0.71208799
C	-2.40623593	-0.03676400	1.43772995
C	-1.14880395	-0.02713400	0.70276600
C	1.13674402	-0.03271500	0.70279002
C	2.39395404	-0.07631900	1.43670499
C	2.45958591	-0.15523300	2.83694100
H	1.55051005	-0.18015800	3.42877412

C	3.70393610	-0.19890800	3.43876004
H	3.80084109	-0.26024100	4.51402521
C	4.85756016	-0.16515200	2.65441489
H	5.83962584	-0.20402101	3.10289693
C	3.59274793	-0.04084500	0.71247101
C	3.59293294	0.04531300	-0.72790003
C	7.42012119	-2.39667702	-1.27423799
C	5.83126020	-3.04139805	0.24922000
C	7.63022184	-3.73214197	-1.65947902
C	8.16079903	-1.35339701	-1.88199306
C	6.04403210	-4.37779999	-0.14669301
H	5.11257887	-2.79085398	1.01554501
C	9.11818409	-1.64645803	-2.86903310
H	5.47108507	-5.16687107	0.32488999
C	8.61612225	0.88829303	-2.04611802
C	9.57893944	0.58575100	-3.03094196
H	8.43164158	1.90754402	-1.73930895
H	10.14587688	1.39111400	-3.48173499
N	7.90484524	-0.07783000	-1.47592199
N	6.51834202	-2.05292296	-0.31185600
C	8.15975857	1.36531699	1.86763203
C	8.61576939	-0.87615401	2.03287196
C	9.11693001	1.65915895	2.85466504
C	7.41862488	2.40803504	1.25946605
C	9.57836819	-0.57282001	3.01764798
H	8.43155003	-1.89560497	1.72654796
C	7.62857294	3.74379206	1.64374399
H	10.14470005	-1.37789905	3.46971607
C	5.82917213	3.05140090	-0.26394999
C	6.04168415	4.38810396	0.13112800
H	5.11036015	2.80020690	-1.02994394
H	5.46834898	5.17672777	-0.34073201
N	7.90434217	0.08949500	1.46204102
N	6.51678801	2.06346297	0.29741400
C	-4.86997509	0.00540000	-2.67588401
C	-4.87119818	-0.02979200	2.65668797
C	-2.40567803	0.01926800	-1.45578694
C	-2.47208810	0.04075200	-2.85812593
H	-1.56333494	0.06362400	-3.45068908
C	-2.47332597	-0.05973800	2.84001708
H	-1.56505704	-0.08019500	3.43319511
C	-3.71671891	0.03349100	-3.46104908
C	-3.71827793	-0.05620600	3.44238091
H	-3.81406999	0.04834200	-4.53791285
H	-5.85243702	-0.00800200	-3.12506104
H	-3.81603599	-0.07211100	4.51919794
H	-5.85404778	-0.01859500	3.10508299
N	-4.82020807	-0.01225000	-1.34048796
N	-4.82076502	-0.01052600	1.32136500
Ru	-6.43255520	-0.00878600	-0.00957200
N	-7.91363621	0.17570700	-1.47225904
N	-6.52037716	2.06567192	-0.18212301
N	-7.91667080	-0.18575300	1.45072305
N	-6.53289890	-2.08272099	0.16103800
C	-8.15723705	1.47560203	-1.80200195
C	-8.63180447	-0.74826300	-2.10079789
C	-7.41282606	2.47435689	-1.12766302
C	-5.83174706	3.01314998	0.44396800
C	-8.17246628	-1.48459303	1.77522004
C	-8.63006306	0.74211001	2.07900596
C	-7.43313408	-2.48722100	1.10105896
C	-5.84912300	-3.03381205	-0.46494099
C	-9.10721684	1.83560503	-2.77393389
C	-9.58827686	-0.37853900	-3.06881595

H	-8.45706940	-1.78576899	-1.85570300
C	-7.61134911	3.83248711	-1.43134797
C	-6.03320980	4.37291384	0.12999199
H	-5.12009716	2.71191502	1.19849098
C	-9.13166332	-1.83997405	2.73978209
C	-9.59543419	0.37712401	3.03990793
H	-8.44512272	1.77888703	1.83826494
C	-7.64607191	-3.84475207	1.39747202
C	-6.06481409	-4.39299583	-0.15792599
H	-5.13106918	-2.73586297	-1.21468699
H	-10.16112804	-1.15018404	-3.56848907
H	-5.45909119	5.12740088	0.65392101
H	-10.16407490	1.15190399	3.53955412
H	-5.49438381	-5.15047407	-0.68155700
C	9.32039833	3.02666402	3.23830605
H	10.06311989	3.22077298	4.00199890
C	8.60408592	4.02899981	2.65657902
H	8.74106216	5.06701899	2.93270802
C	8.60507298	-4.01644087	-2.67320299
H	8.74146938	-5.05414677	-2.95079994
C	9.32196426	-3.01369095	-3.25349498
H	10.06256294	-3.20686007	-4.01947021
C	-8.62166500	-4.19284010	2.39033604
H	-8.76512718	-5.24648523	2.59442902
C	-9.33749962	-3.22898889	3.03428102
H	-10.08302498	-3.47075891	3.78143191
C	-8.57690716	4.18519497	-2.43234611
H	-8.70417404	5.23884916	-2.64700007
C	-9.29774857	3.22511911	-3.07637191
H	-10.03140640	3.47006392	-3.83412290
N	-6.89723921	4.78313017	-0.78092700
N	-9.82446003	0.87603599	-3.40743208
N	6.91583586	4.73663092	1.05801797
N	9.82812786	0.65773302	3.42729402
N	9.82894993	-0.64450401	-3.44128394
N	6.91789722	-4.72551298	-1.07415700
N	-9.84518623	-0.87664700	3.37175608
N	-6.93801498	-4.79921722	0.74602503
O	-0.00487500	-0.15036000	4.95248795
O	-0.00056400	0.19740000	-4.96508312
H	-0.02721800	-0.87218201	5.59056997
H	0.04280300	0.64835602	5.48963690
H	-0.07853500	0.99923497	-5.49392796
H	0.00367800	-0.51974797	-5.60879517
O	11.68879986	-1.93453503	-5.29005384
O	7.90683413	-7.21594191	-2.24134707
O	7.90243292	7.22760677	2.22622395
O	11.51379490	1.92784500	5.44945478
O	-11.63531590	2.29289699	-5.21442413
O	-7.86582184	7.34869289	-1.79211104
O	-11.54655170	-2.27424312	5.29622793
O	-7.79959679	-7.35565805	1.87316597
H	-10.91159630	-2.38196611	6.01280689
H	-7.24053001	-7.38023996	2.65741706
H	-12.34457684	2.50620794	-4.59827805
H	-8.64835644	7.45099115	-1.23948097
H	8.68353271	-7.35336685	-1.68812203
H	10.87195492	1.98756802	6.16553020
H	8.67890930	7.36721277	1.67323005
H	12.38525867	-2.17674494	-4.66998386
H	7.42506504	6.51446486	1.76632297
H	11.12423420	-1.33561397	-4.76997089
H	7.42825890	-6.50293016	-1.78250206
H	11.07545662	1.34459996	4.80482483

H	-7.39437914	6.60458088	-1.37736297
H	-11.09021664	1.65763104	-4.71725416
H	-7.42802811	-6.61887217	1.35636604
H	-11.10199165	-1.65032601	4.69544315

S17. CALCULATIONS ON 2^{3+} (^{2}A) IN WATER (EXPLICIT + PCM)

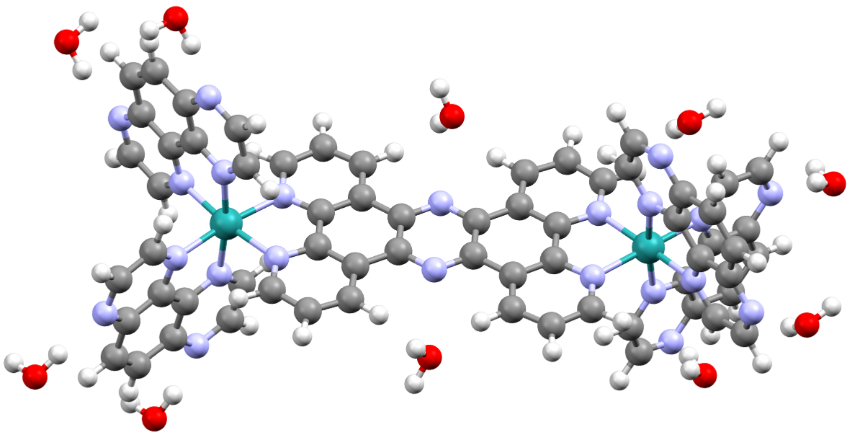

Route	:	# opt freq b3lyp/genecp scrf=(solvent=water) geom=connectivity empiric
	:	aldispersion=gd3bj int=ultrafine pop=regular
SMILES	:	c1cc2c3c(c4ccc[n+]5c4c2[n+](c1)[Ru]567([n+]8ccnc9c8c1[n+]6ccnc1cc9)[n+]1ccnc2c1c1[n+]7ccnc1cc2)nc1c2ccc[n+]4c2c2c(c1n3)ccc[n+]2[Ru]412([n+]3ccnc4c3c3[n+]1ccnc3cc4)[n+]1ccnc3c1c1[n+]2ccnc1cc3.O.O.O.O.O.O.O.O.O.O
Formula	:	$C_{64}H_{56}N_{22}O_{10}Ru_2^{3+,2}$
Charge	:	3
Multiplicity	:	2
Energy	:	-4620.75312519 a.u.
Gibbs Energy	:	-4619.73697700 a.u.
Number of imaginary frequencies	:	3

S17.1. Cartesian Co-ordinates (XYZ format)

154

Ru	6.39870691	-0.01265500	-0.00169200
N	4.77979088	0.11514600	-1.32366502
N	-0.03491300	0.07781000	-1.39089406
N	-0.03435700	-0.20163999	1.38437796
N	4.78138208	-0.19326200	1.31705594
C	4.82814789	0.25098500	-2.65247703
H	5.81267881	0.31117499	-3.09385610
C	3.67476106	0.31717500	-3.43590689
H	3.77138901	0.42770499	-4.50733614
C	2.43047810	0.24349800	-2.83623505
H	1.51788104	0.29602799	-3.41834092
C	2.36460209	0.09710200	-1.44239604
C	1.10798097	0.01295600	-0.71183997
C	-1.17750001	0.00758400	-0.71103901
C	-3.63307691	0.01461500	-0.72041100
C	-3.63360500	-0.12434900	0.71573400
C	-2.43468189	-0.20183299	1.43651700
C	-1.17739904	-0.13297600	0.70486999

C	1.10834503	-0.13205799	0.70514202
C	2.36594391	-0.20753901	1.43519402
C	2.43361998	-0.35892200	2.82842708
H	1.52164102	-0.42381400	3.41031909
C	3.67869210	-0.42134199	3.42779803
H	3.77652907	-0.53625399	4.49866009
C	4.83126879	-0.33572099	2.64498997
H	5.81673622	-0.38354701	3.08590293
C	3.56458998	-0.12557000	0.71372700
C	3.56390405	0.03184800	-0.72055602
C	7.56773615	-2.32554007	-1.28500402
C	5.92367315	-3.09955597	0.13111700
C	7.88428879	-3.65035892	-1.65929401
C	8.23726368	-1.23557901	-1.86232698
C	6.25119877	-4.40687513	-0.24663600
H	5.13869810	-2.90438199	0.84837800
C	9.23569965	-1.43985200	-2.83811307
H	5.70903397	-5.23940182	0.18387701
C	8.48431683	1.04841006	-2.06371999
C	9.47064495	0.82885498	-3.02368093
H	8.20126629	2.04931688	-1.77012801
H	9.96730614	1.67530298	-3.48112392
N	7.85678291	0.02574400	-1.46415102
N	6.58110380	-2.05141091	-0.36910000
C	8.18884182	1.27972102	1.86025596
C	8.53041744	-0.99318999	2.05431604
C	9.18109226	1.52150595	2.83424091
C	7.47430420	2.34275603	1.28769302
C	9.51054478	-0.73636401	3.01083302
H	8.28706360	-2.00407100	1.75925505
C	7.73473120	3.67833996	1.66797805
H	10.04273796	-1.56290996	3.46462893
C	5.79378891	3.05352712	-0.11935900
C	6.06503820	4.37121820	0.26531801
H	5.01515007	2.82911992	-0.83491099
H	5.48538685	5.18192387	-0.15784000
N	7.85866880	0.00444300	1.46011698
N	6.49841785	2.03158307	0.37138799
C	-4.89779186	0.19857500	-2.65799904
C	-4.89886189	-0.30687499	2.65301609
C	-2.43439388	0.07876100	-1.44250798
C	-2.49992990	0.20949900	-2.83876610
H	-1.59083200	0.26348901	-3.42687511
C	-2.50059390	-0.33967501	2.83204103
H	-1.59160995	-0.40298301	3.41942501
C	-3.74395609	0.26810899	-3.43993497
C	-3.74486995	-0.39342001	3.43324399
H	-3.84064198	0.36827800	-4.51225805
H	-5.88035297	0.23888300	-3.10536098
H	-3.84167910	-0.50073302	4.50488186
H	-5.88101006	-0.34072301	3.10195589
N	-4.84856987	0.07300100	-1.32828903
N	-4.84983301	-0.17129400	1.32416105
Ru	-6.45966721	-0.01429900	-0.00126200
N	-7.92828083	0.35710299	-1.44025302
N	-6.49997711	2.06932592	0.04619500
N	-7.96110916	-0.30781901	1.42490995
N	-6.62427998	-2.08950591	-0.06298800
C	-8.15017796	1.68938398	-1.62275195
C	-8.65340233	-0.47953200	-2.17390394
C	-7.38885880	2.59471107	-0.84362203
C	-5.79069090	2.93055606	0.76689398
C	-8.28339767	-1.62367404	1.57419503
C	-8.63728523	0.56371200	2.16477203

C	-7.56995201	-2.56674695	0.79465002
C	-5.95705700	-2.98583794	-0.78110403
C	-9.09115696	2.17019701	-2.55006003
C	-9.59922409	0.01041900	-3.09786296
H	-8.49179554	-1.54007494	-2.04764199
C	-7.56398916	3.98115206	-0.99568099
C	-5.96982193	4.31979895	0.60569799
H	-5.07984209	2.53604603	1.47807002
C	-9.27821732	-2.05333209	2.47007394
C	-9.63685226	0.12545200	3.05762196
H	-8.39686680	1.61212599	2.06621194
C	-7.84539986	-3.93990111	0.91555202
C	-6.23563290	-4.36171103	-0.65001899
H	-5.20295906	-2.62970901	-1.46746898
H	-10.17836475	-0.69191098	-3.68486500
H	-5.38032579	5.00261497	1.20526898
H	-10.17451763	0.85579199	3.64988399
H	-5.67765713	-5.07352018	-1.24607694
C	9.45493507	2.88657403	3.19979095
H	10.22700691	3.05184388	3.94095612
C	8.76552963	3.91878390	2.64060211
H	8.95640564	4.94872379	2.91497302
C	8.92121124	-3.85159707	-2.63411188
H	9.15458393	-4.87379408	-2.90463209
C	9.56562328	-2.79360700	-3.19894099
H	10.33932877	-2.92943192	-3.94430494
C	-8.86355972	-4.36437988	1.83316195
H	-9.05534840	-5.42783499	1.90152502
C	-9.55336761	-3.45717597	2.57967997
H	-10.32637978	-3.75665903	3.27624607
C	-8.52482414	4.45836687	-1.94847906
H	-8.63447094	5.53127384	-2.04515290
C	-9.26016712	3.58761406	-2.69495296
H	-9.98898315	3.92714000	-3.42022800
N	-6.83111811	4.84216976	-0.24864000
N	-9.81828213	1.29856896	-3.29056811
N	7.02312994	4.69186592	1.14072394
N	9.84799290	0.50204402	3.40654397
N	9.85745716	-0.39523199	-3.41665196
N	7.21841383	-4.69073677	-1.12376595
N	-9.95643997	-1.14641094	3.21441603
N	-7.15452099	-4.83727598	0.17120400
O	-0.13350099	-0.59019101	5.03773403
O	-0.13799500	0.44772401	-5.04818678
H	0.26819500	-1.39750397	5.37746906
H	0.28175300	0.11656200	5.54429913
H	0.25630501	1.26228797	-5.37920713
H	0.28681600	-0.24990501	-5.55946589
O	11.85976410	-1.43102300	-5.19620514
O	8.41832256	-7.10548592	-2.14508104
O	8.10460091	7.15115690	2.18460011
O	11.63623905	1.60359895	5.36256886
O	-11.61503220	2.93041897	-4.91939878
O	-7.75445700	7.51878786	-0.97146302
O	-11.76987362	-2.69184589	4.90877819
O	-8.12266827	-7.47177315	0.99126297
H	-11.16448879	-2.92215610	5.62196016
H	-7.58633900	-7.59670591	1.78179395
H	-12.31974220	3.07830501	-4.27929497
H	-8.53499317	7.57396317	-0.40937001
H	9.19099331	-7.14422607	-1.57113099
H	11.01154137	1.74015999	6.08294010
H	8.87131500	7.23934889	1.60817003
H	12.55259609	-1.62449801	-4.55559778

H	7.61042690	6.40864277	1.78553700
H	11.19863319	-0.94093198	-4.66771507
H	7.88657093	-6.38542414	-1.75357103
H	11.11430931	1.09635496	4.70897293
H	-7.29594278	6.72594881	-0.64094800
H	-11.07432079	2.23724008	-4.50101900
H	-7.71609497	-6.69656706	0.56504202
H	-11.27695465	-2.02043295	4.40466404

S17.2. Frequencies

Mode	IR frequency	IR intensity	Raman intensity
1	-10.18990000	1.23750000	0.00000000
2	-7.04300000	0.03320000	0.00000000
3	-4.87170000	4.27850000	0.00000000
4	7.49770000	2.10900000	0.00000000
5	10.42780000	7.40480000	0.00000000
6	11.57270000	14.43360000	0.00000000
7	13.97990000	9.03590000	0.00000000
8	17.18350000	3.04630000	0.00000000
9	18.95730000	37.93750000	0.00000000
10	19.03060000	2.96750000	0.00000000
11	20.69500000	0.43580000	0.00000000
12	22.44630000	6.94090000	0.00000000
13	24.49490000	1.94060000	0.00000000
14	36.09010000	17.21900000	0.00000000
15	38.11760000	81.29090000	0.00000000
16	39.49580000	8.81580000	0.00000000
17	40.18980000	25.96830000	0.00000000
18	41.92000000	1.41780000	0.00000000
19	43.22640000	37.22390000	0.00000000
20	44.89530000	74.48150000	0.00000000
21	46.14250000	5.25950000	0.00000000
22	46.79840000	2.27350000	0.00000000
23	48.59870000	0.88160000	0.00000000
24	52.77900000	0.35300000	0.00000000
25	60.26000000	178.40490000	0.00000000
26	64.16390000	48.64310000	0.00000000
27	65.34750000	2.57080000	0.00000000
28	67.02240000	3.16760000	0.00000000
29	67.82610000	22.49720000	0.00000000
30	69.09650000	38.64640000	0.00000000
31	70.84590000	52.25950000	0.00000000
32	72.42170000	12.46890000	0.00000000
33	75.15240000	3.28000000	0.00000000
34	76.67000000	15.20460000	0.00000000
35	81.45660000	9.54750000	0.00000000
36	82.75230000	13.08350000	0.00000000
37	84.62410000	2.62030000	0.00000000
38	85.55180000	10.09920000	0.00000000
39	89.96230000	21.95830000	0.00000000
40	92.43740000	0.82340000	0.00000000
41	96.98220000	18.12230000	0.00000000
42	98.09270000	1.87670000	0.00000000
43	102.14490000	17.35770000	0.00000000
44	102.61310000	40.70610000	0.00000000
45	104.50210000	62.14170000	0.00000000
46	109.26910000	86.01090000	0.00000000
47	124.02380000	38.08980000	0.00000000
48	129.61630000	1.97230000	0.00000000
49	134.35180000	0.91140000	0.00000000
50	138.73670000	1.93330000	0.00000000
51	139.64090000	8.20280000	0.00000000
52	145.34510000	110.12250000	0.00000000
53	147.29040000	0.98570000	0.00000000
54	148.97070000	14.41410000	0.00000000
55	151.96060000	1069.35430000	0.00000000
56	153.64130000	33.83120000	0.00000000
57	165.63200000	847.27430000	0.00000000
58	175.92220000	4.97130000	0.00000000
59	178.01650000	428.75290000	0.00000000
60	179.45480000	22.28770000	0.00000000

61	181.37330000	354.97460000	0.00000000
62	182.41900000	415.81010000	0.00000000
63	183.51590000	216.78640000	0.00000000
64	188.29550000	1650.33010000	0.00000000
65	190.10940000	4.97530000	0.00000000
66	192.67430000	80.48180000	0.00000000
67	192.86130000	31.62240000	0.00000000
68	195.56100000	4.53850000	0.00000000
69	200.07500000	98.69990000	0.00000000
70	202.32370000	225.51690000	0.00000000
71	204.13330000	558.28680000	0.00000000
72	205.66360000	143.08440000	0.00000000
73	207.63260000	165.29910000	0.00000000
74	209.32680000	20.41870000	0.00000000
75	212.23770000	20.46780000	0.00000000
76	214.36220000	9.03450000	0.00000000
77	215.57880000	122.16160000	0.00000000
78	217.64060000	11.43920000	0.00000000
79	228.74820000	455.54130000	0.00000000
80	236.74470000	27.99540000	0.00000000
81	238.67830000	325.92320000	0.00000000
82	239.41720000	67.25930000	0.00000000
83	239.47880000	149.31690000	0.00000000
84	239.71990000	380.40990000	0.00000000
85	243.74650000	17.51630000	0.00000000
86	245.22380000	13.61780000	0.00000000
87	255.50150000	817.40880000	0.00000000
88	258.21160000	140.86150000	0.00000000
89	258.78480000	58.71340000	0.00000000
90	260.60990000	1.34320000	0.00000000
91	261.61390000	4.06610000	0.00000000
92	261.78620000	5.21060000	0.00000000
93	275.25480000	2608.49030000	0.00000000
94	284.40400000	27.01850000	0.00000000
95	287.36850000	6.66520000	0.00000000
96	289.86630000	2.31950000	0.00000000
97	292.13560000	240.33120000	0.00000000
98	296.22900000	14.90160000	0.00000000
99	299.89670000	27.85960000	0.00000000
100	301.05240000	11.28420000	0.00000000
101	306.26360000	3.43960000	0.00000000
102	319.10380000	10.94850000	0.00000000
103	320.92520000	32.30280000	0.00000000
104	324.26420000	10.52100000	0.00000000
105	336.15550000	80.47750000	0.00000000
106	339.09560000	47.67150000	0.00000000
107	340.44310000	9.19330000	0.00000000
108	341.48250000	18.02560000	0.00000000
109	342.08940000	90.72280000	0.00000000
110	346.05250000	122.10620000	0.00000000
111	350.66440000	159.78240000	0.00000000
112	351.66310000	18.29800000	0.00000000
113	352.76720000	24.37070000	0.00000000
114	369.65350000	92.49920000	0.00000000
115	374.42010000	96.82350000	0.00000000
116	380.87450000	30.06310000	0.00000000
117	383.67520000	195.55480000	0.00000000
118	389.61140000	27.81920000	0.00000000
119	393.91050000	28.42210000	0.00000000
120	432.16830000	6227.38820000	0.00000000
121	440.70540000	3.43030000	0.00000000
122	442.83880000	3.46630000	0.00000000
123	447.01960000	1.38820000	0.00000000
124	447.96570000	0.35530000	0.00000000

125	448.62900000	0.14560000	0.00000000
126	450.55590000	5.17370000	0.00000000
127	451.71550000	0.08770000	0.00000000
128	453.48330000	1.26530000	0.00000000
129	458.18290000	18.65030000	0.00000000
130	459.05010000	8.94810000	0.00000000
131	465.68710000	2.77790000	0.00000000
132	468.16170000	1.64970000	0.00000000
133	473.19230000	38.12750000	0.00000000
134	477.22700000	5.54180000	0.00000000
135	478.36460000	262.47210000	0.00000000
136	481.18050000	363.38880000	0.00000000
137	487.35190000	23.01640000	0.00000000
138	488.69620000	6.70260000	0.00000000
139	492.42380000	69.98350000	0.00000000
140	496.99460000	353.46530000	0.00000000
141	499.38200000	1.04190000	0.00000000
142	505.88890000	0.45210000	0.00000000
143	509.56590000	8.06470000	0.00000000
144	537.46940000	18.09440000	0.00000000
145	548.03180000	2740.82540000	0.00000000
146	554.95000000	2.75670000	0.00000000
147	558.32060000	1.50730000	0.00000000
148	562.03650000	87.35000000	0.00000000
149	562.41920000	24.34550000	0.00000000
150	562.66400000	93.70210000	0.00000000
151	563.33930000	32.26070000	0.00000000
152	563.63960000	25.05970000	0.00000000
153	569.10260000	2.34220000	0.00000000
154	571.97050000	6673.64550000	0.00000000
155	572.33750000	35.33400000	0.00000000
156	574.88820000	0.15650000	0.00000000
157	583.83370000	8.67700000	0.00000000
158	584.74490000	10.73000000	0.00000000
159	586.47180000	49.06030000	0.00000000
160	587.08550000	64.96230000	0.00000000
161	589.02660000	18.62210000	0.00000000
162	594.29710000	47.16160000	0.00000000
163	595.25220000	12.86750000	0.00000000
164	596.59800000	236.02980000	0.00000000
165	599.69930000	37.51050000	0.00000000
166	622.62190000	4003.08920000	0.00000000
167	627.94410000	3194.08490000	0.00000000
168	640.53650000	8.58040000	0.00000000
169	643.65430000	12.98680000	0.00000000
170	647.27470000	0.04060000	0.00000000
171	657.61380000	13667.00470000	0.00000000
172	659.56530000	1421.09040000	0.00000000
173	661.74860000	161.92040000	0.00000000
174	662.87980000	50.70470000	0.00000000
175	666.29440000	1092.07290000	0.00000000
176	668.32140000	2086.75820000	0.00000000
177	671.43230000	148.23070000	0.00000000
178	674.00830000	1599.07130000	0.00000000
179	674.76780000	10567.95730000	0.00000000
180	680.33270000	80.40950000	0.00000000
181	681.31750000	42.63540000	0.00000000
182	687.20730000	814.86370000	0.00000000
183	689.20610000	231.42580000	0.00000000
184	720.39540000	51.07460000	0.00000000
185	731.51770000	106.09990000	0.00000000
186	732.18310000	39.19380000	0.00000000
187	735.22810000	943.05670000	0.00000000
188	742.39050000	5.10700000	0.00000000

189	744.02290000	257.42060000	0.00000000
190	745.90050000	623.45730000	0.00000000
191	746.31210000	12.98850000	0.00000000
192	746.48660000	36.70260000	0.00000000
193	747.08400000	206.88890000	0.00000000
194	752.20530000	240.20180000	0.00000000
195	753.09920000	111.13230000	0.00000000
196	754.59960000	0.65600000	0.00000000
197	754.79750000	0.26950000	0.00000000
198	758.16680000	93.19980000	0.00000000
199	761.99680000	120.11630000	0.00000000
200	764.21930000	46.95550000	0.00000000
201	767.84070000	14.71120000	0.00000000
202	768.35760000	29.42940000	0.00000000
203	771.89900000	1.75250000	0.00000000
204	772.92820000	1.78130000	0.00000000
205	773.88460000	148.90950000	0.00000000
206	822.34220000	0.15560000	0.00000000
207	825.03490000	167.40700000	0.00000000
208	827.58230000	5.35840000	0.00000000
209	828.37490000	11.17660000	0.00000000
210	828.76800000	1.87750000	0.00000000
211	832.97270000	0.04830000	0.00000000
212	841.68200000	35.81980000	0.00000000
213	842.07440000	542.02470000	0.00000000
214	844.47180000	86.67000000	0.00000000
215	848.55100000	50.91320000	0.00000000
216	849.69140000	72.93670000	0.00000000
217	852.64830000	4.91280000	0.00000000
218	853.28570000	0.35670000	0.00000000
219	854.72460000	0.36400000	0.00000000
220	862.44800000	0.31980000	0.00000000
221	869.77780000	28.50460000	0.00000000
222	871.03320000	23.40700000	0.00000000
223	874.31580000	30.02400000	0.00000000
224	880.70560000	0.35090000	0.00000000
225	882.45800000	0.37760000	0.00000000
226	883.34790000	9.89750000	0.00000000
227	892.67770000	41685.80410000	0.00000000
228	907.50110000	203.08630000	0.00000000
229	908.17350000	109.12950000	0.00000000
230	920.04240000	73.32610000	0.00000000
231	921.13260000	71.76830000	0.00000000
232	926.40700000	4.76140000	0.00000000
233	927.13890000	1972.12820000	0.00000000
234	928.00250000	4.39500000	0.00000000
235	928.43280000	6.04700000	0.00000000
236	945.77970000	49.52530000	0.00000000
237	946.62340000	71.07870000	0.00000000
238	948.90190000	216.85570000	0.00000000
239	950.23760000	190.35700000	0.00000000
240	951.94260000	1.86270000	0.00000000
241	967.55650000	60.55670000	0.00000000
242	968.09300000	42.08300000	0.00000000
243	969.59310000	3.94450000	0.00000000
244	970.39870000	14.81990000	0.00000000
245	988.92530000	0.09720000	0.00000000
246	992.61660000	0.23030000	0.00000000
247	992.94410000	10.21480000	0.00000000
248	995.56650000	10.46090000	0.00000000
249	997.49500000	0.25380000	0.00000000
250	999.50940000	0.25410000	0.00000000
251	1000.04640000	0.30190000	0.00000000
252	1001.42920000	0.44180000	0.00000000

253	1045.67310000	837.48540000	0.00000000
254	1049.68500000	277.40660000	0.00000000
255	1051.61700000	33.15970000	0.00000000
256	1056.77210000	2.33520000	0.00000000
257	1057.82290000	0.76480000	0.00000000
258	1063.18690000	0.07210000	0.00000000
259	1065.77510000	53.23160000	0.00000000
260	1068.23440000	0.89120000	0.00000000
261	1068.48000000	5.05200000	0.00000000
262	1070.39180000	37.12900000	0.00000000
263	1073.46820000	4.29370000	0.00000000
264	1074.21640000	0.35820000	0.00000000
265	1077.51900000	1.21390000	0.00000000
266	1078.13730000	0.61490000	0.00000000
267	1078.32970000	0.01240000	0.00000000
268	1079.71520000	0.74570000	0.00000000
269	1080.99480000	8.80070000	0.00000000
270	1082.13940000	3.34180000	0.00000000
271	1089.44550000	41.99910000	0.00000000
272	1097.55040000	197.63520000	0.00000000
273	1098.96410000	167.66100000	0.00000000
274	1108.46790000	9.71720000	0.00000000
275	1118.53550000	443.42700000	0.00000000
276	1122.96190000	797.48080000	0.00000000
277	1128.06710000	50.32360000	0.00000000
278	1128.38370000	34.57990000	0.00000000
279	1130.09040000	31.51640000	0.00000000
280	1130.27740000	325.44810000	0.00000000
281	1130.50120000	588.68490000	0.00000000
282	1133.40570000	22.67740000	0.00000000
283	1137.61580000	3.93350000	0.00000000
284	1137.68840000	5.86990000	0.00000000
285	1142.87990000	0.15680000	0.00000000
286	1153.40970000	16.21870000	0.00000000
287	1158.39080000	223.76080000	0.00000000
288	1165.78860000	18.88530000	0.00000000
289	1169.24330000	4.21800000	0.00000000
290	1181.49650000	261.50900000	0.00000000
291	1190.38380000	902.75760000	0.00000000
292	1195.50420000	0.40480000	0.00000000
293	1196.89190000	0.07720000	0.00000000
294	1205.36350000	1599.47270000	0.00000000
295	1208.39740000	1540.86680000	0.00000000
296	1215.93600000	14.01740000	0.00000000
297	1231.48610000	12.88670000	0.00000000
298	1234.25810000	2.05120000	0.00000000
299	1237.77180000	1.12230000	0.00000000
300	1239.13790000	1.38020000	0.00000000
301	1239.30450000	2.76830000	0.00000000
302	1241.82780000	42.40850000	0.00000000
303	1253.90260000	0.79200000	0.00000000
304	1260.04270000	6.73030000	0.00000000
305	1260.82720000	416.73150000	0.00000000
306	1260.92170000	2.21470000	0.00000000
307	1264.64670000	10.08510000	0.00000000
308	1264.70320000	24.35150000	0.00000000
309	1265.08150000	117.92900000	0.00000000
310	1265.74060000	38.46820000	0.00000000
311	1266.03480000	13.52440000	0.00000000
312	1289.59850000	6.67010000	0.00000000
313	1290.90620000	16.50520000	0.00000000
314	1305.79590000	0.07040000	0.00000000
315	1313.35150000	132.15780000	0.00000000
316	1315.02520000	108.35210000	0.00000000

317	1315.17900000	93.06260000	0.00000000
318	1315.81760000	4.63620000	0.00000000
319	1316.04820000	1.73680000	0.00000000
320	1322.62620000	30.84750000	0.00000000
321	1323.32140000	4.52930000	0.00000000
322	1340.35820000	3.32630000	0.00000000
323	1341.92670000	649.64180000	0.00000000
324	1343.51720000	1596.48910000	0.00000000
325	1353.01560000	4.96700000	0.00000000
326	1359.05350000	13.89290000	0.00000000
327	1367.97480000	1.64160000	0.00000000
328	1376.64430000	0.34230000	0.00000000
329	1377.11290000	0.29580000	0.00000000
330	1390.42980000	371.77900000	0.00000000
331	1395.87580000	0.05480000	0.00000000
332	1408.94060000	3974.34890000	0.00000000
333	1415.56910000	14.53380000	0.00000000
334	1415.90660000	4.35560000	0.00000000
335	1423.15750000	120.21250000	0.00000000
336	1423.34390000	35.19330000	0.00000000
337	1426.41630000	5.94400000	0.00000000
338	1431.48450000	40.01540000	0.00000000
339	1431.73940000	47.49090000	0.00000000
340	1432.39990000	905.11400000	0.00000000
341	1433.92250000	586.01260000	0.00000000
342	1434.77470000	152.27500000	0.00000000
343	1438.54980000	91.44590000	0.00000000
344	1443.15710000	108.72290000	0.00000000
345	1444.71330000	24.57220000	0.00000000
346	1456.93060000	22.39090000	0.00000000
347	1470.42880000	3.43240000	0.00000000
348	1476.11400000	30.08060000	0.00000000
349	1486.71300000	0.24110000	0.00000000
350	1494.02610000	2.23230000	0.00000000
351	1494.24480000	6.20800000	0.00000000
352	1495.81500000	5.50640000	0.00000000
353	1497.59440000	94.76540000	0.00000000
354	1499.96250000	87.30510000	0.00000000
355	1506.73050000	757.31500000	0.00000000
356	1508.72120000	1004.74460000	0.00000000
357	1510.66020000	11.24910000	0.00000000
358	1510.86900000	20.13290000	0.00000000
359	1518.64570000	635.22370000	0.00000000
360	1520.84880000	54.40160000	0.00000000
361	1524.83670000	237.11930000	0.00000000
362	1525.25800000	88.65750000	0.00000000
363	1532.46700000	69.39600000	0.00000000
364	1533.89470000	164.37660000	0.00000000
365	1536.22240000	50.24030000	0.00000000
366	1537.23340000	138.82460000	0.00000000
367	1540.67080000	107.47610000	0.00000000
368	1541.38870000	310.67440000	0.00000000
369	1541.79360000	45.90970000	0.00000000
370	1547.96540000	17.80470000	0.00000000
371	1562.94230000	239.33160000	0.00000000
372	1585.17070000	13.52180000	0.00000000
373	1585.54500000	9.49780000	0.00000000
374	1586.23970000	10.27690000	0.00000000
375	1587.01960000	72.72150000	0.00000000
376	1587.80770000	21.76820000	0.00000000
377	1603.41910000	0.03590000	0.00000000
378	1611.87350000	10.43170000	0.00000000
379	1616.74850000	50.60400000	0.00000000
380	1618.09980000	27.65360000	0.00000000

381	1618.55080000	31.06340000	0.00000000
382	1619.86020000	0.17330000	0.00000000
383	1621.92150000	66.68230000	0.00000000
384	1623.32470000	2.51050000	0.00000000
385	1625.79990000	0.09830000	0.00000000
386	1632.82030000	0.30720000	0.00000000
387	1638.67920000	2.76790000	0.00000000
388	1647.54700000	11.13410000	0.00000000
389	1648.32390000	15.70870000	0.00000000
390	1650.98020000	26.72770000	0.00000000
391	1658.49200000	187.69880000	0.00000000
392	1659.17790000	72.47550000	0.00000000
393	1660.20780000	145.69890000	0.00000000
394	1661.64230000	45.22010000	0.00000000
395	1662.59030000	20.74820000	0.00000000
396	1663.66000000	36.39020000	0.00000000
397	1664.46770000	159.45470000	0.00000000
398	1665.84060000	115.41270000	0.00000000
399	1666.69730000	186.15340000	0.00000000
400	1667.25580000	129.70730000	0.00000000
401	3183.10440000	107.84870000	0.00000000
402	3183.56230000	149.29950000	0.00000000
403	3187.66110000	100.39460000	0.00000000
404	3188.19170000	69.32720000	0.00000000
405	3191.44680000	9.44420000	0.00000000
406	3191.57940000	10.69650000	0.00000000
407	3193.47180000	35.16060000	0.00000000
408	3193.66380000	63.15190000	0.00000000
409	3193.92920000	35.07880000	0.00000000
410	3194.26430000	63.25080000	0.00000000
411	3195.00610000	80.85730000	0.00000000
412	3195.27630000	25.58800000	0.00000000
413	3196.90960000	6.05920000	0.00000000
414	3197.63960000	7.64070000	0.00000000
415	3197.76770000	4.19090000	0.00000000
416	3197.88930000	3.84230000	0.00000000
417	3201.88050000	4.85800000	0.00000000
418	3201.95760000	2.07040000	0.00000000
419	3202.57030000	9.28050000	0.00000000
420	3203.09850000	9.58800000	0.00000000
421	3212.34930000	4.55170000	0.00000000
422	3212.76730000	4.43870000	0.00000000
423	3213.76430000	0.89840000	0.00000000
424	3213.80350000	5.16130000	0.00000000
425	3221.95140000	72.30030000	0.00000000
426	3224.11220000	57.07820000	0.00000000
427	3225.26680000	65.08250000	0.00000000
428	3225.44330000	9.51520000	0.00000000
429	3226.98730000	19.67470000	0.00000000
430	3227.54880000	4.35810000	0.00000000
431	3228.35960000	16.01160000	0.00000000
432	3228.40970000	3.73560000	0.00000000
433	3233.98040000	6.27630000	0.00000000
434	3234.20540000	0.34590000	0.00000000
435	3235.09320000	3.25480000	0.00000000
436	3238.63080000	3.47920000	0.00000000
437	3512.35770000	8824.89560000	0.00000000
438	3520.13710000	2205.70070000	0.00000000
439	3538.35610000	6292.31340000	0.00000000
440	3543.49440000	715.56780000	0.00000000
441	3615.89150000	579.22040000	0.00000000
442	3616.47550000	273.58350000	0.00000000
443	3617.11520000	1930.77500000	0.00000000
444	3617.83200000	829.84260000	0.00000000

445	3801.41890000	33.16000000	0.00000000
446	3801.64870000	31.25060000	0.00000000
447	3846.20530000	234.32340000	0.00000000
448	3846.44810000	195.30300000	0.00000000
449	3846.53930000	80.75970000	0.00000000
450	3846.66770000	127.87390000	0.00000000
451	3847.15810000	67.89540000	0.00000000
452	3847.16390000	31.77390000	0.00000000
453	3847.26740000	171.80250000	0.00000000
454	3847.65220000	104.24820000	0.00000000
455	3891.68070000	84.78360000	0.00000000
456	3891.72310000	89.18250000	0.00000000

S18. CALCULATIONS ON 2^{2+} (^{1}A) AT 2^{3+} (^{2}A) STRUCTURE IN WATER (EXPLICIT + PCM) (SINGLE POINT)

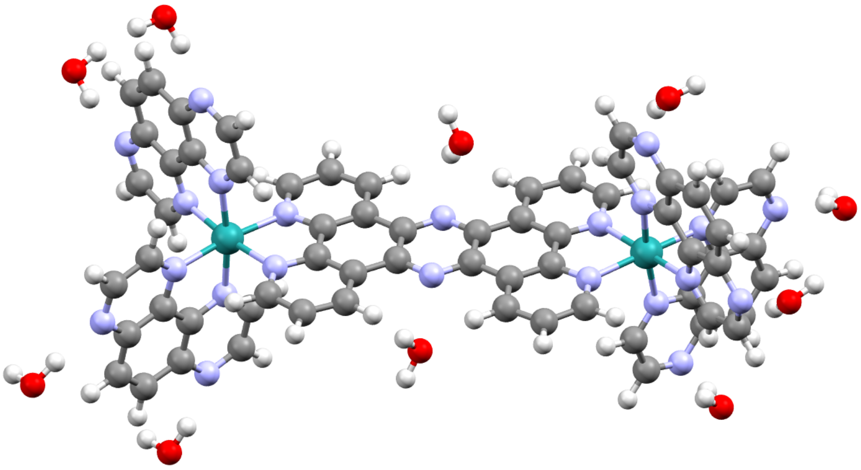

Route	:	# b3lyp/genecp scrf=(solvent=water) geom=connectivity empiricaldispers
	:	ion=gd3bj int=ultrafine pop=regular
SMILES	:	
Formula	:	$C_{64}H_{56}N_{22}O_{10}Ru_2^{2+}$
Charge	:	2
Multiplicity	:	1
Energy	:	-4620.87321672 a.u.

S18.1. Cartesian Co-ordinates (XYZ format)

154

Ru	6.39870691	-0.01265500	-0.00169200
N	4.77979088	0.11514600	-1.32366502
N	-0.03491300	0.07781000	-1.39089406
N	-0.03435700	-0.20163999	1.38437796
N	4.78138208	-0.19326200	1.31705594
C	4.82814789	0.25098500	-2.65247703
H	5.81267881	0.31117499	-3.09385610
C	3.67476106	0.31717500	-3.43590689
H	3.77138901	0.42770499	-4.50733614
C	2.43047810	0.24349800	-2.83623505
H	1.51788104	0.29602799	-3.41834092
C	2.36460209	0.09710200	-1.44239604
C	1.10798097	0.01295600	-0.71183997
C	-1.17750001	0.00758400	-0.71103901
C	-3.63307691	0.01461500	-0.72041100
C	-3.63360500	-0.12434900	0.71573400
C	-2.43468189	-0.20183299	1.43651700
C	-1.17739904	-0.13297600	0.70486999
C	1.10834503	-0.13205799	0.70514202
C	2.36594391	-0.20753901	1.43519402
C	2.43361998	-0.35892200	2.82842708
H	1.52164102	-0.42381400	3.41031909

C	3.67869210	-0.42134199	3.42779803
H	3.77652907	-0.53625399	4.49866009
C	4.83126879	-0.33572099	2.64498997
H	5.81673622	-0.38354701	3.08590293
C	3.56458998	-0.12557000	0.71372700
C	3.56390405	0.03184800	-0.72055602
C	7.56773615	-2.32554007	-1.28500402
C	5.92367315	-3.09955597	0.13111700
C	7.88428879	-3.65035892	-1.65929401
C	8.23726368	-1.23557901	-1.86232698
C	6.25119877	-4.40687513	-0.24663600
H	5.13869810	-2.90438199	0.84837800
C	9.23569965	-1.43985200	-2.83811307
H	5.70903397	-5.23940182	0.18387701
C	8.48431683	1.04841006	-2.06371999
C	9.47064495	0.82885498	-3.02368093
H	8.20126629	2.04931688	-1.77012801
H	9.96730614	1.67530298	-3.48112392
N	7.85678291	0.02574400	-1.46415102
N	6.58110380	-2.05141091	-0.36910000
C	8.18884182	1.27972102	1.86025596
C	8.53041744	-0.99318999	2.05431604
C	9.18109226	1.52150595	2.83424091
C	7.47430420	2.34275603	1.28769302
C	9.51054478	-0.73636401	3.01083302
H	8.28706360	-2.00407100	1.75925505
C	7.73473120	3.67833996	1.66797805
H	10.04273796	-1.56290996	3.46462893
C	5.79378891	3.05352712	-0.11935900
C	6.06503820	4.37121820	0.26531801
H	5.01515007	2.82911992	-0.83491099
H	5.48538685	5.18192387	-0.15784000
N	7.85866880	0.00444300	1.46011698
N	6.49841785	2.03158307	0.37138799
C	-4.89779186	0.19857500	-2.65799904
C	-4.89886189	-0.30687499	2.65301609
C	-2.43439388	0.07876100	-1.44250798
C	-2.49992990	0.20949900	-2.83876610
H	-1.59083200	0.26348901	-3.42687511
C	-2.50059390	-0.33967501	2.83204103
H	-1.59160995	-0.40298301	3.41942501
C	-3.74395609	0.26810899	-3.43993497
C	-3.74486995	-0.39342001	3.43324399
H	-3.84064198	0.36827800	-4.51225805
H	-5.88035297	0.23888300	-3.10536098
H	-3.84167910	-0.50073302	4.50488186
H	-5.88101006	-0.34072301	3.10195589
N	-4.84856987	0.07300100	-1.32828903
N	-4.84983301	-0.17129400	1.32416105
Ru	-6.45966721	-0.01429900	-0.00126200
N	-7.92828083	0.35710299	-1.44025302
N	-6.49997711	2.06932592	0.04619500
N	-7.96110916	-0.30781901	1.42490995
N	-6.62427998	-2.08950591	-0.06298800
C	-8.15017796	1.68938398	-1.62275195
C	-8.65340233	-0.47953200	-2.17390394
C	-7.38885880	2.59471107	-0.84362203
C	-5.79069090	2.93055606	0.76689398
C	-8.28339767	-1.62367404	1.57419503
C	-8.63728523	0.56371200	2.16477203
C	-7.56995201	-2.56674695	0.79465002
C	-5.95705700	-2.98583794	-0.78110403
C	-9.09115696	2.17019701	-2.55006003
C	-9.59922409	0.01041900	-3.09786296

H	-8.49179554	-1.54007494	-2.04764199
C	-7.56398916	3.98115206	-0.99568099
C	-5.96982193	4.31979895	0.60569799
H	-5.07984209	2.53604603	1.47807002
C	-9.27821732	-2.05333209	2.47007394
C	-9.63685226	0.12545200	3.05762196
H	-8.39686680	1.61212599	2.06621194
C	-7.84539986	-3.93990111	0.91555202
C	-6.23563290	-4.36171103	-0.65001899
H	-5.20295906	-2.62970901	-1.46746898
H	-10.17836475	-0.69191098	-3.68486500
H	-5.38032579	5.00261497	1.20526898
H	-10.17451763	0.85579199	3.64988399
H	-5.67765713	-5.07352018	-1.24607694
C	9.45493507	2.88657403	3.19979095
H	10.22700691	3.05184388	3.94095612
C	8.76552963	3.91878390	2.64060211
H	8.95640564	4.94872379	2.91497302
C	8.92121124	-3.85159707	-2.63411188
H	9.15458393	-4.87379408	-2.90463209
C	9.56562328	-2.79360700	-3.19894099
H	10.33932877	-2.92943192	-3.94430494
C	-8.86355972	-4.36437988	1.83316195
H	-9.05534840	-5.42783499	1.90152502
C	-9.55336761	-3.45717597	2.57967997
H	-10.32637978	-3.75665903	3.27624607
C	-8.52482414	4.45836687	-1.94847906
H	-8.63447094	5.53127384	-2.04515290
C	-9.26016712	3.58761406	-2.69495296
H	-9.98898315	3.92714000	-3.42022800
N	-6.83111811	4.84216976	-0.24864000
N	-9.81828213	1.29856896	-3.29056811
N	7.02312994	4.69186592	1.14072394
N	9.84799290	0.50204402	3.40654397
N	9.85745716	-0.39523199	-3.41665196
N	7.21841383	-4.69073677	-1.12376595
N	-9.95643997	-1.14641094	3.21441603
N	-7.15452099	-4.83727598	0.17120400
O	-0.13350099	-0.59019101	5.03773403
O	-0.13799500	0.44772401	-5.04818678
H	0.26819500	-1.39750397	5.37746906
H	0.28175300	0.11656200	5.54429913
H	0.25630501	1.26228797	-5.37920713
H	0.28681600	-0.24990501	-5.55946589
O	11.85976410	-1.43102300	-5.19620514
O	8.41832256	-7.10548592	-2.14508104
O	8.10460091	7.15115690	2.18460011
O	11.63623905	1.60359895	5.36256886
O	-11.61503220	2.93041897	-4.91939878
O	-7.75445700	7.51878786	-0.97146302
O	-11.76987362	-2.69184589	4.90877819
O	-8.12266827	-7.47177315	0.99126297
H	-11.16448879	-2.92215610	5.62196016
H	-7.58633900	-7.59670591	1.78179395
H	-12.31974220	3.07830501	-4.27929497
H	-8.53499317	7.57396317	-0.40937001
H	9.19099331	-7.14422607	-1.57113099
H	11.01154137	1.74015999	6.08294010
H	8.87131500	7.23934889	1.60817003
H	12.55259609	-1.62449801	-4.55559778
H	7.61042690	6.40864277	1.78553700
H	11.19863319	-0.94093198	-4.66771507
H	7.88657093	-6.38542414	-1.75357103
H	11.11430931	1.09635496	4.70897293

H	-7.29594278	6.72594881	-0.64094800
H	-11.07432079	2.23724008	-4.50101900
H	-7.71609497	-6.69656706	0.56504202
H	-11.27695465	-2.02043295	4.40466404

[Note1] Theo Keane: https://github.com/theochemtheo/chemscripts
